# Supplementary material for: Pan-cancer neural regulation pattern with implications for patient stratification and immunotherapy
Source: Mol Ther Nucleic Acids. 2025 Oct 7;36(4):102732. doi: 10.1016/j.omtn.2025.102732 (PMC12554035; doi:10.1016/j.omtn.2025.102732)
Supplement: Document S1. Figures S1–S21 [file mmc1.pdf]

OMTN, Volume *36*

Supplemental information

Pan-cancer neural regulation pattern with implications for patient stratification and immunotherapy

Yueying Gao, Jiyu Guo, Wenyi Yang, Kefan Liu, Qingyi Yang, Si Li, Dezhong Lv, Yangyang Cai, Kang Xu, Weiwei Zhou, Qinghua Jiang, Juan Xu, Yongsheng Li, and Haoxiu Sun

Supplemental Figures

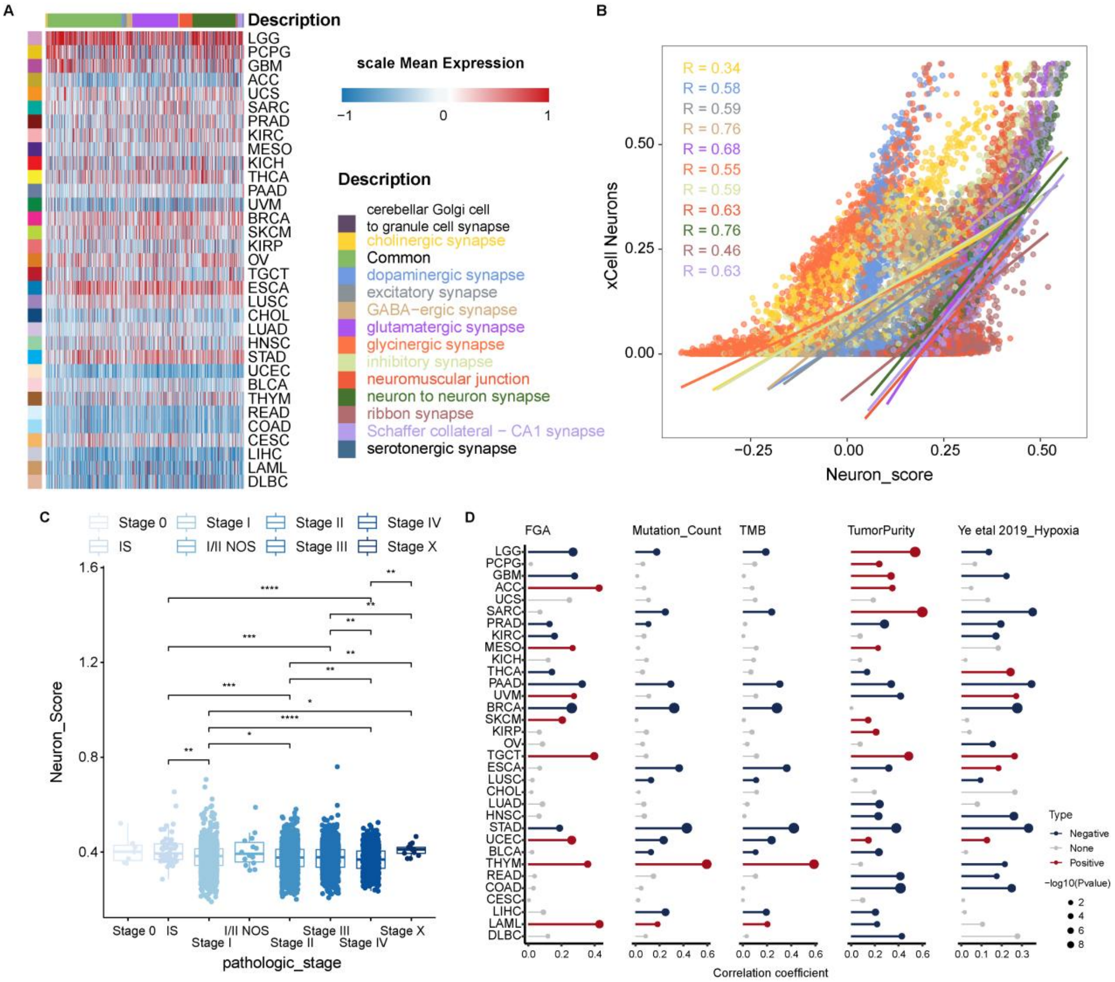

Figure S1. Correlations between the activities of neural signal pathways and clinical features. (A) Heatmap showing the expressions of neural-related genes across 33 cancer types. (B) Scatter plot showing the correlation between neural signals and the abundance of neuronal cells obtained based on xCell algorithm. (C) Boxplot showing the neural signal activitiesacross different stages. (D) Dotplot showing the correlation between the activity of neural signals and clinical parameters, such as FGA, mutation count and TMB.

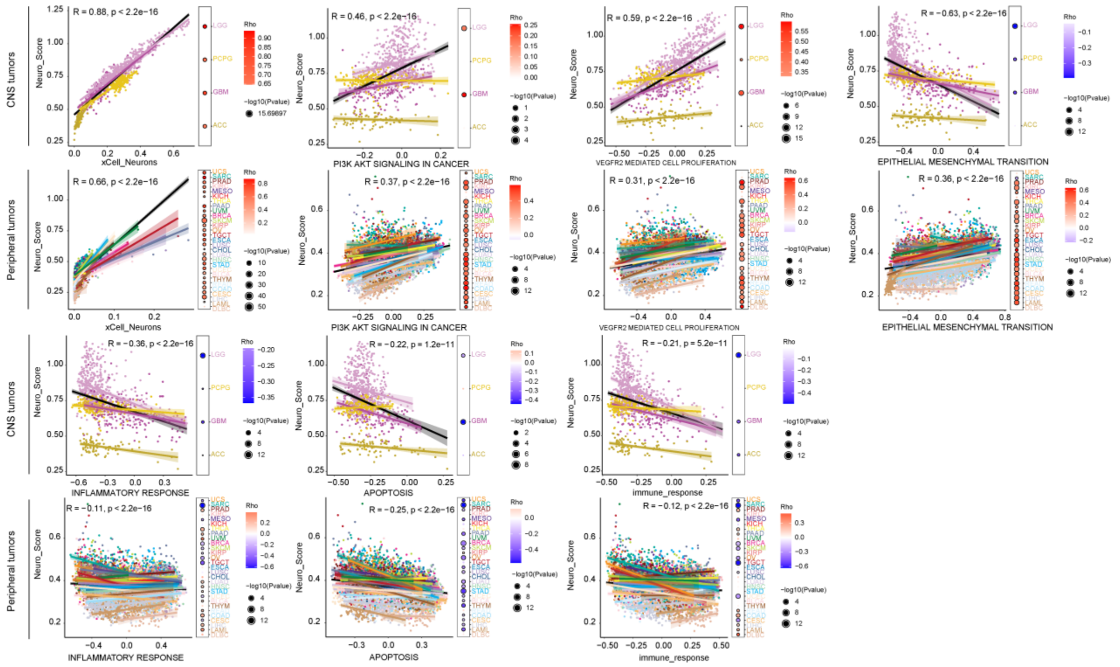

Figure S2. Correlation between the activities of neural signals and TME-related pathways in diverse cancer types. Scatter plot (left) colored by cancer types showing the distribution of patients. Dot plot (right) showing the correlation between neural signals and TME-related pathways. Red represents positive correlation, blue represents negative correlation. The size of the dot represents -log10 (Pvalue).

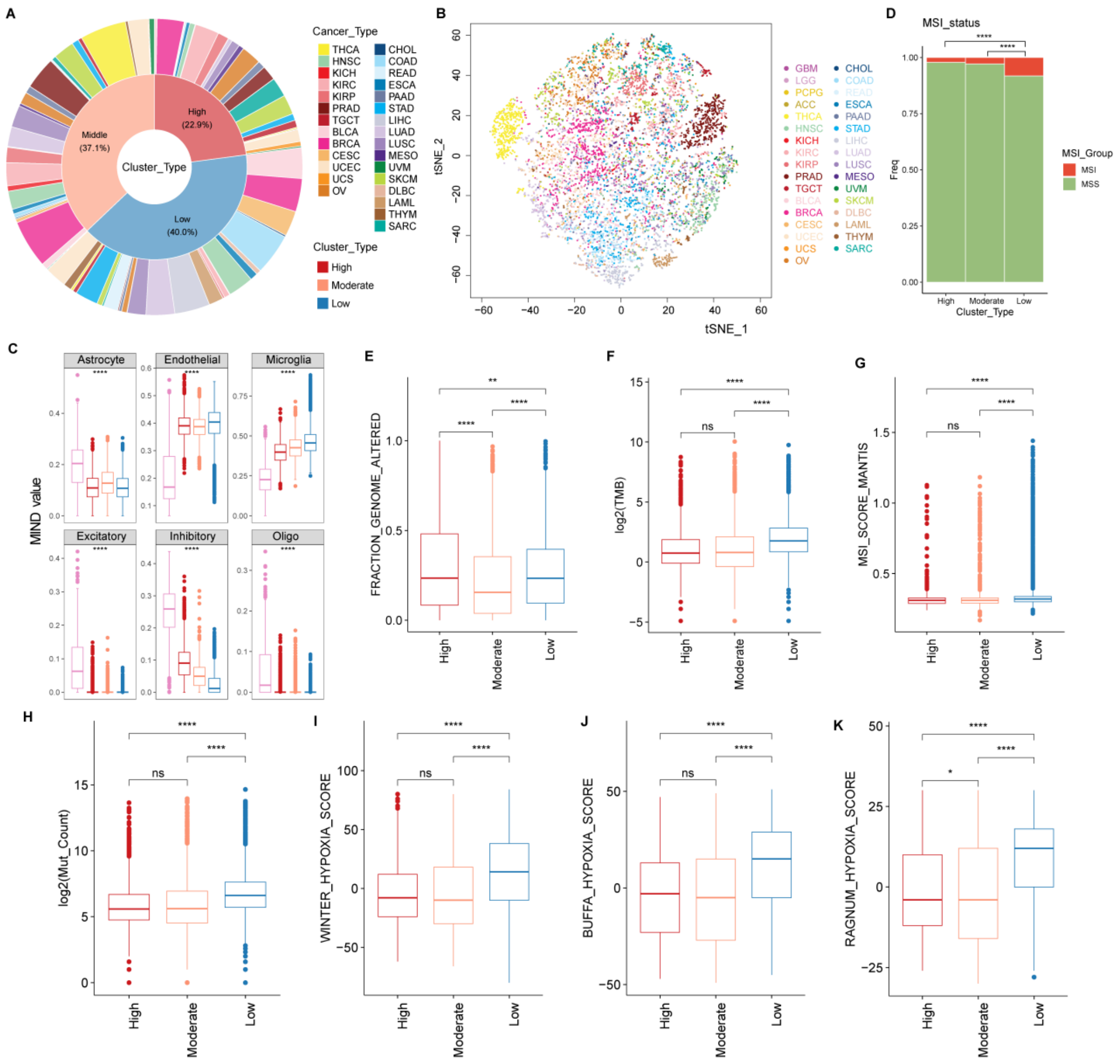

Figure S3. Clinical relevance of molecular subtypes classified by neural signal activity. (A) Circular diagram showing the distribution of cancer types in three molecular subtypes. (B)The t-SNE plot showing the distribution of cancer types. (C) Barplot showing the proportion of patients with microsatellite instability (MSI) or microsatellite stability (MSS) across three subtypes. (D) Boxplot of six cell types derived from deconvolution method across diverse molecular subtypes. (E-K) Boxplot showing clinical features across three subtypes, including FGA (E), TMB (F), MSI score (G), mutation count (H), hypoxia score (I-K).

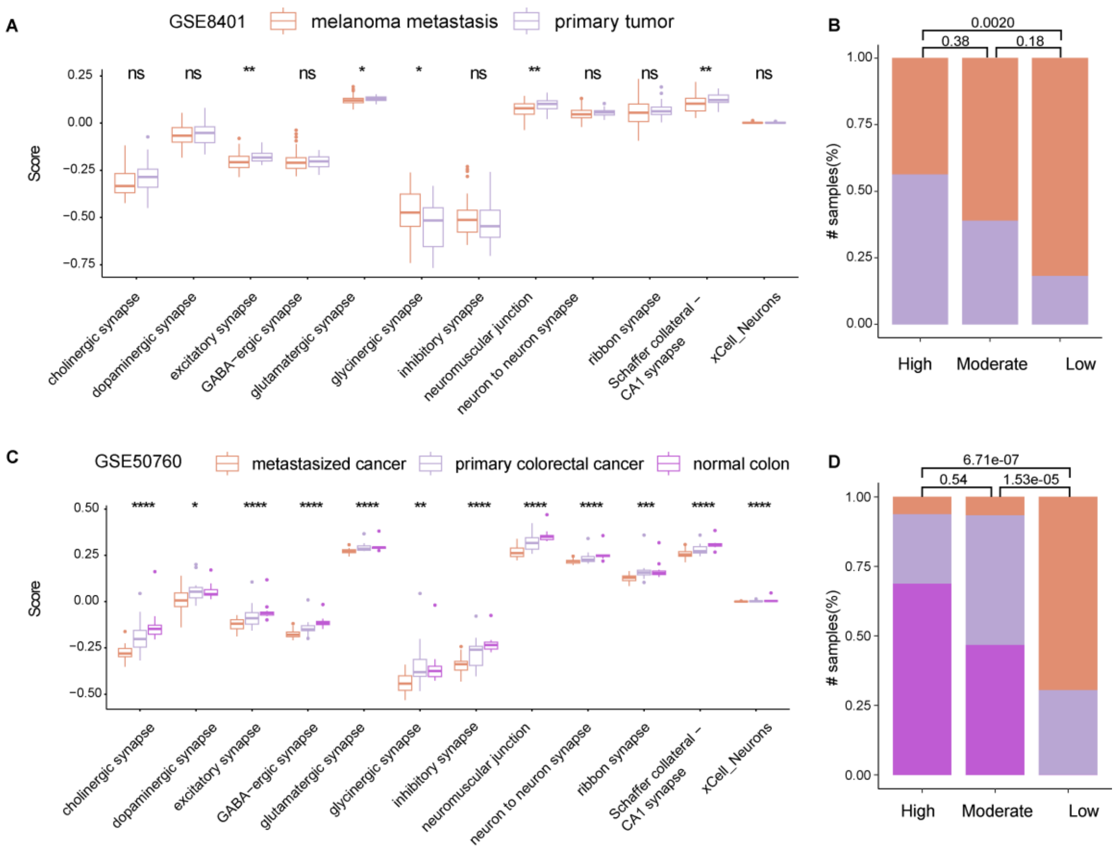

Figure S4. Landscape of metastasis across molecular subtypes. (A-B) Boxplot and Barplot showing the distribution of neural signals in metastasis and primary tumors in melanoma cohort. (C-D) Boxplot and Barplot showing the distribution of neural signals in metastasis and primary tumors in colorectal cohort.

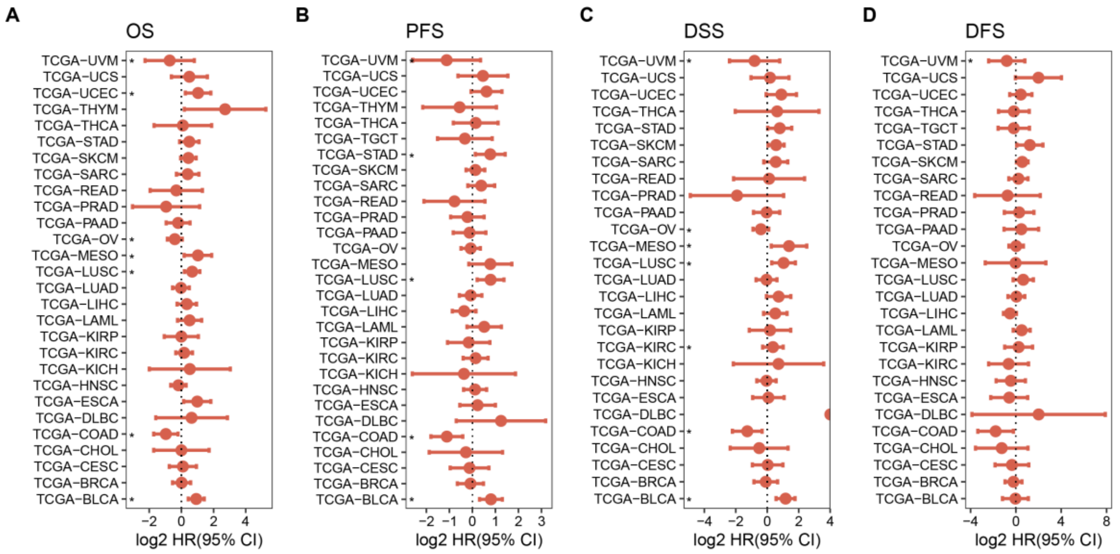

Figure S5. Survival analysis of diverse cancers. Survival analysis was performed to evaluate prognostic value (A, OS, overall survival; B, PFS, progression-free survival; C, DSS, disease-specific survival; D, DFS, disease-free survival) of diverse cancers.

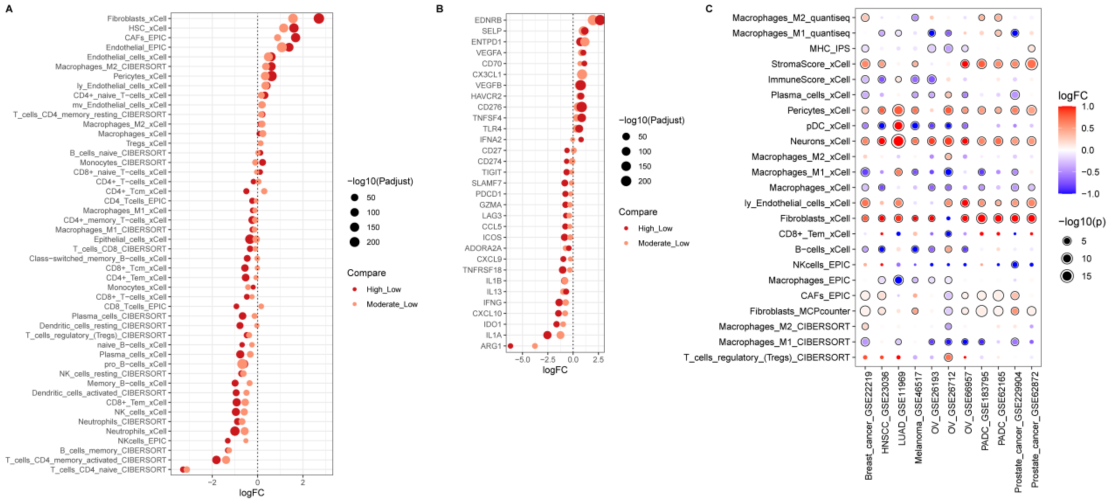

Figure S6. Differences of immunomodulators between molecular subtypes. The dot plot showing the differential changes in abundances of cell types (A) and expressions of immune regulatory gene (B) related to TIME. (C) The relationship between neural signals and TIME in other cohorts. Red represents upregulation in the neural-high group, while blue represents upregulation in the neural-low group. The size of the points are related to the p-value.

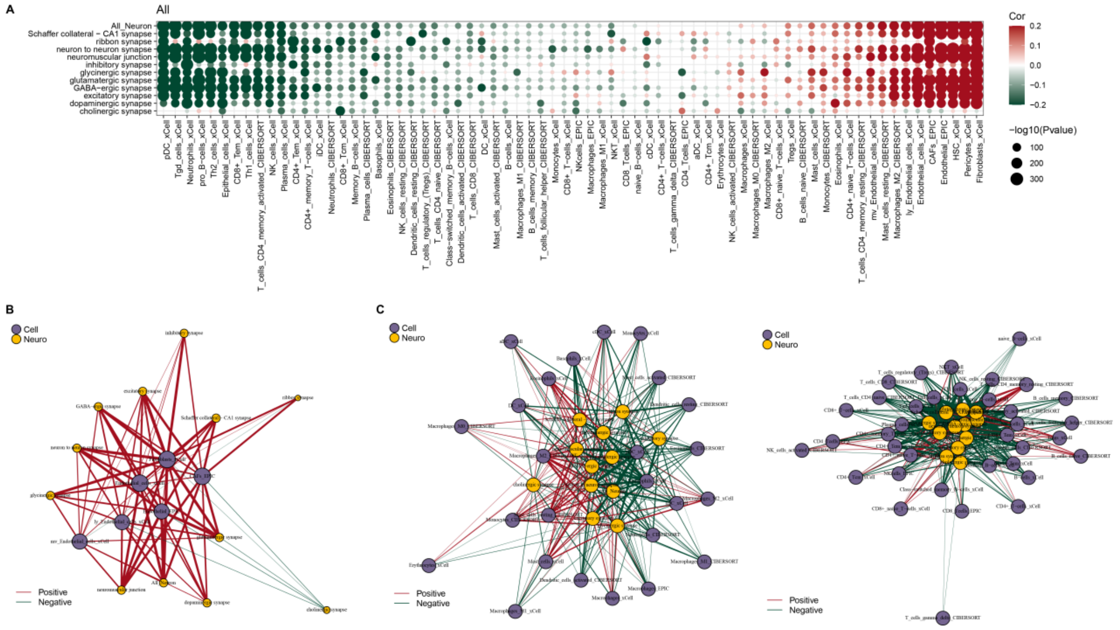

Figure S7. The correlation between neural signals and TIME-related cell types. (A) The dot plot showing the correlation between the activities of neural signals and abundances of TIME-related cell types. (B) The networks exhibiting the association between neural signals and oncogenesis-related cell types. (C) The networks exhibiting the association between neural signals and immune-related cell types, such as myeloid cell (left) and lymphoid cells (right). Red represents significant positive correlation, green represents significant negative correlation. Yellow represents neural signals, while purple represents cancer or immune related cell types.

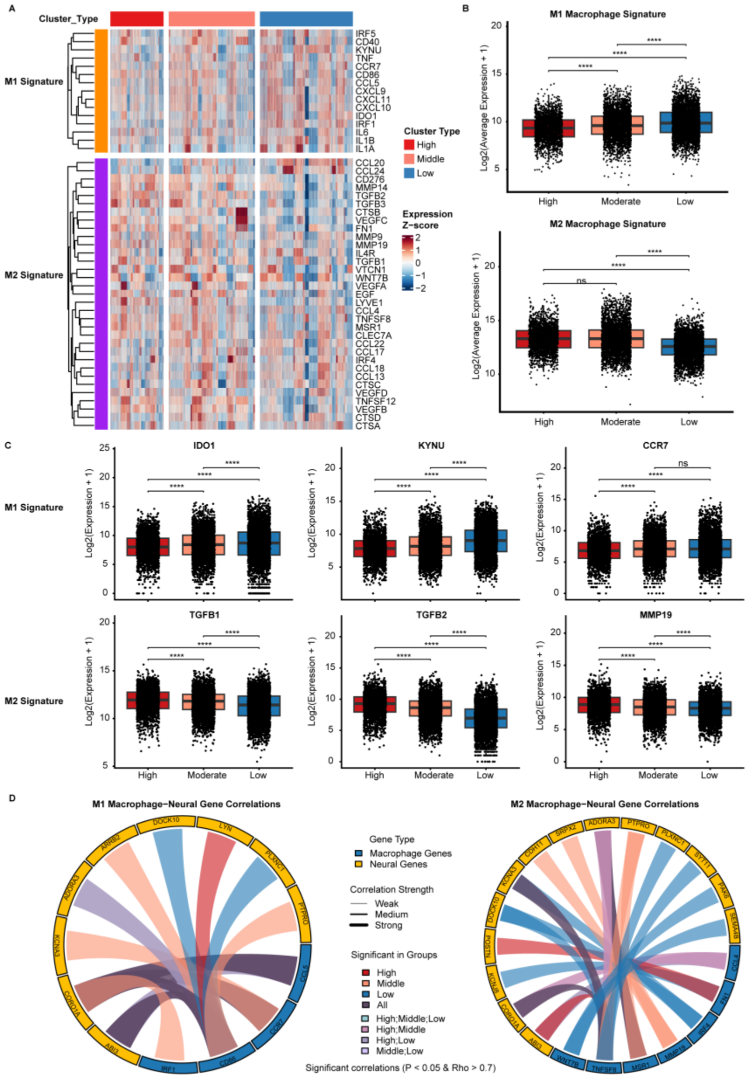

Figure S8. Correlation between neural signals and macrophage subtypes. (A) Heatmap showing the expression of marker genes related macrophage polarization. (B) Boxplot displaying macrophage signature scores across diverse neural subtypes. (C) Boxplot showing the expression distribution of macrophage signatures in different subtypes. (D) Circos plot showing the correlation between neural signals signatures and macrophage signatures. Blue indicates neural signals signatures, and yellow indicates macrophage signatures.

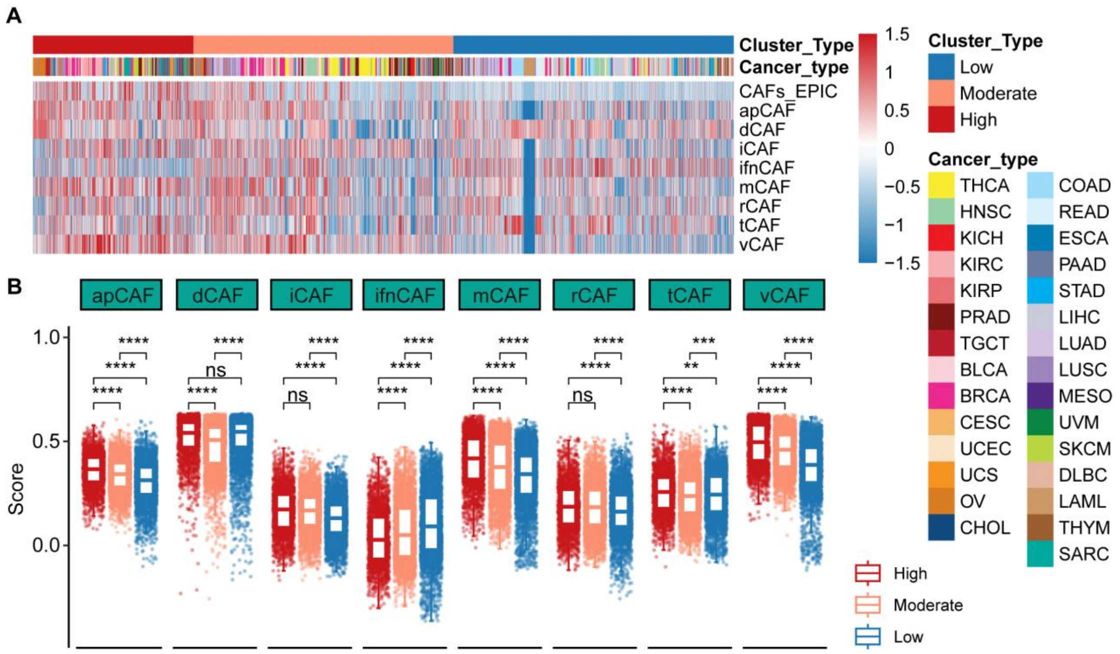

Figure S9. Differences of CAFs among neural signals-related molecular subtypes . (A) Heatmap showing the enrichment score of different CAF subtype signatures. (B) Boxplot displaying the distribution of different CAF signatures among molecular subtypes classified by neural signals. apCAF, antigen-presenting CAFs; dCAF, dividing CAFs; iCAF, inflammatory CAFs; ifnCAF, interferon response CAFs; mCAF, matrix CAFs;rCAF, reticular-like CAFs;tCAF, tumor-like CAFs;vCAF, vascular or vessel-associated CAFs.

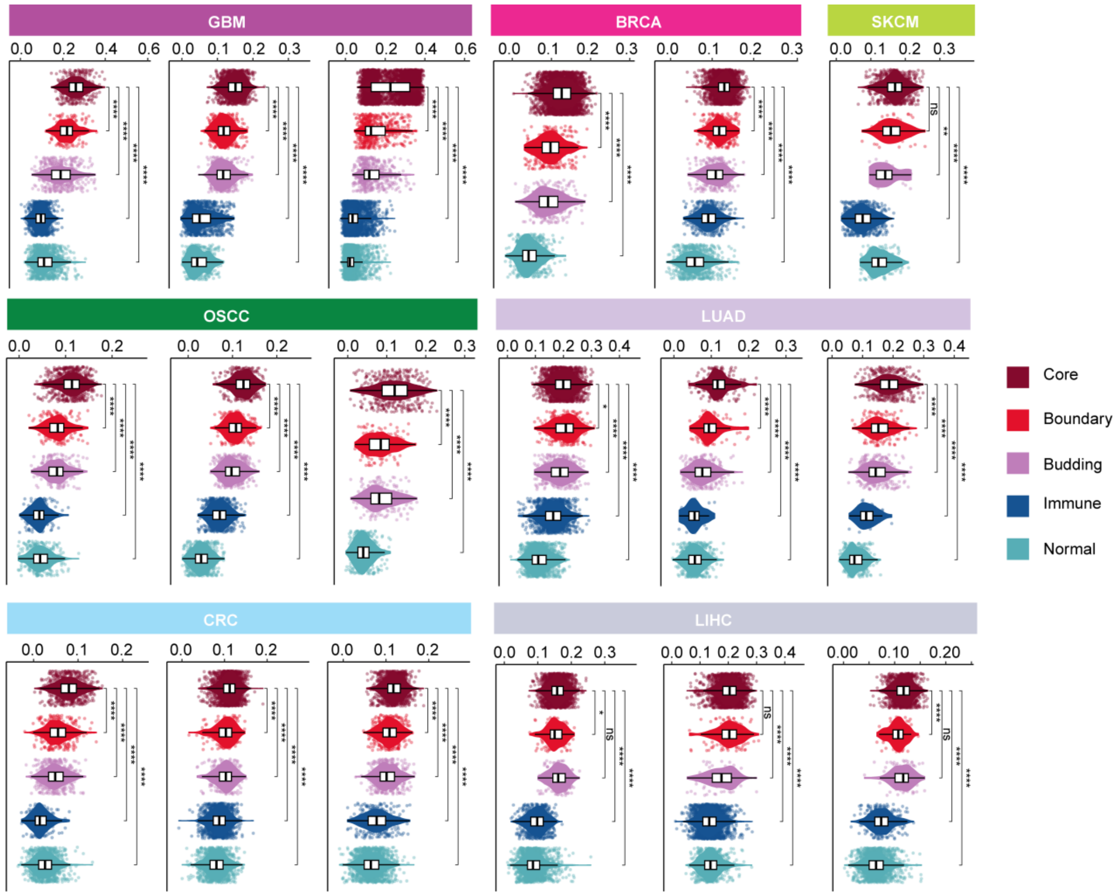

Figure S10. Neural signals activity in different regions based on the spatial transcriptome. VlnpIot exhibiting the distribution of neural signal activity across different regions in various cancer types, associated with Figure 4b. Wilcoxon rank sum test, P>0.5, ns; p<0.5, *; p<0.01, **; p<0.001, ***; p<0.0001, ****.

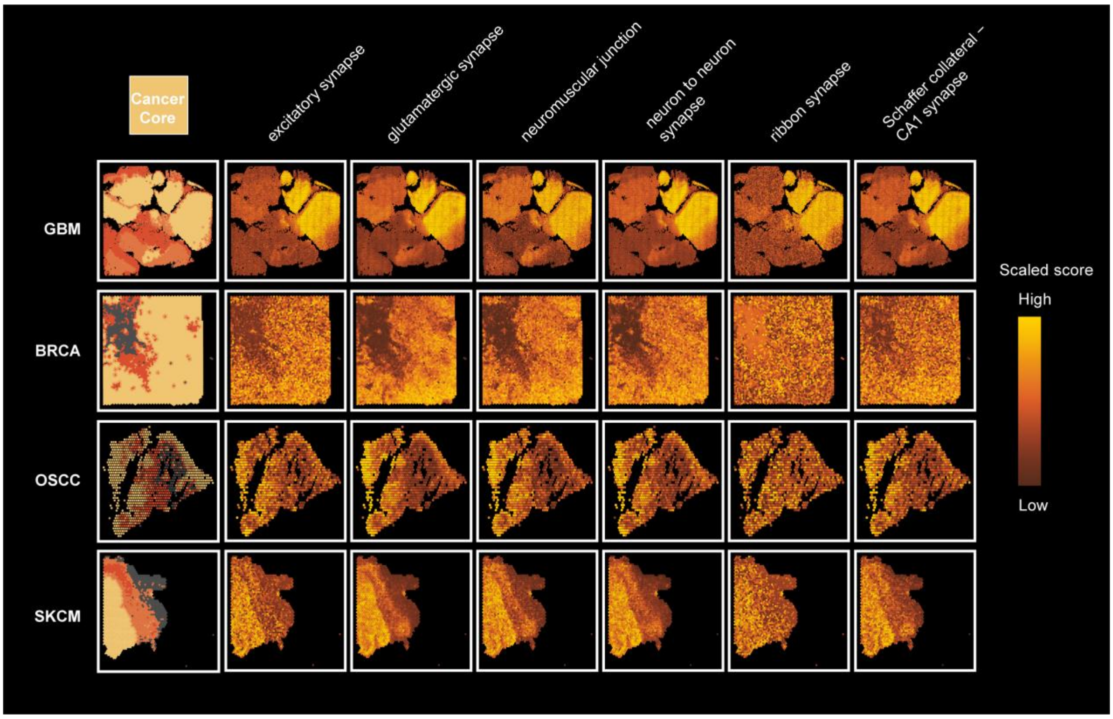

Figure S11. The activities of neural signals-related pathways in tumor core regions. The SpatialFeaturePlot displaying the activities of neural signals-related pathways in tumor core regions of GBM, BRCA, OSCC and SKCM.

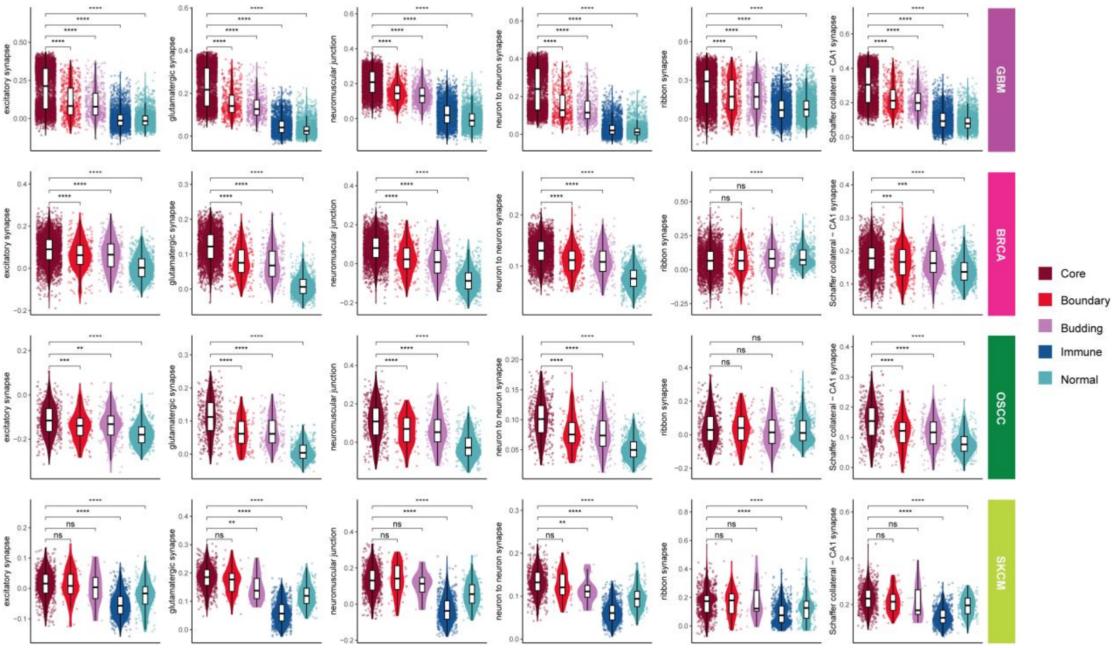

Figure S12. The activities of neural signals-related pathways in different regions. Vlnplot comparing the activities of neural signals-related pathways across different regions of various cancer types, associated with Figure S9. Wilcoxon rank sum test, P>0.5, ns; p<0.5, *; p<0.01, **; p<0.001, ***; p<0.0001, ****.

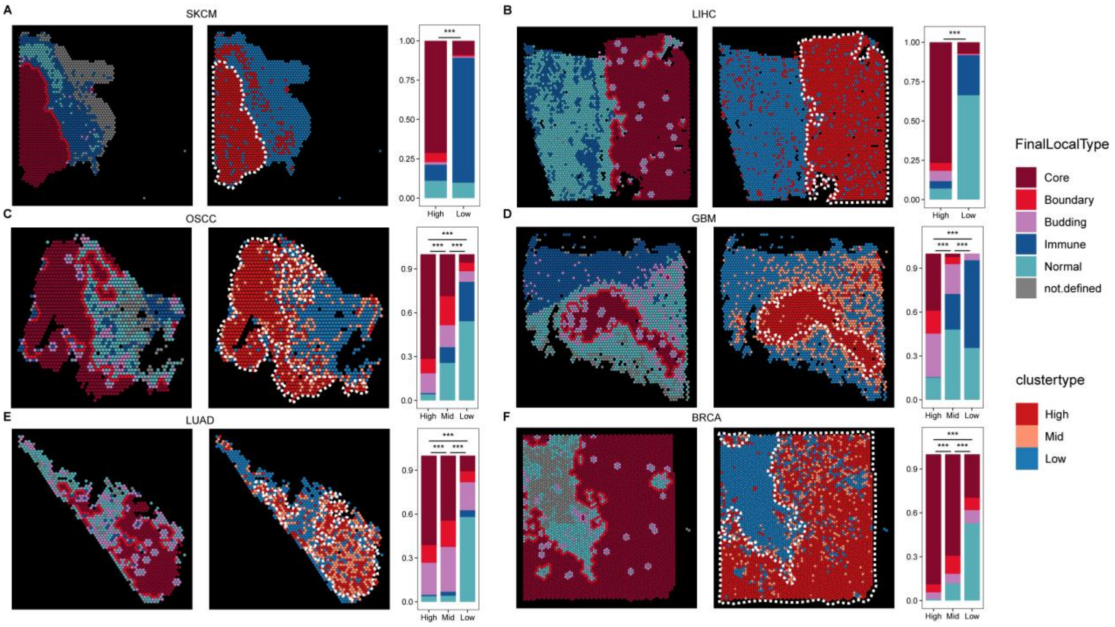

Figure S13. The association between neural signals-related molecular subtypes and diverse regions. The SpatialFeaturePlot comparing the distribution of neural signals-related molecular subtypes (middle) with diverse regions (left) of SKCM (A), LIHC (B), OSCC (C), GBM (D), LUAD (E) and BRCA (F). The bar plots (right) showing the proportion of spots from different regions in different subtypes.

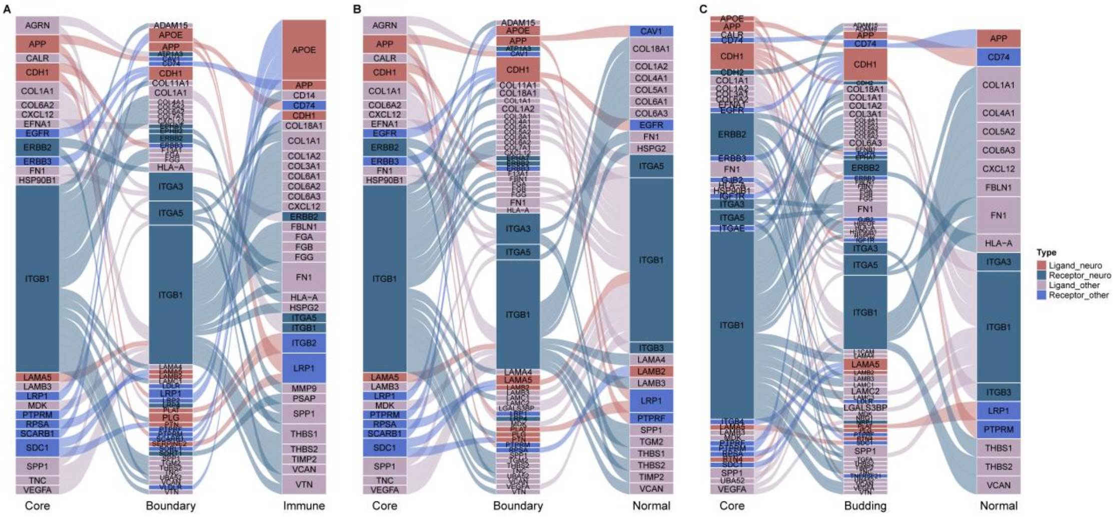

Figure S14. Ligand-receptor interactions between different regions. The sankey plots displaying the interaction of neural signals-related ligands or receptors and their paired receptors/ligands between malignant regions and non-malignant regions. Deep red represents neural-related ligands, and deep blue represents neural-related receptors.

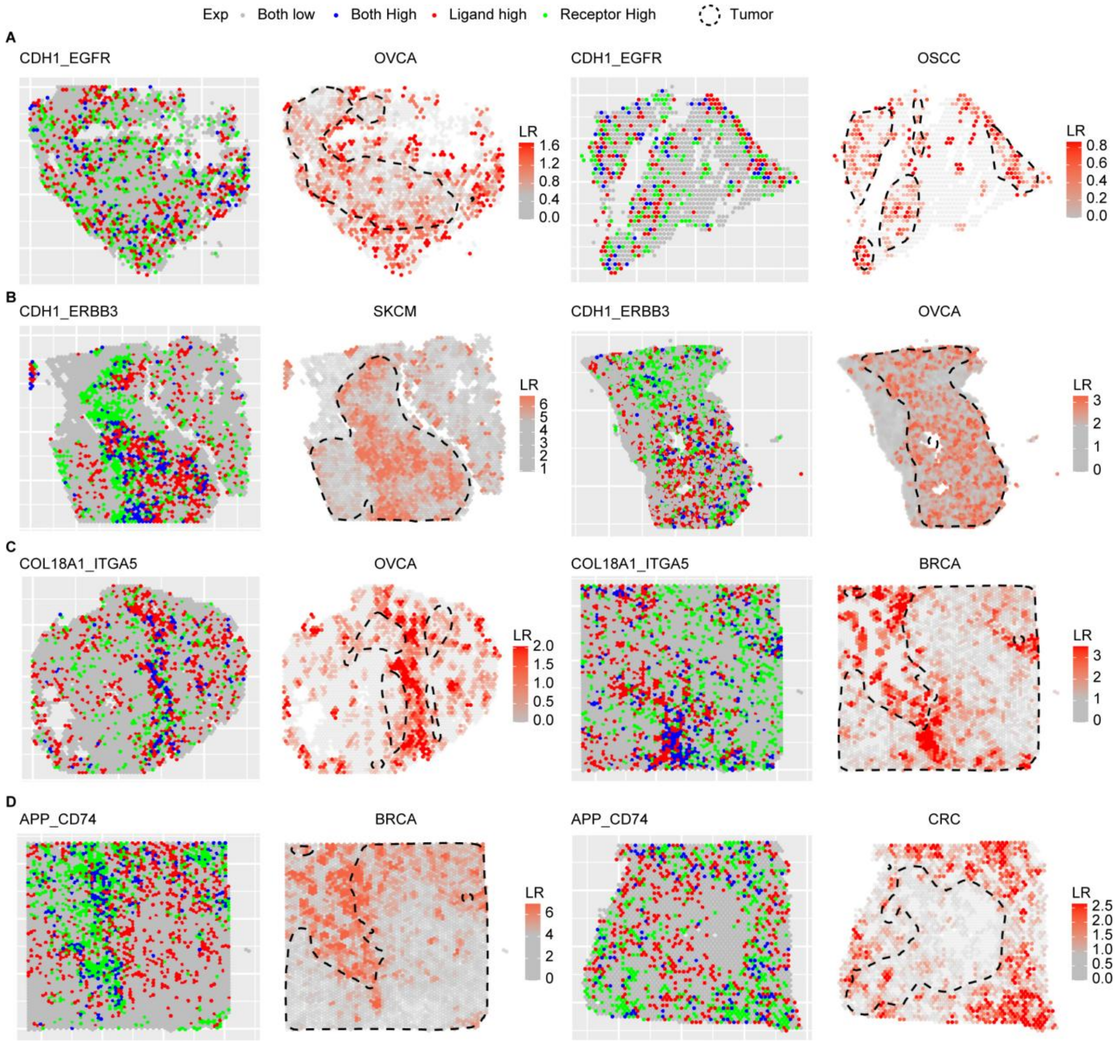

Figure S15. Spatial localization of Ligand-Receptor pairs. The scatter diagram displaying the expression distribution of the Ligand-Receptor pairs, such as *CDH1-EGFR* (A), *CDH1-ERBB3* (B), *COL18A1-ITGA5* (C) and *APP-CD74* (D).

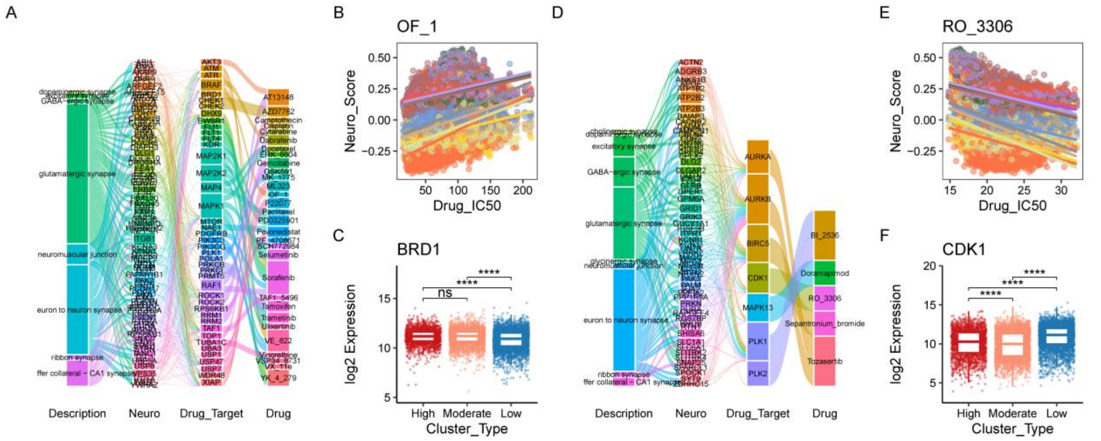

Figure S16. The correlation between neural signals and drug targets. (A) Sankey diagram illustrating the interaction between neural signals and the targets of positively correlated drugs. (B) The scatter plot showing shows the positive correlation between neural signals and IC50 values of OF-1. (C) Box plot displaying the expression distribution of OF-1 target *BRD1* in diverse neural subtypes. (D) Sankey diagram illustrating the interaction between neural signals and the targets of negatively correlated drugs. (E) The scatter plot showing shows the negative correlation between neural signals and IC50 values of RO-3306. (F) Box plot displaying the expression distribution of RO-3306 target *CDK1* in diverse neural subtypes.

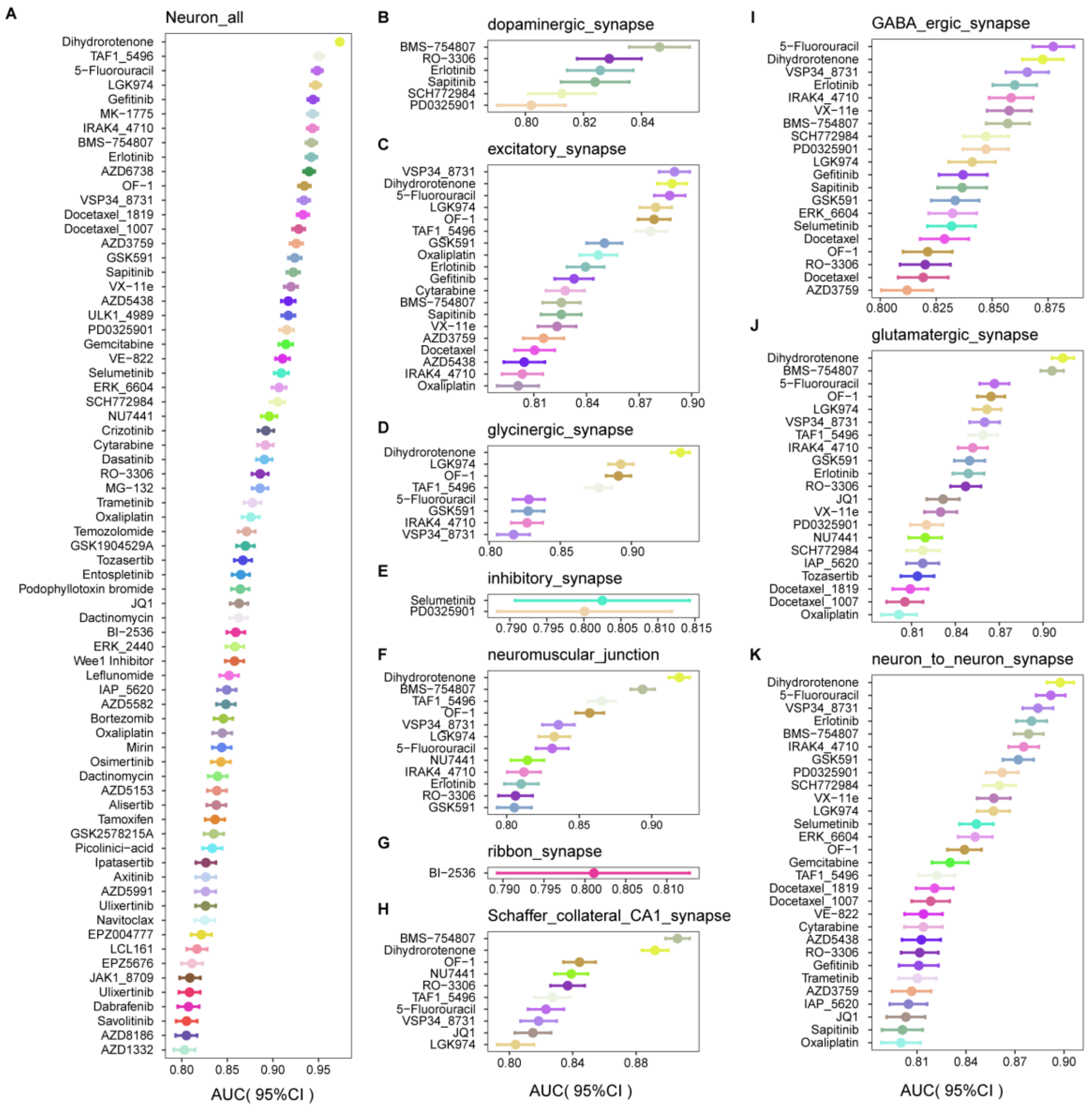

Figure S17. The efficacy of neural signals-related features in predicting drug sensitivity. The dot plots displaying the accuracy of neural signals-related features in predicting drug sensitivity via the area under the receiver operating characteristic curve (AUC). Only results with AUC greater than 0.8 were displayed.

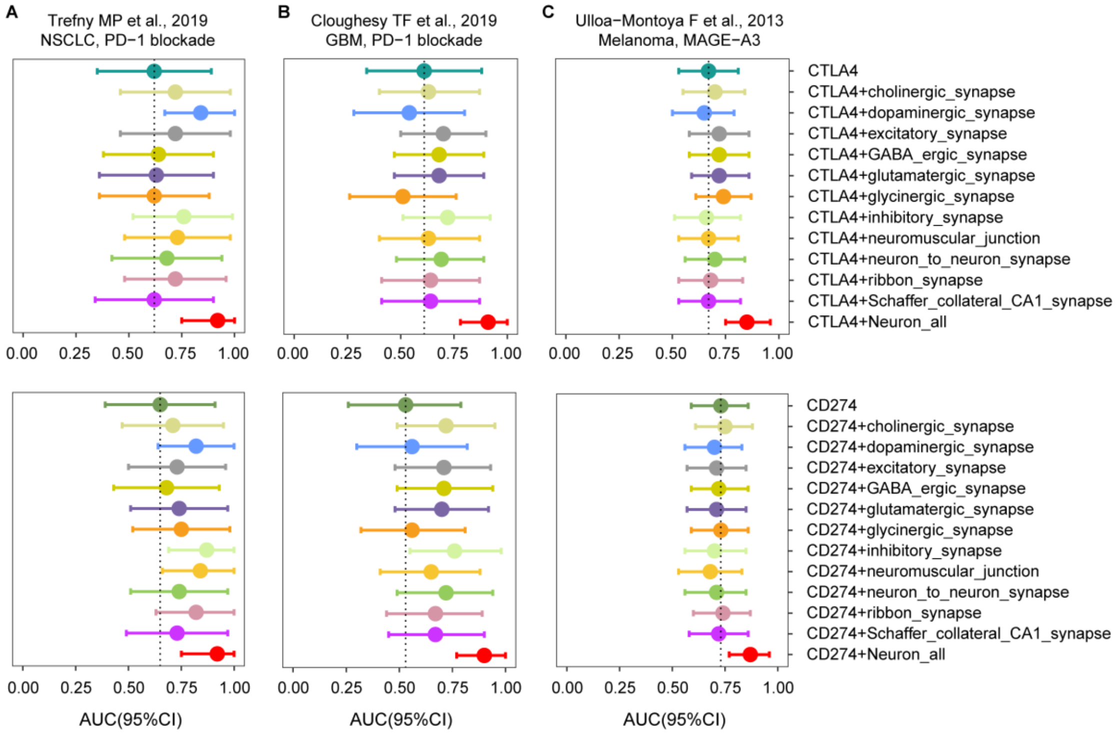

Figure S18. The efficacy of immune check points and combained neural signals-related features in predicting immunotherapy response. The dot plots displaying the accuracy of immune check points and combained neural signals-related features in predicting immunotherapy response via the area under the receiver operating characteristic curve (AUC), using NSCLC, GBM and Melanom cohorts.

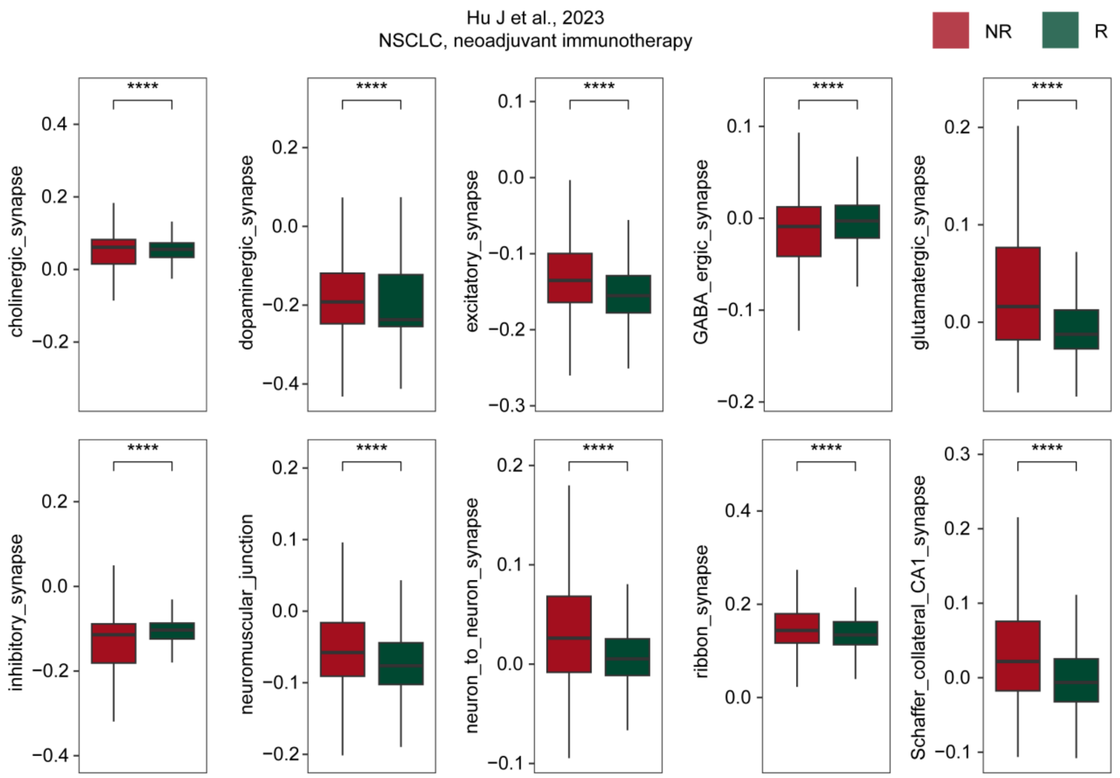

Figure S19. Activities of neural signals in responsive group and non-responsive group. The boxplot displaying the distribution of activities of neural signals in responsive group and non-responsive group using NSCLC single-cell cohort.

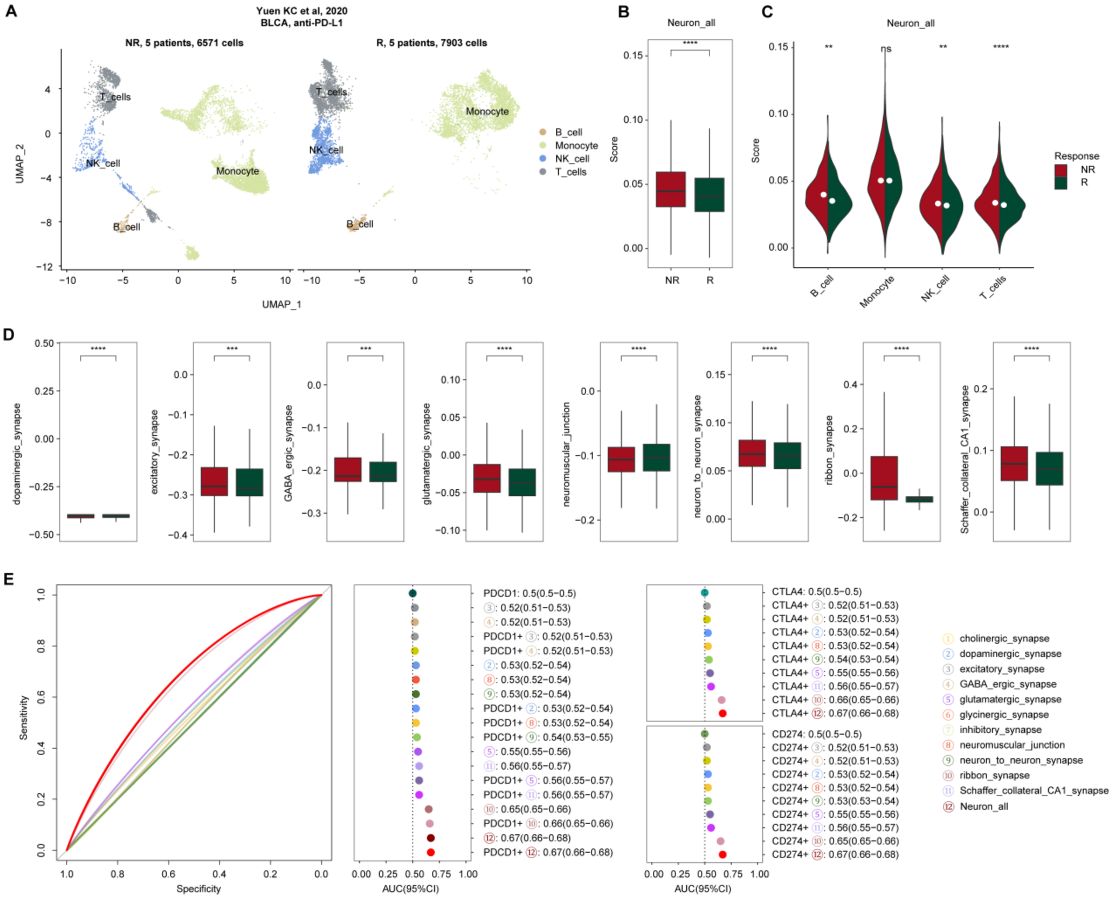

Figure S20. The performance of neural signals in predicting immune therapy response using BLCA single-cell cohort. (A) The umap showing cell types and number of cells in responsive group and non-responsive group. (B-D) The boxplot displaying he distribution of activities of neural signals in responsive group and non-responsive group, as well as in diverse cell types. (E) ROC curve (left) evaluating the ability of immune check points and combained neural signals-related features to predict immune therapy response. The dot plots (right) displaying the accuracy in predicting immunotherapy response via AUC value.

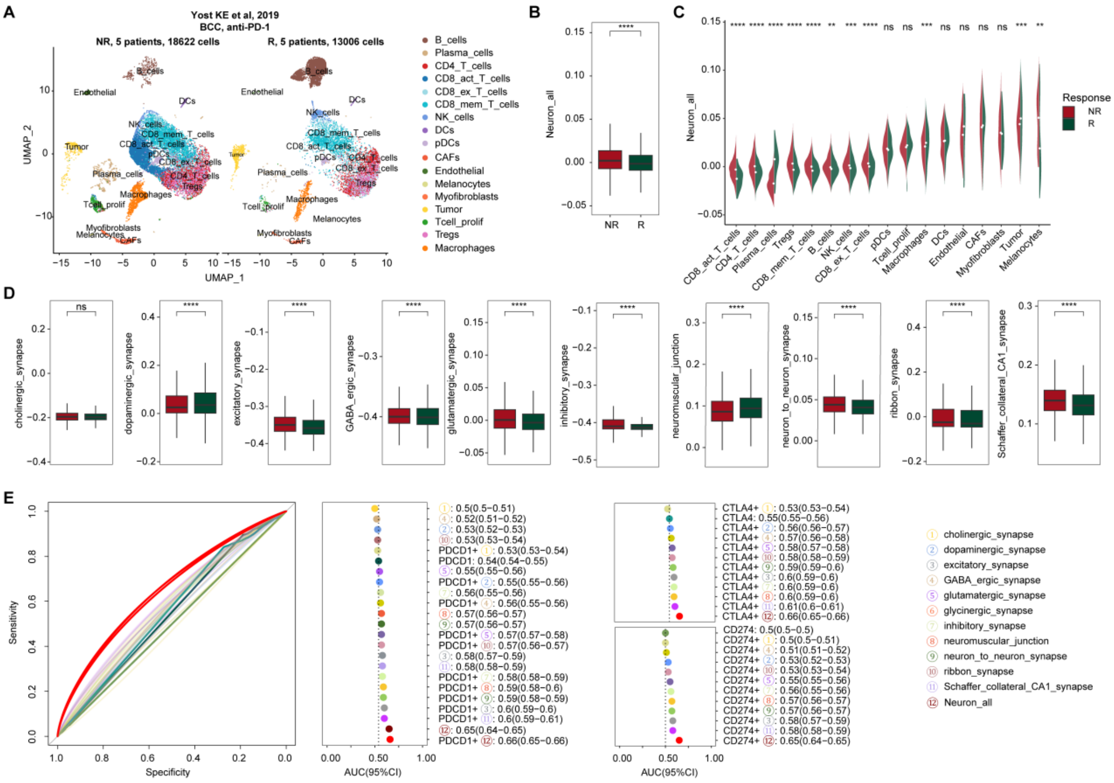

Figure S21. The performance of neural signals in predicting immune therapy response using BCC single-cell cohort. (A) The umap showing cell types and number of cells in responsive group and non-responsive group. (B-D) The boxplot displaying he distribution of activities of neural signals in responsive group and non-responsive group, as well as in diverse cell types. (E) ROC curve (left) evaluating the ability of immune check points and combained neural signals-related features to predict immune therapy response. The dot plots (right) displaying the accuracy in predicting immunotherapy response via AUC value.

Supplemental Tables

Table S1. Gene members of neural signal pathways.

Table S2. The genes involved in cancer or immune-related pathways.

Table S3. Correlations between activities of neural signals and abundances of TIME-related cell.

Table S4. The list of immune regulatory factors.

Table S5. DE results of immune-related factors between subtypes.

Table S6. AUC and 95%CI of neural signals-related signatures in predicting drug sensitivity.
