# Supplementary material for: Pan-cancer neural regulation pattern with implications for patient stratification and immunotherapy
Source: Mol Ther Nucleic Acids. 2025 Oct 7;36(4):102732. doi: 10.1016/j.omtn.2025.102732 (PMC12554035; doi:10.1016/j.omtn.2025.102732)
Supplement: Document S3. Article plus supplemental information [file mmc8.pdf]

Molecular Therapy
Nucleic Acids
Original Article

Pan-cancer neural regulation pattern with implications for patient stratification and immunotherapy

Yueying Gao,[1,2,5] Jiyu Guo,[1,2,5] Wenyi Yang,[3,5] Kefan Liu,[1] Qingyi Yang,[1] Si Li,[1] Dezhong Lv,[4] Yangyang Cai,[4] Kang Xu,[1] Weiwei Zhou,[4] Qinghua Jiang,[1] Juan Xu,[2,4] Yongsheng Li,[1,2] and Haoxiu Sun[1]

[1]School of Interdisciplinary Medicine and Engineering, Harbin Medical University, Harbin 150081, China; [2]State Key Laboratory of Frigid Zone Cardiovascular Diseases (SKLFZCD), Harbin Medical University, Harbin 150076, China; [3]Center for Bioinformatics, School of Life Science and Technology, Harbin Institute of Technology, Harbin 150081, China; [4]College of Bioinformatics Science and Technology, Harbin Medical University, Harbin 150081, China

The interplay between the nervous system and cancer plays a central role in regulating oncogenesis. We depict the pan-cancer landscape of neuro-tumor interactions by integration of >10,000 The Cancer Genome Atlas (TCGA) bulk and 239 pan-cancer spatial transcriptomics, in combination with 115 bulk and >120,000 single-cell checkpoint-inhibitor-treated transcriptomes. Neural signals are significantly correlated with various clinical features, and neural cancer subtypes have been characterized. High neural signals are associated with cancer-associated fibroblast infiltration and immune exclusion. Spatiotemporal transcriptome analysis reveals that tumor core regions present significantly higher neural signals and further unveils the pivotal modulators essential for tumor growth via ligand-receptor interaction networks. Neural signals are predictive biomarkers for drug treatment responses, particularly cancer immunotherapy. We constructed a comprehensive data resource Neuro_Cancer, to investigate neuro-tumor interactions in diverse cancer types. Our systematic dissection of the landscape of the neuronal regulation pattern provides a deeper understanding of tumorigenesis and potential therapeutic opportunities.

INTRODUCTION

The cross-talk between neural signals and tumor cells has been increasingly considered to play a pivotal role in regulating tumor proliferation, invasion, angiogenesis, immune exhaustion, and evasion.[1–5] Emerging studies have demonstrated the importance of nerve infiltration in tumor progression and adverse outcomes, as tumor cells also drive neuronal reprogramming to recruit new nerve fibers.[1,3,4,6] Neural-cancer interactions may reactivate neural-dependent biological processes to promote tumor progression.[1,4,7] Understanding the crosstalk between nerves and tumors is essential for advancing our understanding of the mechanisms of oncogenesis, malignant growth, and metastatic spread.

Many studies over the past decade have demonstrated the important roles of neural regulation in central nervous system (CNS) tumors. Neurons promote the growth of glioma mainly through the secretion of paracrine mitogens that drive proliferative signaling (e.g., the PI3K-mTOR, WNT, and β-catenin pathways).[2,4] Functional synaptic signaling from neurons to gliomas results in depolarizing currents in cancer cells, which promote tumor proliferation and invasiveness.[8] In addition, autocrine signaling activity is also induced to activate N-methyl-D-aspartate receptors (*NMDARs*) and their downstream effectors, thereby promoting invasiveness.[9] Previous studies have shown the role of glutamatergic and GABAergic signaling in driving the proliferation of cancer cells.[10] The γ-aminobutyric acid (GABA), the neurotransmitter of GABAergic synapses, primarily exerts inhibitory effects within the nervous system. It has revealed that GABA is able to sustain cancer cell proliferation and promote tumor progression by influencing angiogenesis and macrophage polarization.[11,12] It has been shown that excitatory neural signals, such as glutamatergic and cholinergic inputs, may promote tumor progression.[13] Glutamatergic synapses employ glutamate as their primary neurotransmitter, whereas cholinergic synapses utilize acetylcholine. These synapses primarily modulate neuronal excitability and facilitate tumor progression via the tumor-neural circuitry.[14–16] These findings emphasize the instrumental roles of neural signal activity from oncogenesis to malignant growth and metastatic spread in CNS tumors.

Moreover, numerous neural signals have direct or indirect connections with peripheral solid tumors, thereby affecting tumor pathogenesis and progression.[4,5,9,17] For example, breast cancer cells

Received 9 May 2025; accepted 2 October 2025;
https://doi.org/10.1016/j.omtn.2025.102732.

[5]These authors contributed equally

Correspondence: Juan Xu, College of Bioinformatics Science and Technology, Harbin Medical University, Harbin 150081, China.
E-mail: xujuanbiocc@ems.hrbmu.edu.cn
Correspondence: Yongsheng Li, School of Interdisciplinary Medicine and Engineering, Harbin Medical University, Harbin 150081, China.
E-mail: liyongsheng@ems.hrbmu.edu.cn
Correspondence: Haoxiu Sun, School of Interdisciplinary Medicine and Engineering, Harbin Medical University, Harbin 150081, China.
E-mail: sunhaoxiu@hrbmu.edu.cn

© 2025 The Author(s). Published by Elsevier Inc. on behalf of The American Society of Gene and Cell Therapy. This is an open access article under the CC BY license (http://creativecommons.org/licenses/by/4.0/).

promote the release of the neuropeptide substance P by inducing calcium signal activity in neurons, thus promoting growth and invasion of tumors.[18] Stress-induced neural activation increases primary tumor growth and tumor cell dissemination to the normal adjacent pancreas.[19] Crosstalk between neural signals and cancer cells has been observed in various cancer types, including breast cancer,[9] prostate cancer,[20] and pancreatic cancer.[19] Although the vital role of neural signals in cancer development and progression has been demonstrated in various types of tumor, the mechanistic details of such modulation remain to be discovered.

In this study, we integrated multiple omics data to evaluate the neural signal activity across various cancer types. In addition to CNS tumors, we revealed the intra- and intertumor heterogeneity of neural signals associated with peripheral solid tumors and further identified neural molecular subtypes. Higher levels of neural signal activity were associated with worse patient survival. In addition, the correlation between neural signal activity and the tumor immune microenvironment (TIME) was investigated via bulk and spatial transcriptomes (STs), and a significant correlation between neural signals and tumor- or immune-related signals was revealed. The performance of neural signals in predicting treatment response, particularly the immune therapy response, was further evaluated. Neural signals are potential predictive factors for immune therapy responses in various cancers. Our comprehensive analysis contributes to a deeper understanding of the mechanism underlying crosstalk between neural signals and tumors, offering valuable recommendations for future experimental studies and practical applications of cancer treatment.

RESULTS

Widespread neuronal activities across the CNS and peripheral solid tumors

To investigate the neural signal repertoire in cancer, we first evaluated the activities of neural pathways of more than 10,000 samples across 33 cancer types (Table S1). We found that neural pathway activities vary greatly not only between cancer types but also among different patients with the same type of tumor (Figure 1A). As expected, the neural signal activities of certain CNS tumors, such as brain lower grade glioma (LGG), pheochromocytoma and paraganglioma (PCPG), glioblastoma multiforme (GBM), and adrenocortical carcinoma (ACC), were generally greater than those of peripheral solid tumors (Figure 1A). Interestingly, numerous patients with peripheral solid tumors presented neural pathway activities comparable to those of CNS tumor patients (Figure 1A). In addition, neural-signal-related genes were more highly expressed globally in both the CNS and certain peripheral solid tumors (Figure S1A). We also compared neural signal activity with the abundance of infiltrated neuronal cells in patients[21] and revealed a significant correlation in both the CNS and peripheral solid tumors ($R = 0.88$ and 0.66, p values $<2.2e-16$) (Figures 1B and 1C), as well as in individual cancer types (p values <0.05) (Figure S2). Additionally, the activities of 11 neural signaling pathways were compared with those of infiltrated neuronal cells separately, and we also observed significant correlations in both the CNS and peripheral solid tumors (p values <0.05) (Figures 1D and S1B).

We next evaluated the correlations between neural signaling pathway activity and other genetic and clinical features. We found that there were significant differences in neural pathway activity not only between normal and tumor samples (Figure 1A) but also among samples at different pathological stages (Figure S1C). In addition, neural signaling pathway activity was significantly associated with the fraction of genome alterations (FGAs), mutation count, tumor mutation burden (TMB), tumor purity, and hypoxia in several cancer types (Figure S1D). Our results also revealed significant positive associations with cancer-related pathways (e.g., PI3K-AKT, cell proliferation, and EMT) (Figure 1D; Table S2) but negative associations with apoptosis and the immune response (Figure 1D), in both the CNS and peripheral solid tumors. We obtained similar results in individual cancer types (Figure S2). Taken together, these observations suggest the extensive infiltration of neural signals in cancer and their significant correlation with the tumor microenvironment, as well as highly tumor heterogeneity of neural pathways in both the CNS and peripheral solid tumors.

Characterization of neural signals reveals cancer subtypes

To further investigate the heterogeneity of neural signaling pathways across peripheral solid tumors, we next performed hierarchical clustering on the basis of neural pathway activity. Three molecular subtypes with high, moderate, and low neural signal activities were identified (Figures 2A, 2B, and S3A). The t-distributed stochastic neighbor embedding (t-SNE) mapping also demonstrated consistency within the same subtypes and heterogeneity among different subtypes (Figures 2B and S3B). In addition, cancer patients from similar tissues tended to cluster together and were further classified into the same neural subtype (Figure S3B). We next estimated the relative abundances of neuron cells in patients and found that although peripheral solid tumors presented relatively lower abundances than CNS tumors did ($p < 0.001$) (Figure 2C), significantly more neuron cells infiltrated patients in the high group ($p < 0.001$) (Figure 2C). Furthermore, we deconvoluted astrocytes, endothelial cells, microglia, excitatory neurons, inhibitory neurons, and oligodendrocytes among cancer patients.[22,23] The relative abundances in CNS tumors were the highest, followed by patients with higher neural signal activity (Figure S3C).

The associations between neural subtypes and the intrinsic biology of different cancer types were also examined. We observed that several cancer types (e.g., COAD, READ, and UCS) were allocated, for the most part, to the high or low neuron subtypes (Figure 2D). In addition, the proportion of patients with microsatellite instability was relatively greater in the group with lower neural signal activity than in the group with higher neural signal activity (Figure S3D). Patients in the lower group presented higher FGAs, TMB, mutation counts, and hypoxia scores and were more likely to develop metastasis (Figures 2E, 2F, and S3E–S3K). To further

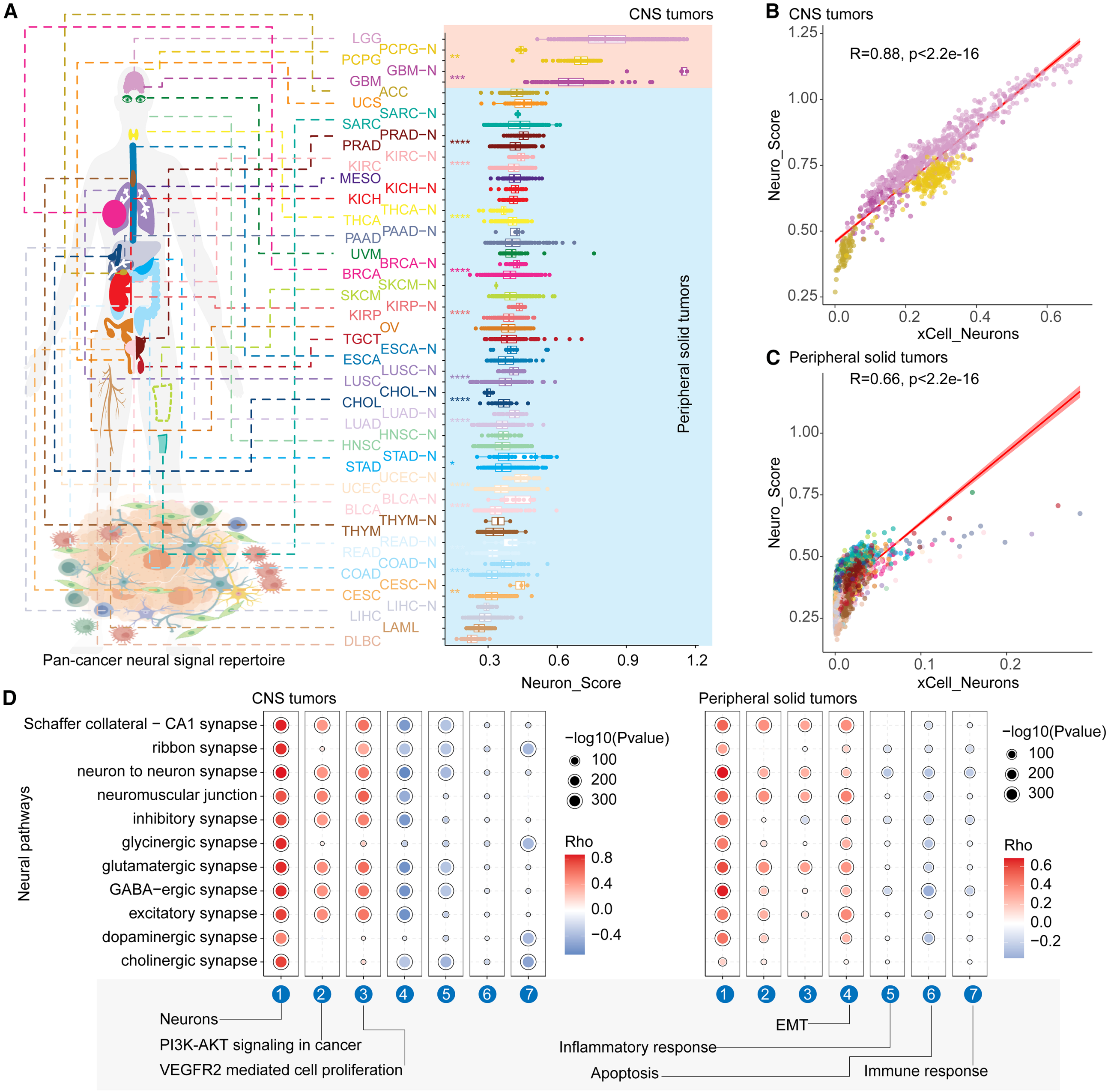

Figure 1. Widespread neuronal activities across the CNS and peripheral solid tumors

(A) Distribution of neural signal activity in various cancer types. (B and C) Scatterplot illustrating the correlation between neural signal activity and neuron abundance estimated by xCell in CNS tumors (B) and peripheral solid tumors (C). (D) Dot plot showing the correlation between the activities of diverse neural signaling pathways and hallmark signatures in CNS tumors and peripheral tumors.

validate the metastasis of patients with lower neural signaling, we collected expression profiles of primary tumors and metastatic tumors, including melanoma and colon cancer, and obtained similar results (Figure S4). Next, the Kaplan-Meier (KM) analysis revealed significant differences in the survival of patients among the three molecular subtypes, and patients in the high group presented poorer survival outcomes (log rank p values <0.0001) (Figures 2G–2J). Among different types of cancer, patients with higher neural signals show poorer prognosis in most cancers, especially in lung squamous cell carcinoma (LUSC), mesothelioma (MESO), bladder urothelial carcinoma (BLCA), and so on (log rank p values <0.05) (Figure S5). These findings revealed significant

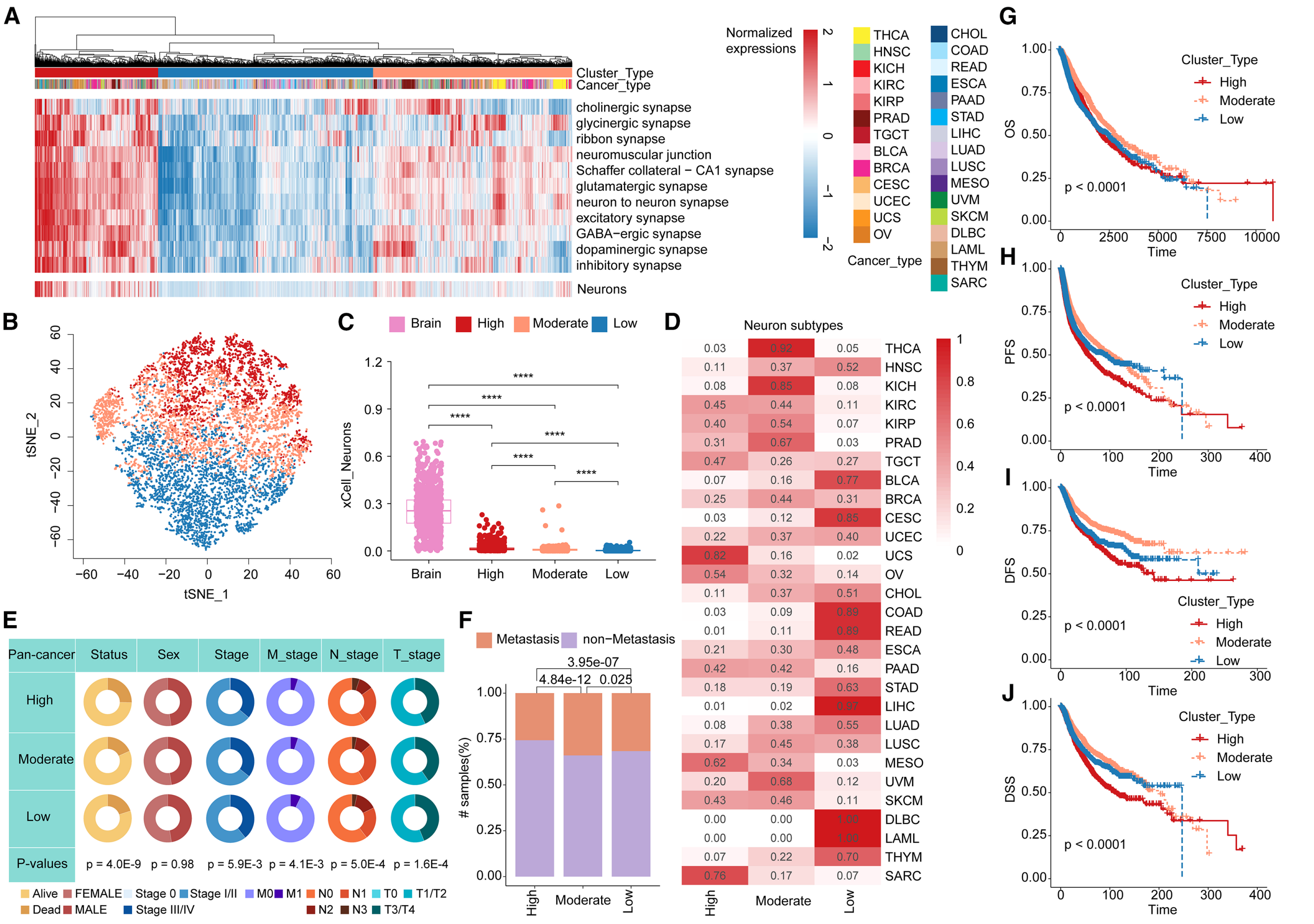

Figure 2. Characterization of neural signal reveals cancer subtypes

(A) Heatmap showing molecular subtypes classified by neural signal activity profiles. (B) Clustering results based on the t-SNE algorithm. (C) Boxplots displaying neuronal cell abundance across molecular subtypes. (D) Heatmap showing the proportions of diverse subtypes of each cancer. (E) Circular diagram showing the correlation between molecular subtypes and clinical features. (F) Bar plot showing the associations between molecular subtypes and metastasis. (G–J) Kaplan-Meier (KM) analysis was performed to evaluate prognostic value (OS, overall survival; PFS, progression-free survival; DFS, disease-free survival; DSS, disease-specific survival) of diverse molecular subtypes.

differences among the three molecular subtypes classified on the basis of the activities of neural signaling pathways, suggesting the potential clinical value of the neural subtyping of cancer.

Intricate tumor microenvironment of neural cancer subtypes

The tumor immune microenvironment (TME) plays a crucial role in the development and progression of cancer. To further investigate the functions of neural signaling pathways in tumor immunity, we next analyzed the associations between neural signals and immune- or cancer-related features. Significant differences in immune features were observed among diverse molecular subtypes (Figures 3 and S6). Compared with the neural-low group, patients with greater neural signal activity presented a lower abundance of immune cells and several myeloid cells such as $CD4^+$ T cells, $CD8^+$ T cells, and DC cells (Figures 3A, 3B, and S6A). Similar conclusions have also been drawn from analyses of public cohorts (Figure S6C). We also performed correlation analysis and observed a significant negative correlation between neural signals and these cell types (Figures S7A–S7C; Table S3). In particular, diverse macrophage subtypes exhibit distinct patterns of neural signaling activities. The abundance of M1 macrophages with pro-inflammatory function was significantly negatively correlated with neural signals, whereas the abundance of M2 macrophages with anti-inflammatory properties exhibited the opposite trend, revealing a more aggressive phenotype with greater neural signal activity (Figures 3B and S7C). We also found in the public cohorts that in most cancers, neural signals are negatively correlated with the abundance of M1 macrophages (Figure S6C). We further evaluated the association between signatures of macrophage subtypes and neural signals (Figure S8). The results showed that M1 macrophage features had higher expression in the neural-low group, while M2 macrophage features had higher expression in the neural-high group, especially the *CCR7* and *MMP19* (Figures S8A–S8C). The co-expression network suggests that by influencing the interplay of *CD86* and neural signal features such as *LYN*, *PLXNC1*, and

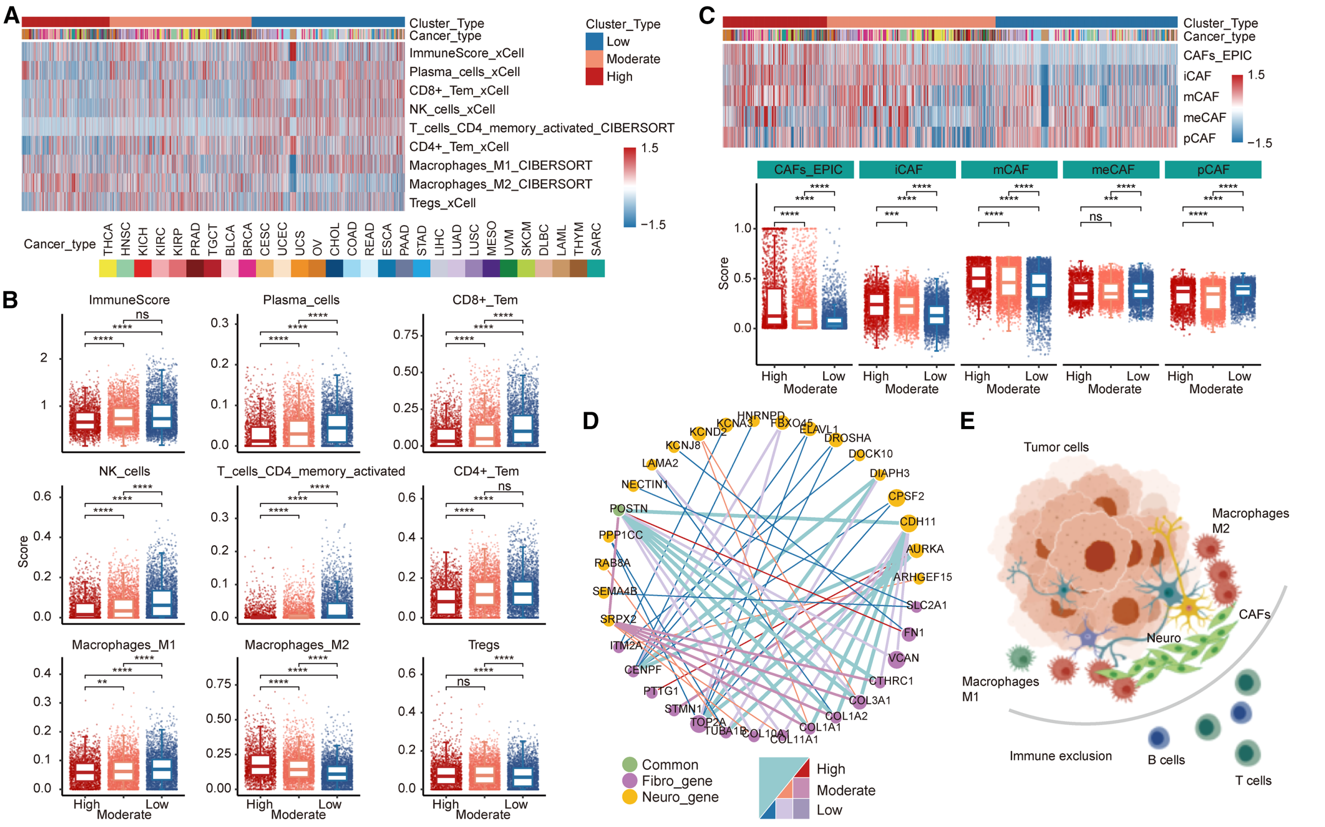

Figure 3. Intricate TMEs of neural cancer subtypes

(A) Heatmap showing the activity of immune features in cancer. (B) Boxplot showing the distribution of immune features across diverse molecular subtypes. (C) Associations between neural subtypes and CAF subtype signatures. Heatmap showing the enrichment score of CAFs in cancer samples. Boxplot display the distribution of CAF score. (D) Network displaying the interactions between neural-related genes and CAF-related genes. Purple represents CAF-related genes, and yellow represents neural-related genes. Green indicates the intersection of CAF-related genes and neural-related genes. (E) Schematic diagram of immune exclusion mediated by high neural signal activity. The neurons recruit fibroblasts to form a barrier tissue and promote immune cells exclusion.

DOCK10, it may mediate the interaction between neural signals and M1 macrophages, thereby affecting anti-tumor activity. The interaction between the M2 feature *FN1* and the neural signal feature *POSTN* may lead to the immunosuppressive phenotype in patients with higher neural signals (Figure S8D).

We also investigated the expression of immune regulatory factors among diverse subtypes (Tables S4 and S5). We found that immune checkpoint genes (e.g., *CTLA4*, *CD274*, and *PDCD1*) were highly expressed in the neural-low group, indicating that patients with lower neural signal activity were more likely to benefit from immune checkpoint inhibitor (ICI) therapy. Cancer-related genes, such as *CD70*, *VEGFA*, and *ENTPD1* (also known as *CD39*), which are reportedly associated with immune suppression, tumor proliferation, immune escape, and drug resistance,[24,25] exhibited increased expression in patients in the neural-high group (Figure S6B).

Notably, we observed a significant association between neural signals and cancer-associated fibroblasts (CAFs) (Figure S6A). The abundance of CAFs was highest in the neural-high group and exhibited a significant positive correlation with multiple neural signaling pathways (Figures S6A, S6C, S7A, and S7B). These results suggest that the activation of neural signals might recruit CAFs to synergistically promote cancer progression and immune escape. To further investigate the associations between CAF heterogeneity and neural signals, we next analyzed marker genes of CAF subtypes (iCAFs, inflammatory CAFs; mCAFs, matrix CAFs; meCAFs, metabolic CAFs; pCAFs, proliferative CAFs).[26] iCAFs are used to describe CAFs with pro-inflammatory effects, mainly characterized by high expression of cytokines and chemokines, which are associated with immune-related functions such as chemokine production and inflammatory response, as well as cell proliferation and migration. mCAFs are associated with extracellular matrix and angiogenesis and mainly play a pro-tumor role in TME. meCAF with a highly activated metabolic state is associated with hypoxia and typical glycolysis and may promote high metastatic potential of tumors. pCAFs exhibit high expression of cell-cycle-related genes, which are associated with cell proliferation.[26–29] We found that both iCAFs and mCAFs were more

abundant in the neural-high group (Figure 3C), which plays important roles in tumor angiogenesis and epithelial-mesenchymal transition (EMT), suggesting an immunosuppressive microenvironment in this group. An analysis of a more refined classification of CAF subtypes revealed similar trends (Figure S9). Higher levels of vascular or vessel-associated CAFs (vCAFs) and mCAFs were observed in the neural-high group (Figure S9), suggesting that higher neural signal activity may promote tumor angiogenesis and form a barrier around the tumor to block immune cell infiltration.

To further understand the potential mechanism between neural signals and CAFs, we next constructed a neural-CAF gene interaction network on the basis of co-expression analysis (adjusted $p < 0.05$ and Spearman correlation coefficient >0.7) (Figure 3D). The network revealed a significant positive correlation between genes related to neural signals and markers of CAFs. For example, *POSTN*, which plays an influential role in both neural signals and CAFs, is coexpressed with neural-signal-related genes (*CDH11* and *SRPX2*) and numerous CAF markers encoding extracellular matrix proteins (*COL1A1*, *COL1A2*, *COL3A1*, and *FN1*) (Figure 3D). In addition, several important neural-signal-related genes were identified, such as cadherin *CDH11* and the mitotic kinases *AURKA* and *SRPX2* (Figure 3D), which are significantly correlated with several CAF markers. Together, these findings elucidated the association between neural signals and the TME; in particular, the significant associations between neural signals and CAFs may play important roles in cancer progression and immune evasion (Figure 3E).[30]

Spatial patterns of neural signals across cancer types

The expansion and production of distinct neurons are crucial for the architectural assembly and formation of TMEs in cancer. To further investigate the role of neural signals in cancer, we collected the publicly available spatial transcriptomics (ST) data from 239 slices of 22 cancer types. On the basis of copy number variation (CNV) estimated by CopyKAT,[31] the spots in each slice were divided into five main groups: normal, immune, budding (tumor spots invading normal areas), boundary (between the core area of the tumor and other nonmalignant regions) and core (the core area of the tumor). We next investigated neural signal activity in spots of the ST and observed significantly increased neural signal activity in malignant regions, particularly in the tumor core area (Figure 4A). The activities of neural signals decreased from the core to immune regions (Figure 4A, bottom panel). For example, the spots in the core tumor area presented the strongest neural signals in GBM, BRCA, SKCM, OSCC, LUAD, CRC, and LIHC (Figures 4B and S10). The activities of numerous neural signaling pathways (e.g., excitatory synapses, glutamatergic synapses, and ribbon synapses) exhibited similar trends (Figures S11 and S12). Hierarchical clustering was used to divide the spots of each slice into diverse groups: neural-high, neural-mediate, and neural-low (Figure S13). We discovered that almost all spots belonging to malignant areas were classified into the neural-high group, whereas relatively more spots were classified into nonmalignant areas in the neural-low group (Fisher's test, p value <0.001) (Figure S13).

Cell-cell interactions, which are mediated by cell-type-specific ligand-receptor interactions in the TME, play crucial roles in impacting tumor growth. We next identified the significantly upregulated neural-signal-related receptors and ligands in different regions (Figure 4C). Amyloid precursor protein (*APP*), which reported to play an important role in intracellular signaling, synaptic and neuronal plasticity, and cell adhesion, was found to be significantly upregulated in malignant regions (Figure 4C), indicating its association with the invasion of tumor cells.[32] Further analysis of the neural-signal-related ligand-receptor networks suggested that *APP* may synergistically promote cancer proliferation and migration by interacting with *CD74*, *CAV1*, and *LRP1* in the tumor core region (Figure 4D), which were reported to enhance the migration and invasive capabilities of tumor cells and induce anti-PD-1 resistance.[33–35] We also identified other ligand-receptor interactions that are upregulated in malignant regions, such as the interaction between integrins (e.g., *ITGB1*, *ITGA5*, and *ITGA3*) and various extracellular matrix proteins (e.g., *COL4A1*, *FN1*, *MDK*, and *SPP1*) (Figure 4D), which may play important roles in angiogenesis to promote tumor progression.[36,37]

Moreover, to further investigate the potential mechanism by which cancer cells invade normal tissues, we constructed ligand-receptor interaction networks among diverse regions (Figure S14). The *APP-CD74* axis is located between the boundary region and immune region but is absent in the tumor and boundary regions (Figure S14A), indicating its associations with immune suppression in the TME.[38] The *APP-CD74* axis was also detected between the tumor core, budding, and normal regions (Figure S14C), suggesting its principal role in promoting the invasion of tumors into normal tissue. The interaction between *E-cadherin* and *EGFR* (the *CDH1-EGFR* axis) was observed between the tumor core, boundary, and normal regions (Figure S14B), which might lead to a significant increase in the proliferation of tumor cells via activation of the MEK/ERK signaling pathway.[39] Notably, the integrin and neural-signal-related receptors, such as *ITGB1*, *ITGA3*, and *ITGA5*, are bound to numerous ligands between tumor regions and normal regions (Figure S14), suggesting its critical impact on tumor invasion.[36,37] In addition, based on the SpaGene method, we evaluated the spatial coexpression between neural-signal-related ligands and receptors (Figure S15).[40] In terms of spatial localization, we also found that the neural-signal-related ligand *CDH1* interacts with *EGFR* mainly in the tumor surrounding area, which may promote tumor invasion into normal tissues (Figure S15A). The spatial coexpression of *CDH1* and *ERBB3* was only observed in the core region of the tumor, which may be related to the proliferation of tumor cells (Figure S15B). Interestingly, we found coexpression of *COL18A1* and the neural-signal-related receptor *ITGA5* in normal tissues near multiple tumor boundaries, rather than in normal tissues far from the tumor, which may further validate the role of *COL18A1-ITGA5* in promoting tumor cell migration and invasion in tumor-neural crosstalk (Figure S15C). Similar to previous results, the *APP-CD74* axis is enriched in nonmalignant areas around the tumor, which may induce immune suppression around the tumor and promote tumor cell

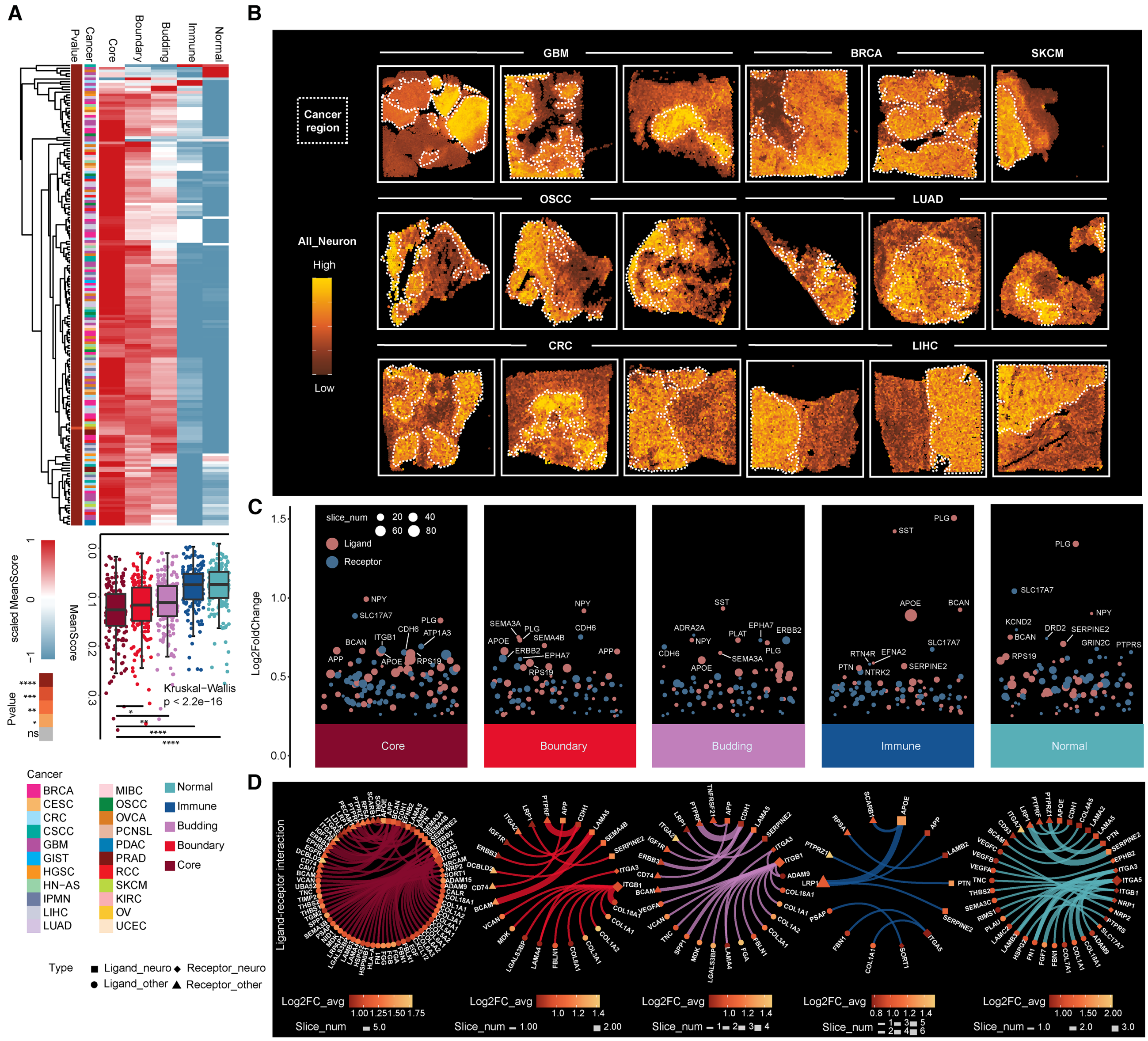

Figure 4. Spatial patterns of neural signals across cancers

(A) Heatmap and boxplot showing the distribution of neural signals activity across five regions: core (tumor core area); boundary (between the core area of the tumor and other normal or immune regions); budding (tumor spots invading normal areas); immune; and normal. (B) Spatial feature plot displaying the distribution of neural signal activity in tumor regions. (C) Dot plot showing the significantly upregulated neural-related ligand-receptor interactions in each region. (D) Networks showing the interactions between upregulated ligands and receptors in each region.

invasion (Figure S15D). Together, these results further demonstrate the spatial patterns of neural signals and reveal important ligand-receptor interactions that play critical roles in tumors.

Neural signatures are predictive biomarkers for drug response

Emerging evidence suggests that neural signals are closely related to the development of cancer treatment resistance and may serve as adjunctive strategies for chemotherapy and targeted therapy.[4,41] Therefore, we first used oncoPredict to predict the drug sensitivity value (IC50) of 198 available chemotherapy drugs and targeted drugs for cancer patients (Figure 5A).[42] We found that drugs exhibited different therapeutic sensitivities in diverse neural molecular subtypes (Figure 5A). In particular, we discovered that the DNA-dependent protein kinase inhibitor NU7441 had a greater IC50 in the group with low neural signal activity (Figure 5B), whereas the small molecule drug OF-1 had the opposite effect (Figure 5C). These observations suggest that patients with high neural signals might benefit from NU7441 treatment and be resistant to OF-1 treatment. In addition,

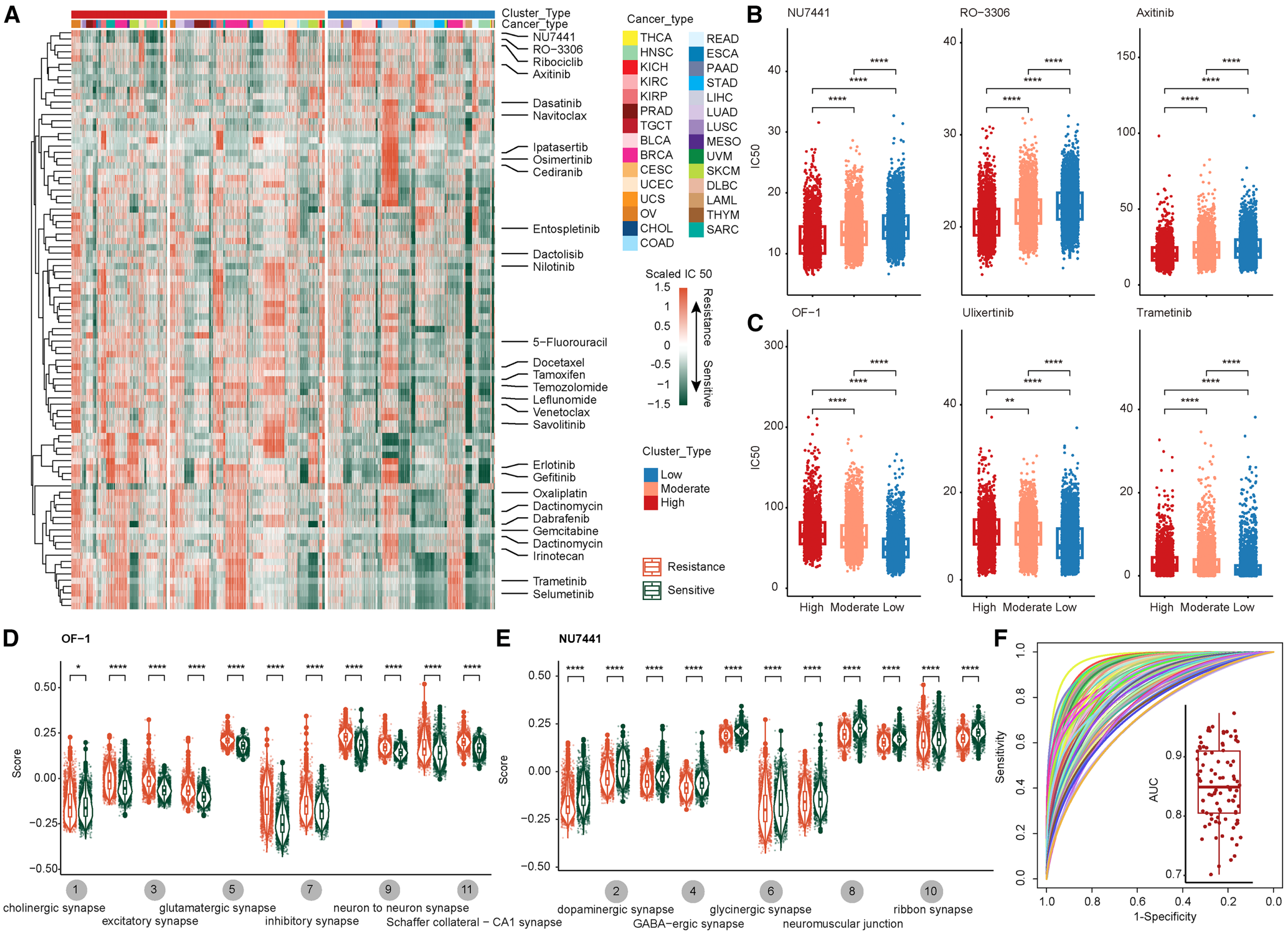

Figure 5. Neural signatures are predictive biomarkers for drug response

(A) Heatmap showing the drug sensitivity of cancer patients as predicted by oncoPredict. (B and C) Boxplot showing the distribution of drug IC50 values across diverse subtypes. (D and E) Boxplot displaying neural signal activity between drug-sensitive and the drug-resistant groups. (F) ROC curve for evaluating the ability of neural signatures to predict drug response.

we classified patients into sensitive and resistant groups on the basis of IC50 values of the drugs. We observed that patients were resistant to OF-1, whereas patients sensitive to NU7441 treatment presented significantly increased neural signaling pathway activity (Figures 5D and 5E). To further investigate the interaction between drug targets and neural signals, we evaluated their Spearman correlation and constructed the correlation networks between drug targets and neural signals (Figure S16). The results showed a significant negative correlation between OF-1 and neural signals, and the target *BRD1* of OF-1 showed higher expression in the neural-high group. Targeting the interaction between neural signals and *BRD1* may regulate resistance to OF-1 therapy (Figures S16A–S16C). On the contrary, RO-3306 is significantly negatively correlated with neural signals, and its target *CDK1* is expressed higher in the neural-low group (Figures S16D–S16F).

To further assess the ability of neural signal activity to evaluate drug sensitivity, we next constructed a logistic regression model based on neural-signal-related and combined gene expression features. The accuracy in predicting drug sensitivity was evaluated via the area under the receiver operating characteristic (ROC) curve (AUC). The models constructed on the basis of neural-signal-related features exhibited great prediction accuracy (Figure 5F), particularly neural-to-neural synapses, GABAergic synapses, and excitatory synapses, which can accurately predict the drug sensitivity of patients to common chemotherapy drugs and targeted drugs (Figure S17; Table S6). Moreover, models constructed on the basis of combined neural signal features performed better in predicting drug sensitivity (Figures 5F and S17A). These results suggest that the therapeutic drug response can be accurately predicted by neural signals, which may be valuable biomarkers for personalized treatment.

Neural-signal activity predicts cancer immunotherapy response

Our above results demonstrated a significant correlation between neural signals and the activities of drugs. Therefore, we

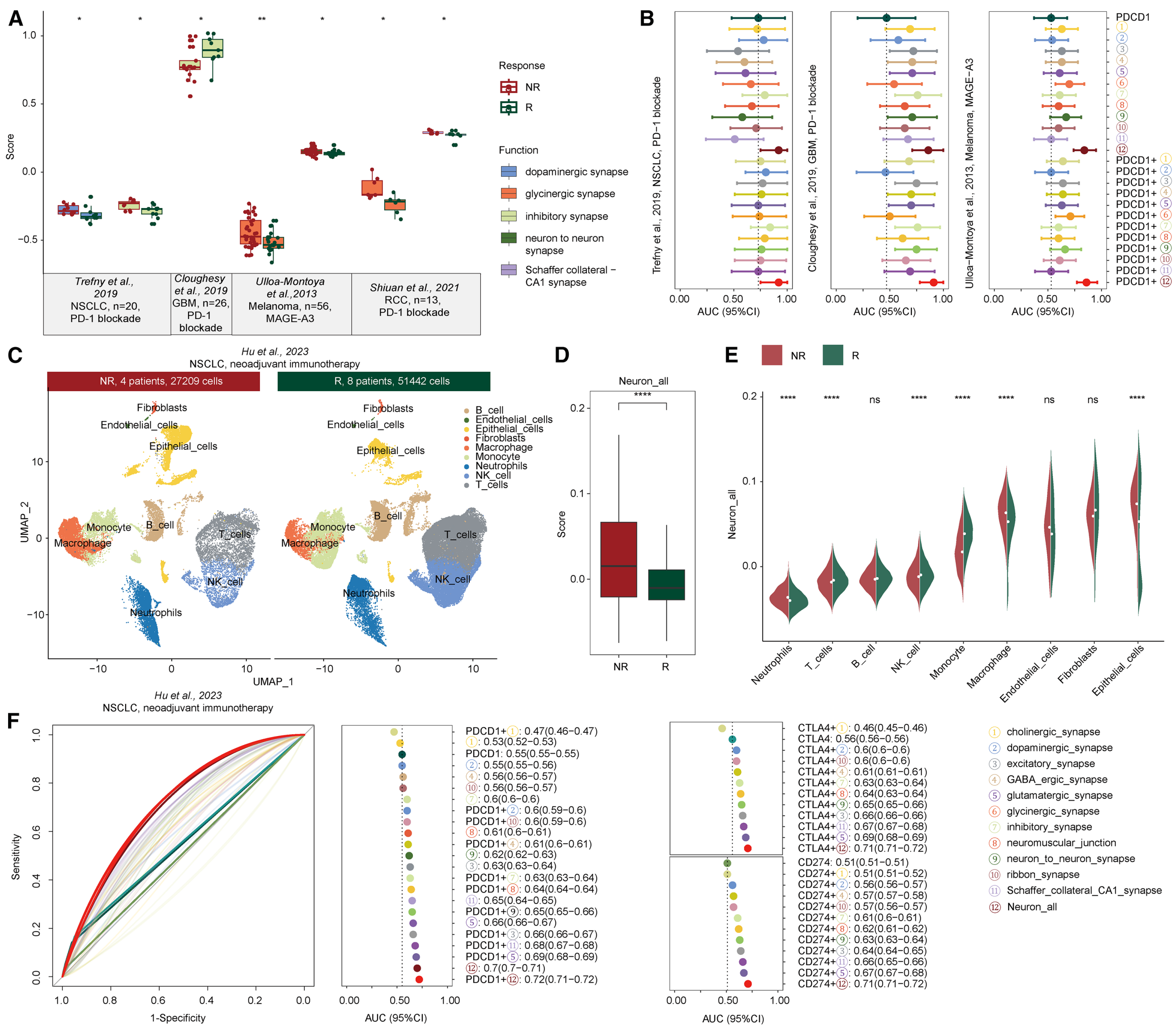

Figure 6. Neural signal activity predicts cancer immunotherapy response

(A) Boxplot showing the distribution of neural signal activity between the responsive group and nonresponsive group in external immunotherapy cohorts. (B) Dot plot showing the efficacy of combining neural features and immune checkpoint features in predicting the response to immune therapy. (C–E) Comparison of neural signal activity between the responsive and nonresponsive groups in the single-cell-level NSCLC cohorts. (F) ROC curve and dot plot showing the accuracy of combining neural-related features and immune checkpoint features in predicting immune therapy response in the NSCLC cohort.

hypothesized that neural signals might have the potential to predict immune therapy responses. Thus, several immunotherapy cohorts from the literature were collected,[43–46] to comprehensively evaluate the role of neural signal activity in immunotherapy. In the non-small cell lung cancer (NSCLC), melanoma, and renal cell carcinoma (RCC) cohorts, we observed significant differences in neural signal activity between immunotherapy responders and nonresponders, and the responders presented significantly lower neural signal activity (Figure 6A). The group with lower neural signals had higher TMB and immune signals, which may be more favorable for immunotherapy (Figure S3F). To further explore the important role of neural signals in predicting immune therapy responses, we next constructed logistic regression models based on three immune checkpoints—namely, *PDCD1* (*PD1*), *CD274* (*PDL1*), and *CTLA4*—as well as neural-signal-related features and their combinations. Compared with general immune checkpoint features, neural signal features, particularly the combined features, which serve as effective strategies for predicting immune therapy response, exhibited better predictive ability (Figures 6B and S18).

We further collected immune therapy cohorts at the single-cell level.[47–49] After quality control in the NSCLC cohort treated with neoadjuvant immunotherapy, a total of 78,651 cells were retained, of which 51,442 cells were in the treatment-responsive group and 27,209 cells were in the nonresponsive group (Figure 6C). We next compared the activities of neural signals of cells between responders and nonresponders, and the responsive group presented lower neural signal activity (Figures 6D and S19). We identified nine major cell types, including B cells, endothelial cells, epithelial cells, fibroblasts, macrophages, neutrophils, natural killer (NK) cells, and T cells (Figure 6C). Consistent with previous observations, there was a significant difference in neural signal activity between cancer-related cells and immune cells. Higher neural signal activity was observed in tumor-related cell types, such as epithelial cells, endothelial cells, and fibroblasts (Figure 6E), whereas lower neural signal activity was observed in immune cells (Figure 6E). In addition, the nonresponsive group presented increased neural signal activity in several types of cancer-related cells, whereas the opposite trend was observed in immune cells (Figure 6E). We also evaluated the ability of neural signal features to predict immune therapy responses and found superior predictive performance of neural signal features (Figure 6F). We next conducted the same analysis in the BLCA cohort treated with anti-PDL1 therapy and the BCC cohort treated with anti-PD1 therapy. Similar results were observed in these cohorts (Figures S20 and S21). These results from multiple immunotherapy cohorts have shown that increased neural signal activity is associated with increased malignancy and poorer treatment phenotypes and that neural signal represents effective predictor of immunotherapy response.

Neuro_Cancer: A comprehensive resource for exploring neural signals across cancers

We developed a comprehensive and interactive resource Neuro_Cancer (http://bio-bigdata.hrbmu.edu.cn/Neuro_Cancer/) to facilitate broad access to the neural signaling pathway activities in cancer (Figure 7). Users can query for neural signal activities, neuro-immune interactions, associations with clinical features, spatial patterns of neural signals, and drug and survival associations in a particular cancer context (Figure 7). All the data can be downloaded for further analysis. This valuable resource of neural signaling pathways in cancer will be of significant interest to the research community.

DISCUSSION

Previous studies have shown that tumors can affect nerves, which in turn may regulate tumor biology through direct or indirect pathways.[1,7] Although the crosstalk between neural signals and the TME has been investigated in CNS tumors, its role in peripheral solid tumors is still unclear. We elucidated the role of neural signaling pathways at the pan-cancer level, particularly regarding their potential impact on treatment strategies and immune therapy responses.

Pan-cancer analysis of neural pathway activity revealed high neural activity not only in CNS tumors but also in peripheral solid tumors. There was high intra- and intertumor heterogeneity. Hierarchical clustering was performed to classify patients into high, moderate, and low groups on the basis of the activities of neural signals. We revealed significant differences in clinical features, survival prognosis, biological function, and immune characteristics among diverse neural subtypes. Notably, the activity of neural signals is significantly positively correlated with EMT, which is a dynamic change in cells or tissues from the epithelial phenotype to the mesenchymal phenotype, leading to a more invasive state and increased resistance to apoptosis.[50] In addition, the neural-high subgroup was associated with worse patient survival. Elevated neural signal activity may signify a more aggressive tumor phenotype and contribute to the immunosuppressive tumor immune microenvironment, thereby increasing the likelihood of evasion from immune surveillance.

An increasing number of studies have shown that the nervous system interacts with the tumor microenvironment and plays an important role in the process of tumor development.[1,2,4] We explored the TME in cancer from the perspective of neural signals. The patient group with higher neural signal activity presented a complex tumor immune microenvironment (TIME) characterized by a reduced abundance of immune cells, increased abundance of tumor-related cells, and increased expression of immunosuppressive genes, indicating greater likelihood of immune evasion and a reduced response to immunotherapy. In particular, cancer-associated fibroblasts (CAFs) are significantly positively correlated with the activities of multiple neural signaling pathways. The synergistic effect of *POSTN* and other extracellular matrix proteins may lead to activated neural signals recruiting CAFs to synergistically promote cancer progression and immune evasion. In addition, the associations between subtypes of neural signals and CAFs revealed their crucial roles in tumor angiogenesis and epithelial mesenchymal transition, indicating an immunosuppressive microenvironment in the high-neural level group. In contrast, the neural-low group presented increased abundances of immune cells and pro-inflammatory M1 macrophages, indicating an immune infiltrating microenvironment that is more likely to benefit from immunotherapy. Further analysis of immunotherapy cohorts confirmed these findings. In contrast, the lower group demonstrated a potentially more favorable prospect for immunotherapy, characterized by a greater abundance of immune cells and significant upregulation of immune-related genes, which are typically associated with effective antitumor immune responses.

As innovative high-flux and high-dimensional technologies emerge, research on the spatial architecture of the TME has become feasible and reliable. On the basis of spatial transcriptomics data, we classified the spots into five regions—normal, immune, budding, boundary, and tumor core regions. In tumor regions, the activities of neural signals are increased, and interactions between integrins and various extracellular matrix proteins, which may play important roles in angiogenesis to promote tumor progression,[36,37] have been identified. Through further analysis of ligand-receptor interactions, we identified key regulators involved in tumorigenesis and development. Targeting these interactions may be an effective strategy for inhibiting tumor invasion ability.

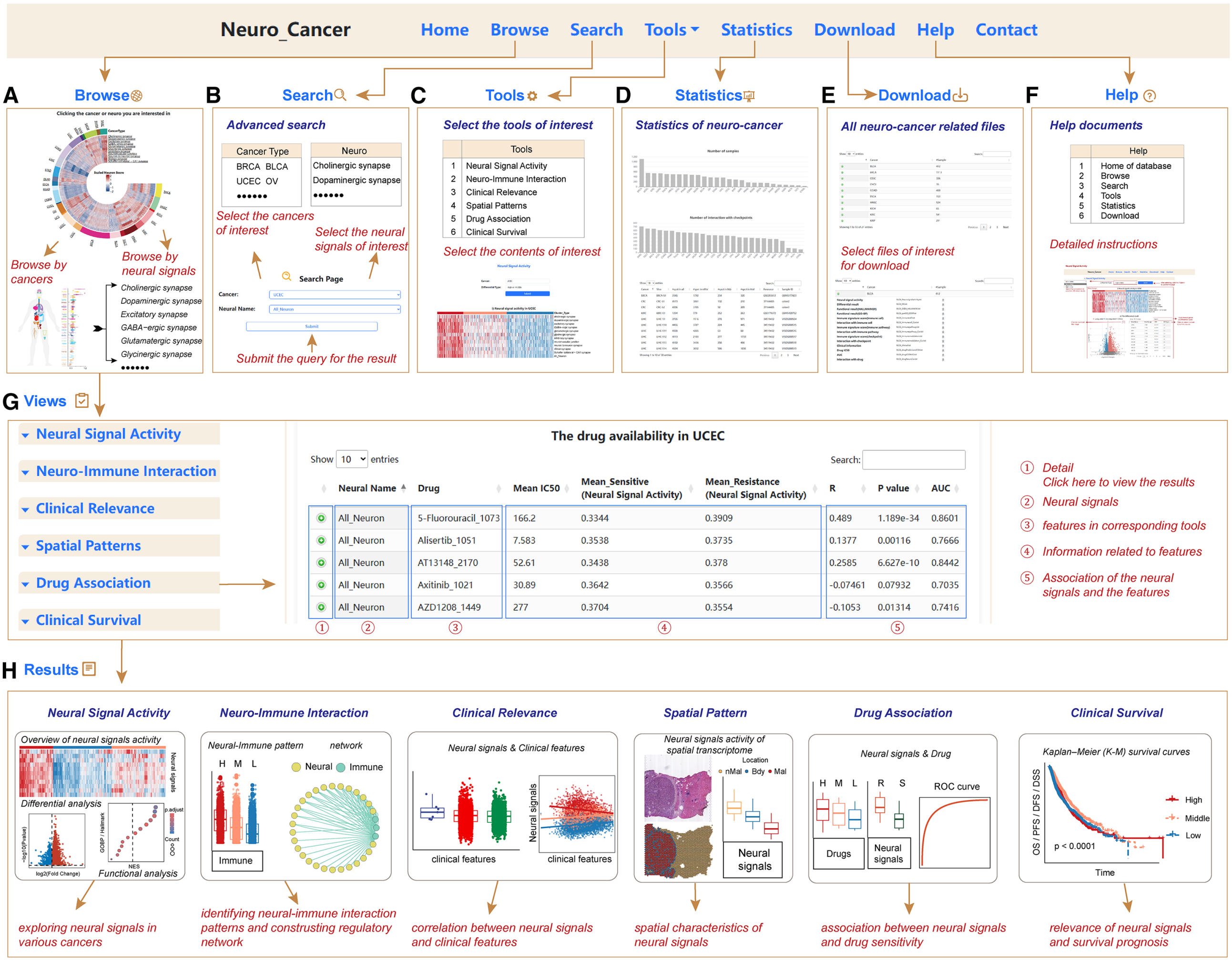

Figure 7. A comprehensive resource for exploring neural signals in cancer

Overview of the comprehensive resource—Neuro_Cancer. (A–F) Major functional modules of the web resource, including Browse (A), Search (B), Tools (C), Statistics (D), Download (E), and Help (F). (G) Detailed views of the results for drug associations. (H) Major results of the web resource, including neural signal activity, neuro-immune interaction, association with clinical features, spatial pattern of neural signals, drug association, and survival association.

It is crucial to accurately predict patients who will promptly respond to cancer treatments to tailor therapeutic strategies and minimize exposure to ineffective therapies. Therefore, we investigated the associations between the therapeutic response and neural signal activity of 198 available chemotherapy drugs and targeted drugs. We identified several drugs that are more sensitive in the neural-high group, such as the DNA-PK inhibitor NU7441, the CDK1 inhibitor RO-3306, and the vascular endothelial growth factor receptor (VEGFR) inhibitor axitinib, which might serve as complementary treatment strategies in the immunosuppressive microenvironment.[51–53] The models constructed on the basis of neural-signal-related features can also accurately predict the treatment sensitivity of drugs in cancer patients. We constructed prediction models on the basis of neural signal features in multiple cohorts and evaluated their ability to predict drug sensitivity and immune therapy response. The neural signal features exhibited better predictive performance in the bulk and single-cell RNA sequencing (scRNA-seq) cohorts than did the immune checkpoint genes *PD-1*, *PD-L1*, and *CTLA4*. Increasing evidence suggests that neural signals are associated with resistance to cancer therapy; these signals might be effective predictors of therapeutic response in cancer, and interventions in neural signaling pathways could represent a promising approach to overcoming treatment resistance. Given the modulation of the tumor microenvironment and immunity by neural signals, the integration of neuro-targeting strategies with other cancer treatment modalities, such as chemotherapy, targeted therapy, and immunotherapy, may yield improved therapeutic effects.

In summary, our research provides valuable insights into the molecular mechanisms and functional roles of neural signaling in cancer. Neural signals are effective factors for tumor progression and

potential guidelines for personalized clinical decision-making, particularly in immunotherapy strategies. Nevertheless, it is crucial to validate the role of neural signaling activity more extensively in other cancer cohorts. The dialogue between neural signals and tumors is not identical across various cancer types, necessitating further investigation, elucidation, and clinical utilization in human cancer medicine.

MATERIALS AND METHODS

Neural signal activity in cancer patients

We obtained a total of 743 neural-signal-related genes from the Gene Ontology (GO) resource (https://www.geneontology.org/), which was divided into 14 GO terms (Table S1). The "gsva" function of the gene set variation analysis (GSVA) (v.1.48.3) package was utilized,[54] with the parameters set as follows: (1) method = "ssgsea" and (2) min.sz = 5, to calculate the neural signal activity of each GO term in each sample. For scRNA-seq and ST datasets, GSVA was applied as described above to calculate the neural signal activity for every single cell or spot. Moreover, cancer-related pathways for evaluating neural activity are collected and stored in the supplementary table (Table S2).

Collection of transcriptome and clinical information

The gene expression profiles and clinical information of patients were derived from The Cancer Genome Atlas (TCGA; https://portal.gdc.cancer.gov/) and downloaded via the R package TCGAbiolinks (v.2.30.4), which includes 33 cancer types and 24 corresponding normal tissues. In addition, clinical information, including sex, tumor stage, fraction genome alterations (FGAs), mutation counts, tumor mutation burden (TMB), microsatellite instability, hypoxia score, and survival outcome, was also collected from cBioPortal (https://www.cbioportal.org/). More than 10,000 samples and more than 700 normal samples were included in total. The expression of genes with duplicate gene symbols was averaged.

Classification of neural molecular subtypes

On the basis of neural signal activity, we divided 9,427 cancer samples of peripheral solid tumors into three neural molecular subtypes via hierarchical clustering (high group, n = 2,159; moderate group, n = 3,497; low group, n = 3,771). To assess the reliability of the subtypes, we calculated the proportion of neurons in each sample via the xCell algorithm and examined the distribution of diverse neural molecular subtypes.[21] In addition, the deconvolution method based on nonnegative least squares (NNLS) was performed via the R package MIND (v.0.3.3).[23] The signature matrix utilized in this analysis, derived from Darmanis et al.,[22] contained six major cell types: astrocytes, endothelial cells, microglia, excitatory neurons, inhibitory neurons, and oligodendrocytes. We also examined their distribution in different groups.

Identification of neural signal interaction patterns

To identify the associations between neural signals and the TME in diverse subtypes, we collected immune- or cancer-related features from different sources. The relative abundance of various cells can be determined via diverse algorithms, such as the Microenvironment Cell Populations (MCP)-counter algorithm, the Estimate the Proportion of Immune and Cancer cells (EPIC) algorithm, the xCell algorithm, Immunophenoscore (IPS) algorithm, quanTIseq algorithm, and CIBERSORT algorithm.[21,55–59] The Wilcoxon rank-sum test was used to explore the activity patterns of immune features across diverse subtypes, and a p value <0.05 was considered statistically significant. We subsequently conducted Spearman correlation analysis to explore the associations between neural signals and immune features (Table S3). The significant correlations were visualized via the igraph (v.1.5.1) package. Furthermore, a list of immune regulatory factors, including 72 genes expressed in the transcriptome profile, was obtained from the literature to explore their expression patterns in diverse subtypes (Table S4). Wilcoxon rank-sum test was applied to evaluate the differences of immune-related features among diverse neural subtypes (Table S5).

Exploration of neural signals in spatial transcriptomes

The ST data were collected from public studies and consisted of 239 slices and 630,158 spots from 22 cancer types. On the basis of an integrative Bayesian segmentation approach called CopyKAT (https://github.com/navinlabcode/copykat),[31] we preliminarily divided the spots into a diploid normal group and an aneuploid tumor group on the basis of gene expression matrix of unique molecular identifier (UMI) counts. On the basis of the marker genes and the group of six spots around each spot, we further divided each slice into five regions: normal, immune, budding (tumor spot invading normal areas), boundary (between the core area of the tumor and other normal or immune regions), and core (core area of the tumor) regions. We applied the Single Sample Gene Set Enrichment Analysis (ssGSEA) algorithm to calculate the neural signal activity for each spot.[54] The SpatialFeaturePlot function in Seurat generated spatial feature expression plots. The Wilcoxon rank-sum test was used to compare the differences in neural signal activity in those regions. In addition, on the basis of prior ligand-receptor information, the FindMarkers function in Seurat was employed to identify crucial ligands or receptors that are differentially expressed between regions, and neural-signal-related L-R interaction networks within and between regions were constructed to explore the interaction patterns of neural signals.

Associations between drug sensitivity and neural signal activity

To predict the drug sensitivity of patients, we used the calcPhenotype function in the R package oncoPredict (v.1.2) to extrapolate the half maximal inhibitory concentration (IC50) value by building a ridge regression model.[42] The reference dataset used in this analysis originates from the Genomics of Drug Sensitivity in Cancer (GDSC; https://www.cancerrxgene.org/), the largest publicly available pharmacogenomic database.[60] Next, we explored the Spearman correlation between drug sensitivity and neural signal activity and investigated the distribution patterns of drug IC50 values among different subtypes via the Wilcoxon rank-sum test. In addition, to further elucidate the associations between drug

sensitivity and neural signals, we defined the 25% of samples with the lowest IC50 values as the sensitive group and the 25% of samples with the highest IC50 values as the resistant group. We subsequently explored the differences in neural signals between the two groups. To investigate whether neural signal activity was able to predict the response of diverse drugs (sensitivity or resistance) in cancer, we constructed logistic regression models with 5-fold cross-validation via the R package caret (v.6.0.94) on the basis of 11 neural signals and the combined features. Seventy percentage of the samples were used as training sets to train these models, and the predictive accuracy of these models was evaluated via ROC curves (Table S6).

Exploration of neural signal activities and immunotherapy

The transcriptome data and clinical information of the immunotherapy cohorts were obtained from the Gene Expression Omnibus (GEO, https://www.ncbi.nlm.nih.gov/geo/) and the literature (NSCLC, PD-1 blockade, GSE111414, $n = 20$; GBM, PD-1 blockade, GSE121810, $n = 26$; melanoma, MAGE-A3, GSE35640, $n = 56$; RCC, PD-1 blockade, Shiuan E et al.,[43] $n = 13$). The GSVA method was applied as described above to calculate the neural signal activity for each sample. We compared neural signal activity between the immunotherapy-responsive group and the unresponsive group via the Wilcoxon rank-sum test. To explore whether neural signal activity is able to predict the response to immunotherapy (responsive or unresponsive) in cancer, logistic regression models were constructed with 5-fold cross-validation via the R package caret (v.6.0.94), which is based on three immune checkpoints (*PD-1/PDCD1*, *PD-L1/CD274*, and *CTLA4*), 11 neural signals, and combined features. The accuracy of these models in predicting immunotherapy response was assessed via ROC curves.

Furthermore, immunotherapy cohorts at the single-cell level were also collected (NSCLC, neoadjuvant immunotherapy, GSE207422; BLCA, anti-PD-L1, GSE145281; BCC, anti-PD-1, GSE123813). Data processing and analysis, such as quality control, data normalization, dimensionality reduction, unsupervised clustering, and visualization, were carried out via the R package Seurat (v.4.3). The quality of the cells was assessed by the total UMI count per cell and the total number of genes detected per cell. Low-quality cells with <200 genes were filtered, and genes detected in fewer than three cells were also excluded from downstream analyses. After quality control, 78,651 cells from the NSCLC cohort, 14,474 cells from the BCC cohort, and 31,628 cells from the BLCA cohort were retained for subsequent analysis. Next, normalization was performed via the SCTransform function. We subsequently applied principal-component analysis (PCA) to reduce the dimensionality and used FindNeighbors and FindClusters functions to determine the optimal number of cell clusters, with the resolution set from 0.5 to 2, to select the most suitable resolution. For visualization of graph-based cell clustering, Uniform Manifold Approximation and Projection (UMAP) and t-Distributed Stochastic Neighbor Embedding (t-SNE) algorithms were applied with the RunUMAP and RunTSNE functions. On the basis of previously reported cell-type-specific markers, we further identified cell types and supplemented them with cell types annotated via the SingleR (v.1.0.1) R package. We compared the activity of neural signals across diverse cell types and between the immunotherapy-responsive group and the nonresponsive group. On the basis of the same features and methods mentioned above, logistic regression models were constructed, and their accuracy in distinguishing immune responses was evaluated via ROC curves.

Construction of Neuro_Cancer

The server-backend of Neuro_Cancer was built and accessed via Java Server Pages with the Tomcat container (v.6.0). The MySQL database (v.5.5.48) was utilized for Neuro_Cancer to document and manage all the metadata, and the web frontend was constituted with HTML, JavaScript, and CSS codes. All the analysis results and multiple statistical tables were visualized via the jQuery (v.3.3.1), DataTable (v.1.10.25), and ECharts (v.5.5.1) plugins. The R framework (v.4.3.1) was employed for the statistical analyses. Neuro_Cancer was tested on several popular web browsers, including the Google Chrome (preferred), Firefox, and Apple Safari browsers.

Survival analysis

KM analysis was performed via the survival and survminer R packages to evaluate the prognostic value (OS, overall survival; PFS, progression-free survival; DFS, disease-free survival; DSS, disease-specific survival) of diverse molecular subtypes. A log rank p value < 0.05 was considered statistically significant.

Statistical analysis

All data analysis was performed via R software (version 4.3.1). Comparisons between groups were performed via a two-tailed Wilcoxon rank-sum test if not specifically stated otherwise. For contingency table analysis, Fisher's exact test was utilized. Spearman correlation analyses were used to assess correlations between variables. Survival differences were evaluated via KM survival curves with log rank tests. A p value < 0.05 was considered statistically significant. The predictive performance of the models was quantified by the area under the AUC. The levels of significance are denoted as $^{*}p < 0.05$, $^{**}p < 0.01$, $^{***}p < 0.001$, and $^{****}p < 0.0001$.

DATA AND CODE AVAILABILITY

The transcriptome profiles that support the findings of this study are available in TCGA (https://portal.gdc.cancer.gov/). The public cohorts of gene expression profiles were collected from GEO (https://www.ncbi.nlm.nih.gov/geo/), with the identifiers GSE22219, GSE23036, GSE11969, GSE46517, GSE26193, GSE26712, GSE66957, GSE183795, GSE62165, GSE229904, and GSE62872. The spatial transcriptome data are deposited in GEO database, PubMed (https://pubmed.ncbi.nlm.nih.gov/), and 10x Genomics website (https://www.10xgenomics.com/), with the identifiers GSE203612, GSE179572, GSE208654, GSE217414, GSE206552, GSE144240, GSE195661, GSE194329, GSE185715, GSE181300, GSE227469, GSE233293, GSE189487, GSE171351, GSE208253, GSE203552, GSE211895, and GSE175540.[61–71] The immunotherapy cohorts are available from GEO database and literature without access restrictions under accession codes GSE111414, GSE121810, GSE35640,[43] GSE207422, GSE145281, and GSE123813. All software and algorithms used in this study are freely or commercially available and are listed in the materials and methods section. Any additional information required to reanalyze the data reported in this paper is available from the lead contact upon request.

ACKNOWLEDGMENTS

This work was supported by the National Science and Technology Major Project (2022ZD0117700), National Natural Science Foundation of China (T2325009, 32322020, 32170676, and 32060152); the Natural Science Foundation of Heilongjiang Province (Key Program) (ZD2023C007); and the project of scientific research business expenses of provincial research institutes (CZKYF2025-1-B044).

AUTHOR CONTRIBUTIONS

Conceptualization, H.S., Q.J., Y.L., and J.X.; methodology, Y.G., J.G., and W.Y.; software, K.L., J.G., and D.L.; formal analysis, Q.Y., S.L., Y.C., K.X., W.Z., and Y.G.; investigation, Y.G., J.G., and K.L.; data curation, all authors; writing—original draft, Y.G., J.G., J.X., and Y.L.; writing—review and editing, Y.G., K.L., Y.L., and J.X.; visualization: Y.G., J.G., K.L., and S.L.; supervision, Q.J., J.X., and Y.L.; project administration, Q.J., Y.L., and J.X.; funding acquisition, Q.J., Y.L., and J.X. All authors read and approved the final manuscript.

DECLARATION OF INTERESTS

The authors declare that they have no competing interests.

SUPPLEMENTAL INFORMATION

Supplemental information can be found online at https://doi.org/10.1016/j.omtn.2025.102732.

REFERENCES

1. Hanahan, D., and Monje, M. (2023). Cancer hallmarks intersect with neuroscience in the tumor microenvironment. Cancer Cell *41*, 573–580. https://doi.org/10.1016/j.ccell.2023.02.012.

2. Winkler, F., Venkatesh, H.S., Amit, M., Batchelor, T., Demir, I.E., Deneen, B., Gutmann, D.H., Hervey-Jumper, S., Kuner, T., Mabbott, D., et al. (2023). Cancer neuroscience: State of the field, emerging directions. Cell *186*, 1689–1707. https://doi.org/10.1016/j.cell.2023.02.002.

3. Wang, H., Zheng, Q., Lu, Z., Wang, L., Ding, L., Xia, L., Zhang, H., Wang, M., Chen, Y., and Li, G. (2021). Role of the nervous system in cancers: a review. Cell Death Discov. *7*, 76. https://doi.org/10.1038/s41420-021-00450-y.

4. Yaniv, D., Mattson, B., Talbot, S., Gleber-Netto, F.O., and Amit, M. (2024). Targeting the peripheral neural-tumour microenvironment for cancer therapy. Nat. Rev. Drug Discov. *23*, 780–796. https://doi.org/10.1038/s41573-024-01017-z.

5. Mancusi, R., and Monje, M. (2023). The neuroscience of cancer. Nature *618*, 467–479. https://doi.org/10.1038/s41586-023-05968-y.

6. Pan, C., and Winkler, F. (2022). Insights and opportunities at the crossroads of cancer and neuroscience. Nat. Cell Biol. *24*, 1454–1460. https://doi.org/10.1038/s41556-022-00978-w.

7. Zahalka, A.H., and Frenette, P.S. (2020). Nerves in cancer. Nat. Rev. Cancer *20*, 143–157. https://doi.org/10.1038/s41568-019-0237-2.

8. Venkatesh, H.S., Morishita, W., Geraghty, A.C., Silverbush, D., Gillespie, S.M., Arzt, M., Tam, L.T., Espenel, C., Ponnuswami, A., Ni, L., et al. (2019). Electrical and synaptic integration of glioma into neural circuits. Nature *573*, 539–545. https://doi.org/10.1038/s41586-019-1563-y.

9. Zeng, Q., Michael, I.P., Zhang, P., Saghafinia, S., Knott, G., Jiao, W., McCabe, B.D., Galván, J.A., Robinson, H.P.C., Zlobec, I., et al. (2019). Synaptic proximity enables NMDAR signalling to promote brain metastasis. Nature *573*, 526–531. https://doi.org/10.1038/s41586-019-1576-6.

10. Xiao, X., Huang, L., Li, M., and Zhang, Q. (2025). Intersection between lung cancer and neuroscience: Opportunities and challenges. Cancer Lett. *621*, 217701. https://doi.org/10.1016/j.canlet.2025.217701.

11. Barron, T., Yalçın, B., Su, M., Byun, Y.G., Gavish, A., Shamardani, K., Xu, H., Ni, L., Soni, N., Mehta, V., et al. (2025). GABAergic neuron-to-glioma synapses in diffuse midline gliomas. Nature *639*, 1060–1068. https://doi.org/10.1038/s41586-024-08579-3.

12. Huang, D., Alexander, P.B., Li, Q.J., and Wang, X.F. (2023). GABAergic signaling beyond synapses: an emerging target for cancer therapy. Trends Cell Biol. *33*, 403–412. https://doi.org/10.1016/j.tcb.2022.08.004.

13. Sun, Y., Wang, X., Zhang, D.Y., Zhang, Z., Bhattarai, J.P., Wang, Y., Park, K.H., Dong, W., Hung, Y.F., Yang, Q., et al. (2025). Brain-wide neuronal circuit connectome of human glioblastoma. Nature *641*, 222–231. https://doi.org/10.1038/s41586-025-08634-7.

14. Goethe, E.A., Deneen, B., Noebels, J., and Rao, G. (2023). The Role of Hyperexcitability in Gliomagenesis. Int. J. Mol. Sci. *24*, 749. https://doi.org/10.3390/ijms24010749.

15. Tetzlaff, S.K., Reyhan, E., Layer, N., Bengtson, C.P., Heuer, A., Schroers, J., Faymonville, A.J., Langeroudi, A.P., Drewa, N., Keifert, E., et al. (2025). Characterizing and targeting glioblastoma neuron-tumor networks with retrograde tracing. Cell *188*, 390–411.e36. https://doi.org/10.1016/j.cell.2024.11.002.

16. Venkataramani, V., Tanev, D.I., Strahle, C., Studier-Fischer, A., Fankhauser, L., Kessler, T., Körber, C., Kardorff, M., Ratliff, M., Xie, R., et al. (2019). Glutamatergic synaptic input to glioma cells drives brain tumour progression. Nature *573*, 532–538. https://doi.org/10.1038/s41586-019-1564-x.

17. Swanton, C., Bernard, E., Abbosh, C., André, F., Auwerx, J., Balmain, A., Bar-Sagi, D., Bernards, R., Bullman, S., DeGregori, J., et al. (2024). Embracing cancer complexity: Hallmarks of systemic disease. Cell *187*, 1589–1616. https://doi.org/10.1016/j.cell.2024.02.009.

18. Padmanaban, V., Keller, I., Seltzer, E.S., Ostendorf, B.N., Kerner, Z., and Tavazoie, S. F. (2024). Neuronal substance P drives metastasis through an extracellular RNA-TLR7 axis. Nature *633*, 207–215. https://doi.org/10.1038/s41586-024-07767-5.

19. Kim-Fuchs, C., Le, C.P., Pimentel, M.A., Shackleford, D., Ferrari, D., Angst, E., Hollande, F., and Sloan, E.K. (2014). Chronic stress accelerates pancreatic cancer growth and invasion: A critical role for beta-adrenergic signaling in the pancreatic microenvironment. Brain Behav. Immun. *40*, 40–47. https://doi.org/10.1016/j.bbi.2014.02.019.

20. Magnon, C., Hall, S.J., Lin, J., Xue, X., Gerber, L., Freedland, S.J., and Frenette, P.S. (2013). Autonomic nerve development contributes to prostate cancer progression. Science *341*, 1236361. https://doi.org/10.1126/science.1236361.

21. Aran, D., Hu, Z., and Butte, A.J. (2017). xCell: digitally portraying the tissue cellular heterogeneity landscape. Genome Biol. *18*, 220. https://doi.org/10.1186/s13059-017-1349-1.

22. Darmanis, S., Sloan, S.A., Zhang, Y., Enge, M., Caneda, C., Shuer, L.M., Hayden Gephart, M.G., Barres, B.A., and Quake, S.R. (2015). A survey of human brain transcriptome diversity at the single cell level. Proc. Natl. Acad. Sci. USA *112*, 7285–7290. https://doi.org/10.1073/pnas.1507125112.

23. Wang, J., Devlin, B., Roeder, K., and Kelso, J. (2020). Using multiple measurements of tissue to estimate subject- and cell-type-specific gene expression. Bioinformatics *36*, 782–788. https://doi.org/10.1093/bioinformatics/btz619.

24. Nilsson, M.B., Yang, Y., Heeke, S., Patel, S.A., Poteete, A., Udagawa, H., Elamin, Y. Y., Moran, C.A., Kashima, Y., Arumugam, T., et al. (2023). CD70 is a therapeutic target upregulated in EMT-associated EGFR tyrosine kinase inhibitor resistance. Cancer Cell *41*, 340–355.e6. https://doi.org/10.1016/j.ccell.2023.01.007.

25. Liu, L., Hou, Y., Deng, C., Tao, Z., Chen, Z., Hu, J., and Chen, K. (2022). Single cell sequencing reveals that CD39 inhibition mediates changes to the tumor microenvironment. Nat. Commun. *13*, 6740. https://doi.org/10.1038/s41467-022-34495-z.

26. Ma, C., Yang, C., Peng, A., Sun, T., Ji, X., Mi, J., Wei, L., Shen, S., and Feng, Q. (2023). Pan-cancer spatially resolved single-cell analysis reveals the crosstalk between cancer-associated fibroblasts and tumor microenvironment. Mol. Cancer *22*, 170. https://doi.org/10.1186/s12943-023-01876-x.

27. Cords, L., de Souza, N., and Bodenmiller, B. (2024). Classifying cancer-associated fibroblasts-The good, the bad, and the target. Cancer Cell *42*, 1480–1485. https://doi.org/10.1016/j.ccell.2024.08.011.

28. Chen, Z., Zhou, L., Liu, L., Hou, Y., Xiong, M., Yang, Y., Hu, J., and Chen, K. (2020). Single-cell RNA sequencing highlights the role of inflammatory cancer-associated fibroblasts in bladder urothelial carcinoma. Nat. Commun. *11*, 5077. https://doi.org/10.1038/s41467-020-18916-5.

29. Wang, Y., Liang, Y., Xu, H., Zhang, X., Mao, T., Cui, J., Yao, J., Wang, Y., Jiao, F., Xiao, X., et al. (2021). Single-cell analysis of pancreatic ductal adenocarcinoma identifies a novel fibroblast subtype associated with poor prognosis but better immunotherapy response. Cell Discov. *7*, 36. https://doi.org/10.1038/s41421-021-00271-4.

30. Jiang, S., Li, H., Zhang, L., Mu, W., Zhang, Y., Chen, T., Wu, J., Tang, H., Zheng, S., Liu, Y., et al. (2025). Generic Diagramming Platform (GDP): a comprehensive database of high-quality biomedical graphics. Nucleic Acids Res. *53*, D1670–D1676. https://doi.org/10.1093/nar/gkae973.

31. Gao, R., Bai, S., Henderson, Y.C., Lin, Y., Schalck, A., Yan, Y., Kumar, T., Hu, M., Sei, E., Davis, A., et al. (2021). Delineating copy number and clonal substructure in human tumors from single-cell transcriptomes. Nat. Biotechnol. *39*, 599–608. https://doi.org/10.1038/s41587-020-00795-2.

32. Lee, H.N., Jeong, M.S., and Jang, S.B. (2021). Molecular Characteristics of Amyloid Precursor Protein (APP) and Its Effects in Cancer. Int. J. Mol. Sci. *22*, 4999. https://doi.org/10.3390/ijms22094999.

33. Zhang, J.F., Tao, L.Y., Yang, M.W., Xu, D.P., Jiang, S.H., Fu, X.L., Liu, D.J., Huo, Y. M., Liu, W., Yang, J.Y., et al. (2021). CD74 promotes perineural invasion of cancer cells and mediates neuroplasticity via the AKT/EGR-1/GDNF axis in pancreatic ductal adenocarcinoma. Cancer Lett. *508*, 47–58. https://doi.org/10.1016/j.canlet.2021.03.016.

34. Lin, H., Fu, L., Zhou, X., Yu, A., Chen, Y., Liao, W., Shu, G., Zhang, L., Tan, L., Liang, H., et al. (2024). LRP1 induces anti-PD-1 resistance by modulating the DLL4-NOTCH2-CCL2 axis and redirecting M2-like macrophage polarisation in bladder cancer. Cancer Lett. *593*, 216807. https://doi.org/10.1016/j.canlet.2024.216807.

35. Wang, Y., Li, Y., Zhong, J., Li, M., Zhou, Y., Lin, Q., Zong, S., Luo, W., Wang, J., Wang, K., et al. (2023). Tumor-derived Cav-1 promotes pre-metastatic niche formation and lung metastasis in breast cancer. Theranostics *13*, 1684–1697. https://doi.org/10.7150/thno.79250.

36. Sun, L., Guo, S., Xie, Y., and Yao, Y. (2023). The characteristics and the multiple functions of integrin β1 in human cancers. J. Transl. Med. *21*, 787. https://doi.org/10.1186/s12967-023-04696-1.

37. Guo, J., Ma, X., Liu, D., Wang, F., Xia, J., Zhang, B., Zhao, P., Zhong, F., Chen, L., Long, Q., et al. (2024). A distinct subset of urothelial cells with enhanced EMT features promotes chemotherapy resistance and cancer recurrence by increasing COL4A1-ITGB1 mediated angiogenesis. Drug Resist. Updat. *76*, 101116. https://doi.org/10.1016/j.drup.2024.101116.

38. Chen, G., Wang, W., Wei, X., Chen, Y., Peng, L., Qu, R., Luo, Y., He, S., Liu, Y., Du, J., et al. (2024). Single-cell transcriptomic analysis reveals that the APP-CD74 axis promotes immunosuppression and progression of testicular tumors. J. Pathol. *264*, 250–269. https://doi.org/10.1002/path.6343.

39. Russo, G.C., Crawford, A.J., Clark, D., Cui, J., Carney, R., Karl, M.N., Su, B., Starich, B., Lih, T.S., Kamat, P., et al. (2024). E-cadherin interacts with EGFR resulting in hyper-activation of ERK in multiple models of breast cancer. Oncogene *43*, 1445–1462. https://doi.org/10.1038/s41388-024-03007-2.

40. Liu, Q., Hsu, C.Y., and Shyr, Y. (2022). Scalable and model-free detection of spatial patterns and colocalization. Genome Res. *32*, 1736–1745. https://doi.org/10.1101/gr.276851.122.

41. Liu, D., Li, X., Chen, X., Sun, Y., Tang, A., Li, Z., Zheng, J., and Shi, M. (2019). Neural regulation of drug resistance in cancer treatment. Biochim. Biophys. Acta. Rev. Cancer *1871*, 20–28. https://doi.org/10.1016/j.bbcan.2018.10.001.

42. Maeser, D., Gruener, R.F., and Huang, R.S. (2021). oncoPredict: an R package for predicting in vivo or cancer patient drug response and biomarkers from cell line screening data. Brief. Bioinform. *22*, bbab260. https://doi.org/10.1093/bib/bbab260.

43. Shiuan, E., Reddy, A., Dudzinski, S.O., Lim, A.R., Sugiura, A., Hongo, R., Young, K., Liu, X.D., Smith, C.C., O'Neal, J., et al. (2021). Clinical Features and Multiplatform Molecular Analysis Assist in Understanding Patient Response to Anti-PD-1/PD-L1 in Renal Cell Carcinoma. Cancers (Basel) *13*, 1475. https://doi.org/10.3390/cancers13061475.

44. Cloughesy, T.F., Mochizuki, A.Y., Orpilla, J.R., Hugo, W., Lee, A.H., Davidson, T.B., Wang, A.C., Ellingson, B.M., Rytlewski, J.A., Sanders, C.M., et al. (2019). Neoadjuvant anti-PD-1 immunotherapy promotes a survival benefit with intratumoral and systemic immune responses in recurrent glioblastoma. Nat. Med. *25*, 477–486. https://doi.org/10.1038/s41591-018-0337-7.

45. Ulloa-Montoya, F., Louahed, J., Dizier, B., Gruselle, O., Spiessens, B., Lehmann, F.F., Suciu, S., Kruit, W.H.J., Eggermont, A.M.M., Vansteenkiste, J., and Brichard, V.G. (2013). Predictive gene signature in MAGE-A3 antigen-specific cancer immunotherapy. J. Clin. Oncol. *31*, 2388–2395. https://doi.org/10.1200/jco.2012.44.3762.

46. Trefny, M.P., Rothschild, S.I., Uhlenbrock, F., Rieder, D., Kasenda, B., Stanczak, M. A., Berner, F., Kashyap, A.S., Kaiser, M., Herzig, P., et al. (2019). A Variant of a Killer Cell Immunoglobulin-like Receptor Is Associated with Resistance to PD-1 Blockade in Lung Cancer. Clin. Cancer Res. *25*, 3026–3034. https://doi.org/10.1158/1078-0432.Ccr-18-3041.

47. Yost, K.E., Satpathy, A.T., Wells, D.K., Qi, Y., Wang, C., Kageyama, R., McNamara, K.L., Granja, J.M., Sarin, K.Y., Brown, R.A., et al. (2019). Clonal replacement of tumor-specific T cells following PD-1 blockade. Nat. Med. *25*, 1251–1259. https://doi.org/10.1038/s41591-019-0522-3.

48. Yuen, K.C., Liu, L.-F., Gupta, V., Madireddi, S., Keerthivasan, S., Li, C., Rishipathak, D., Williams, P., Kadel, E.E., Koeppen, H., et al. (2020). High systemic and tumor-associated IL-8 correlates with reduced clinical benefit of PD-L1 blockade. Nat. Med. *26*, 693–698. https://doi.org/10.1038/s41591-020-0860-1.

49. Hu, J., Zhang, L., Xia, H., Yan, Y., Zhu, X., Sun, F., Sun, L., Li, S., Li, D., Wang, J., et al. (2023). Tumor microenvironment remodeling after neoadjuvant immunotherapy in non-small cell lung cancer revealed by single-cell RNA sequencing. Genome Med. *15*, 14. https://doi.org/10.1186/s13073-023-01164-9.

50. Yang, J., Antin, P., Berx, G., Blanpain, C., Brabletz, T., Bronner, M., Campbell, K., Cano, A., Casanova, J., Christofori, G., et al. (2020). Guidelines and definitions for research on epithelial–mesenchymal transition. Nat. Rev. Mol. Cell Biol. *21*, 341–352. https://doi.org/10.1038/s41580-020-0237-9.

51. Motzer, R.J., Penkov, K., Haanen, J., Rini, B., Albiges, L., Campbell, M.T., Venugopal, B., Kollmannsberger, C., Negrier, S., Uemura, M., et al. (2019). Avelumab plus Axitinib versus Sunitinib for Advanced Renal-Cell Carcinoma. N. Engl. J. Med. *380*, 1103–1115. https://doi.org/10.1056/NEJMoa1816047.

52. Tsai, A.K., Khan, A.Y., Worgo, C.E., Wang, L.L., Liang, Y., and Davila, E. (2017). A Multikinase and DNA-PK Inhibitor Combination Immunomodulates Melanomas, Suppresses Tumor Progression, and Enhances Immunotherapies. Cancer Immunol. Res. *5*, 790–803. https://doi.org/10.1158/2326-6066.Cir-17-0009.

53. Powles, T., Plimack, E.R., Soulières, D., Waddell, T., Stus, V., Gafanov, R., Nosov, D., Pouliot, F., Melichar, B., Vynnychenko, I., et al. (2020). Pembrolizumab plus axitinib versus sunitinib monotherapy as first-line treatment of advanced renal cell carcinoma (KEYNOTE-426): extended follow-up from a randomised, open-label, phase 3 trial. Lancet Oncol. *21*, 1563–1573. https://doi.org/10.1016/s1470-2045(20)30436-8.

54. Hänzelmann, S., Castelo, R., and Guinney, J. (2013). GSVA: gene set variation analysis for microarray and RNA-seq data. BMC Bioinf. *14*, 7. https://doi.org/10.1186/1471-2105-14-7.

55. Becht, E., Giraldo, N.A., Lacroix, L., Buttard, B., Elarouci, N., Petitprez, F., Selves, J., Laurent-Puig, P., Sautès-Fridman, C., Fridman, W.H., and de Reyniès, A. (2016). Estimating the population abundance of tissue-infiltrating immune and stromal cell populations using gene expression. Genome Biol. *17*, 218. https://doi.org/10.1186/s13059-016-1070-5.

56. Finotello, F., Mayer, C., Plattner, C., Laschober, G., Rieder, D., Hackl, H., Krogsdam, A., Loncova, Z., Posch, W., Wilflingseder, D., et al. (2019). Molecular and pharmacological modulators of the tumor immune contexture revealed by deconvolution of RNA-seq data. Genome Med. *11*, 34. https://doi.org/10.1186/s13073-019-0638-6.

57. Charoentong, P., Finotello, F., Angelova, M., Mayer, C., Efremova, M., Rieder, D., Hackl, H., and Trajanoski, Z. (2017). Pan-cancer Immunogenomic Analyses Reveal Genotype-Immunophenotype Relationships and Predictors of Response to Checkpoint Blockade. Cell Rep. *18*, 248–262. https://doi.org/10.1016/j.celrep.2016.12.019.

58. Newman, A.M., Liu, C.L., Green, M.R., Gentles, A.J., Feng, W., Xu, Y., Hoang, C.D., Diehn, M., and Alizadeh, A.A. (2015). Robust enumeration of cell subsets from tissue expression profiles. Nat. Methods *12*, 453–457. https://doi.org/10.1038/nmeth.3337.

59. Racle, J., de Jonge, K., Baumgaertner, P., Speiser, D.E., and Gfeller, D. (2017). Simultaneous enumeration of cancer and immune cell types from bulk tumor gene expression data. eLife *6*, e26476. https://doi.org/10.7554/eLife.26476.

60. Yang, W., Soares, J., Greninger, P., Edelman, E.J., Lightfoot, H., Forbes, S., Bindal, N., Beare, D., Smith, J.A., Thompson, I.R., et al. (2013). Genomics of Drug Sensitivity in Cancer (GDSC): a resource for therapeutic biomarker discovery in cancer cells. Nucleic Acids Res. *41*, D955–D961. https://doi.org/10.1093/nar/gks1111.

61. Li, R., Ferdinand, J.R., Loudon, K.W., Bowyer, G.S., Laidlaw, S., Muyas, F., Mamanova, L., Neves, J.B., Bolt, L., Fasouli, E.S., et al. (2022). Mapping single-cell transcriptomes in the intra-tumoral and associated territories of kidney cancer. Cancer Cell *40*, 1583–1599.e10. https://doi.org/10.1016/j.ccell.2022.11.001.

62. Wu, R., Guo, W., Qiu, X., Wang, S., Sui, C., Lian, Q., Wu, J., Shan, Y., Yang, Z., Yang, S., et al. (2021). Comprehensive analysis of spatial architecture in primary liver cancer. Sci. Adv. *7*, eabg3750. https://doi.org/10.1126/sciadv.abg3750.

63. Liu, Y., Xun, Z., Ma, K., Liang, S., Li, X., Zhou, S., Sun, L., Liu, Y., Du, Y., Guo, X., et al. (2023). Identification of a tumour immune barrier in the HCC microenvironment that determines the efficacy of immunotherapy. J. Hepatol. *78*, 770–782. https://doi.org/10.1016/j.jhep.2023.01.011.

64. Wu, S.Z., Al-Eryani, G., Roden, D.L., Junankar, S., Harvey, K., Andersson, A., Thennavan, A., Wang, C., Torpy, J.R., Bartonicek, N., et al. (2021). A single-cell and spatially resolved atlas of human breast cancers. Nat. Genet. *53*, 1334–1347. https://doi.org/10.1038/s41588-021-00911-1.

65. Engblom, C., Thrane, K., Lin, Q., Andersson, A., Toosi, H., Chen, X., Steiner, E., Lu, C., Mantovani, G., Hagemann-Jensen, M., et al. (2023). Spatial transcriptomics of B cell and T cell receptors reveals lymphocyte clonal dynamics. Science *382*, eadf8486. https://doi.org/10.1126/science.adf8486.

66. Wu, Y., Yang, S., Ma, J., Chen, Z., Song, G., Rao, D., Cheng, Y., Huang, S., Liu, Y., Jiang, S., et al. (2022). Spatiotemporal Immune Landscape of Colorectal Cancer Liver Metastasis at Single-Cell Level. Cancer Discov. *12*, 134–153. https://doi.org/10.1158/2159-8290.Cd-21-0316.

67. Galeano Niño, J.L., Wu, H., LaCourse, K.D., Kempchinsky, A.G., Baryiames, A., Barber, B., Futran, N., Houlton, J., Sather, C., Sicinska, E., et al. (2022). Effect of the intratumoral microbiota on spatial and cellular heterogeneity in cancer. Nature *611*, 810–817. https://doi.org/10.1038/s41586-022-05435-0.

68. Bergenstråhle, L., He, B., Bergenstråhle, J., Abalo, X., Mirzazadeh, R., Thrane, K., Ji, A.L., Andersson, A., Larsson, L., Stakenborg, N., et al. (2022). Super-resolved spatial transcriptomics by deep data fusion. Nat. Biotechnol. *40*, 476–479. https://doi.org/10.1038/s41587-021-01075-3.

69. Ren, Y., Huang, Z., Zhou, L., Xiao, P., Song, J., He, P., Xie, C., Zhou, R., Li, M., Dong, X., et al. (2023). Spatial transcriptomics reveals niche-specific enrichment and vulnerabilities of radial glial stem-like cells in malignant gliomas. Nat. Commun. *14*, 1028. https://doi.org/10.1038/s41467-023-36707-6.

70. Causer, A., Tan, X., Lu, X., Moseley, P., Teoh, S.M., Molotkov, N., McGrath, M., Kim, T., Simpson, P.T., Perry, C., et al. (2023). Deep spatial-omics analysis of Head & Neck carcinomas provides alternative therapeutic targets and rationale for treatment failure. NPJ Precis. Oncol. *7*, 89. https://doi.org/10.1038/s41698-023-00444-2.

71. Pavel, I., Irina, L., Tatiana, G., Denis, P., Philipp, K., Sergei, K., and Evgeny, D. (2023). Comparison of the Illumina NextSeq 2000 and GeneMind Genolab M sequencing platforms for spatial transcriptomics. BMC Genom. *24*, 102. https://doi.org/10.1186/s12864-023-09192-w.

OMTN, Volume *36*

Supplemental information

Pan-cancer neural regulation pattern with implications for patient stratification and immunotherapy

Yueying Gao, Jiyu Guo, Wenyi Yang, Kefan Liu, Qingyi Yang, Si Li, Dezhong Lv, Yangyang Cai, Kang Xu, Weiwei Zhou, Qinghua Jiang, Juan Xu, Yongsheng Li, and Haoxiu Sun

Supplemental Figures

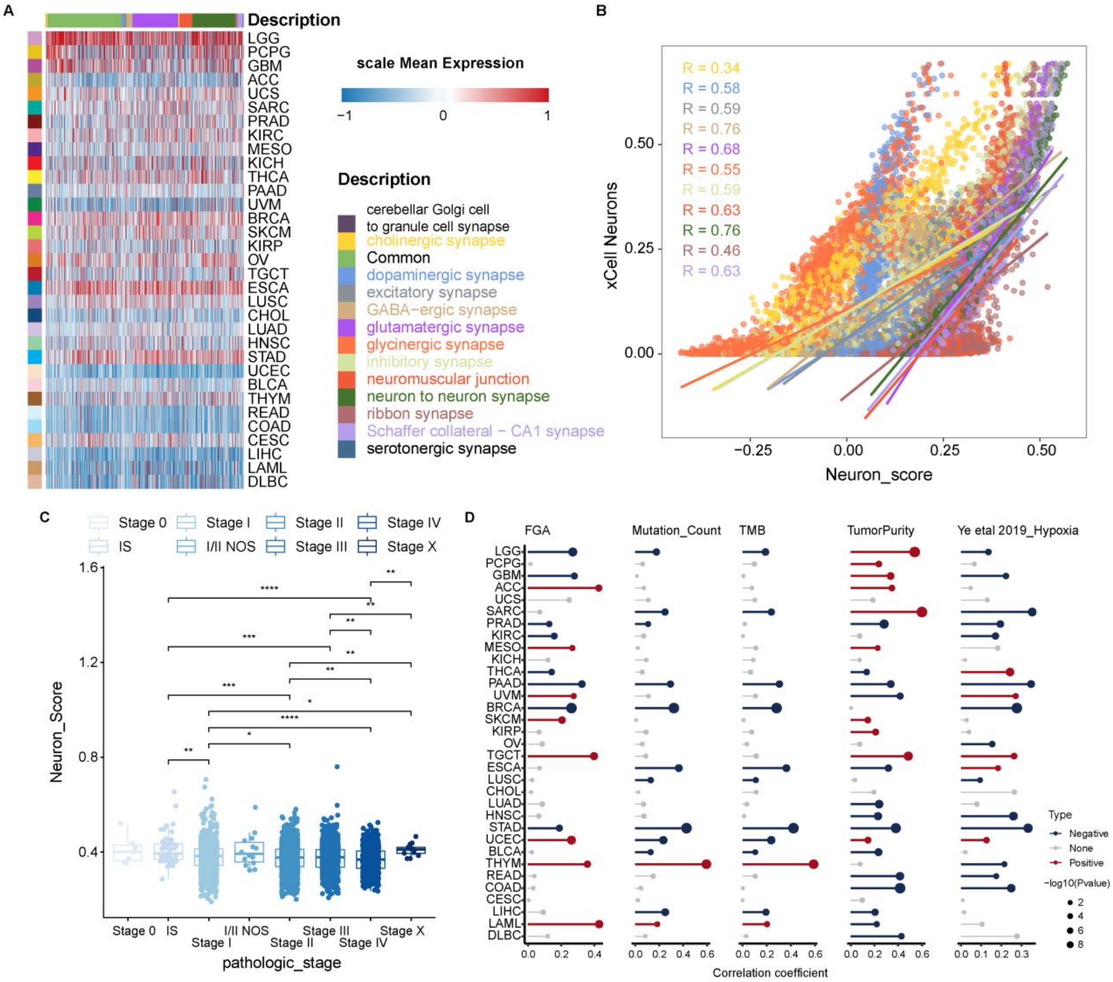

Figure S1. Correlations between the activities of neural signal pathways and clinical features. (A) Heatmap showing the expressions of neural-related genes across 33 cancer types. (B) Scatter plot showing the correlation between neural signals and the abundance of neuronal cells obtained based on xCell algorithm. (C) Boxplot showing the neural signal activitiesacross different stages. (D) Dotplot showing the correlation between the activity of neural signals and clinical parameters, such as FGA, mutation count and TMB.

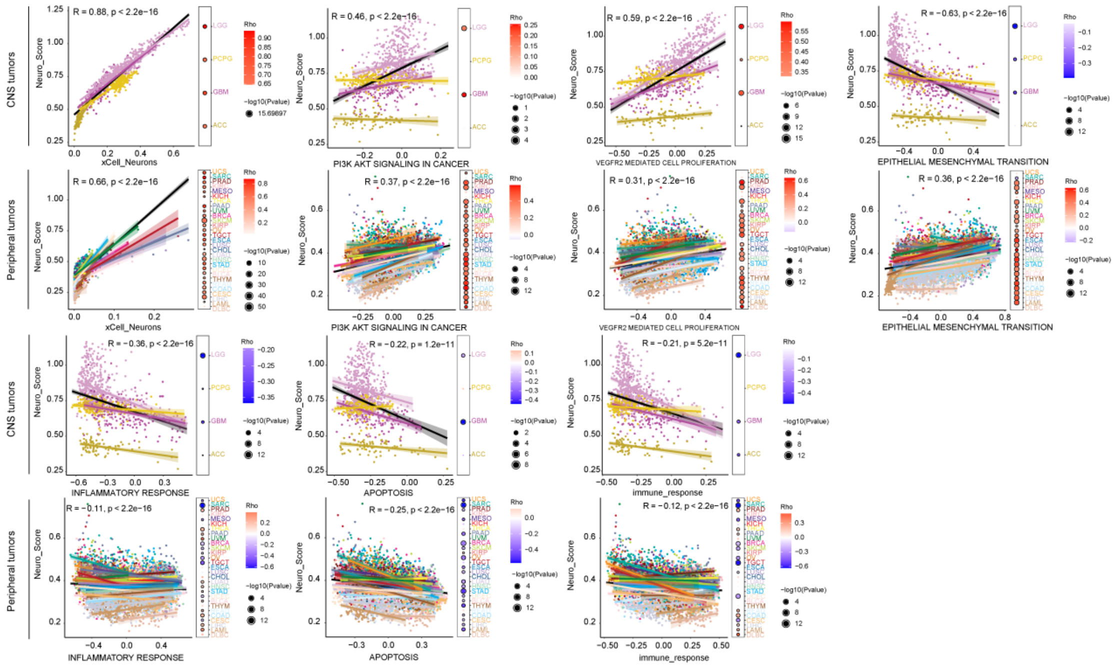

Figure S2. Correlation between the activities of neural signals and TME-related pathways in diverse cancer types. Scatter plot (left) colored by cancer types showing the distribution of patients. Dot plot (right) showing the correlation between neural signals and TME-related pathways. Red represents positive correlation, blue represents negative correlation. The size of the dot represents -log10 (Pvalue).

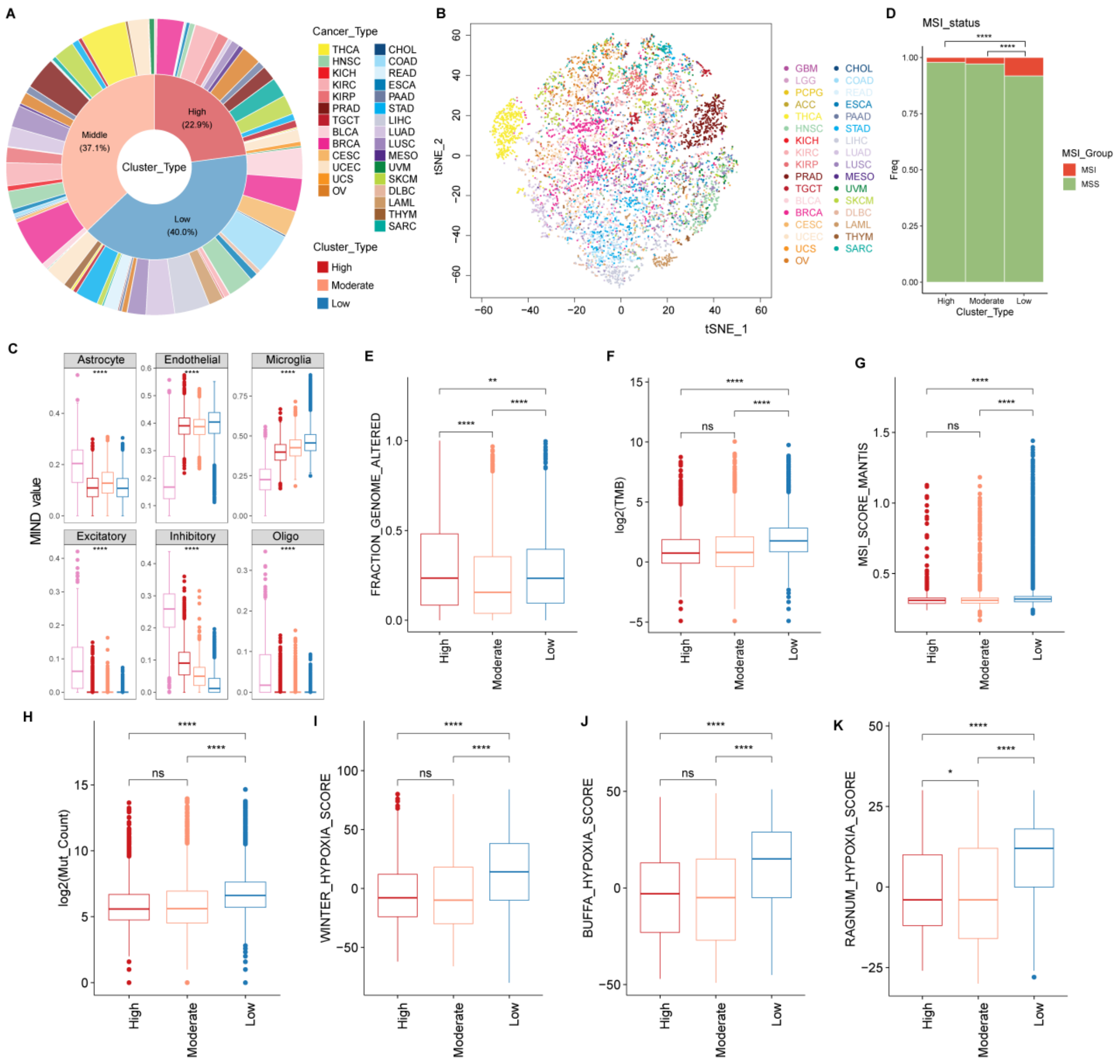

Figure S3. Clinical relevance of molecular subtypes classified by neural signal activity. (A) Circular diagram showing the distribution of cancer types in three molecular subtypes. (B)The t-SNE plot showing the distribution of cancer types. (C) Barplot showing the proportion of patients with microsatellite instability (MSI) or microsatellite stability (MSS) across three subtypes. (D) Boxplot of six cell types derived from deconvolution method across diverse molecular subtypes. (E-K) Boxplot showing clinical features across three subtypes, including FGA (E), TMB (F), MSI score (G), mutation count (H), hypoxia score (I-K).

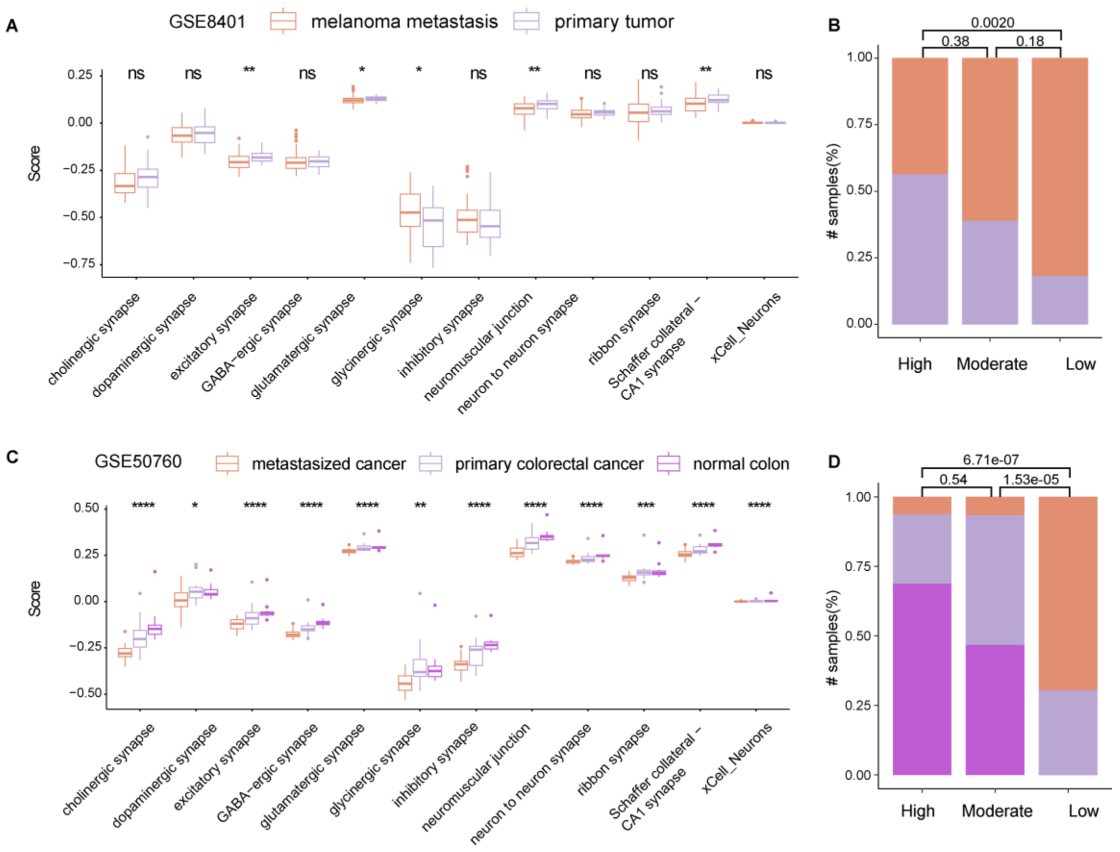

Figure S4. Landscape of metastasis across molecular subtypes. (A-B) Boxplot and Barplot showing the distribution of neural signals in metastasis and primary tumors in melanoma cohort. (C-D) Boxplot and Barplot showing the distribution of neural signals in metastasis and primary tumors in colorectal cohort.

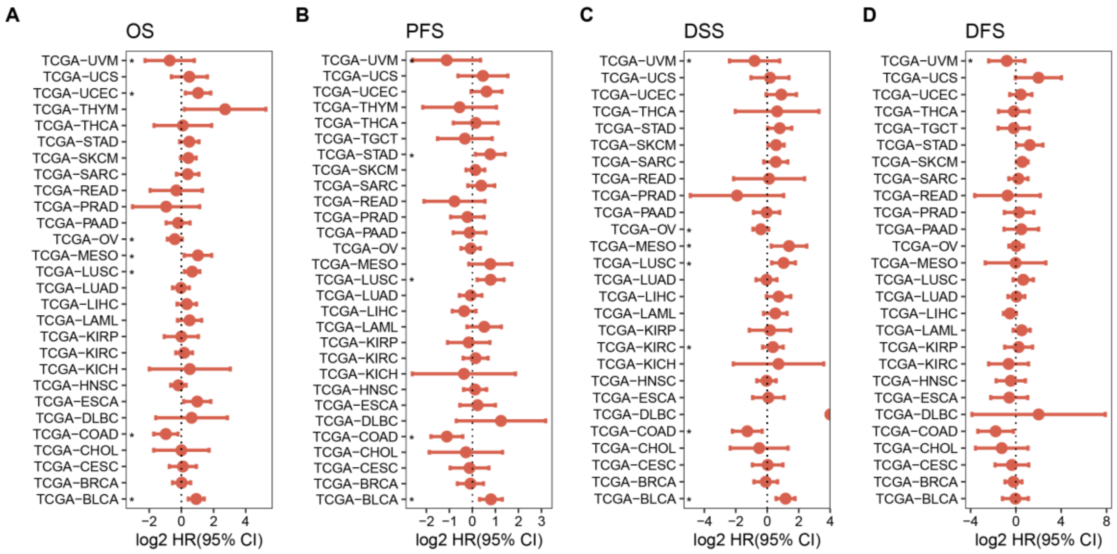

Figure S5. Survival analysis of diverse cancers. Survival analysis was performed to evaluate prognostic value (A, OS, overall survival; B, PFS, progression-free survival; C, DSS, disease-specific survival; D, DFS, disease-free survival) of diverse cancers.

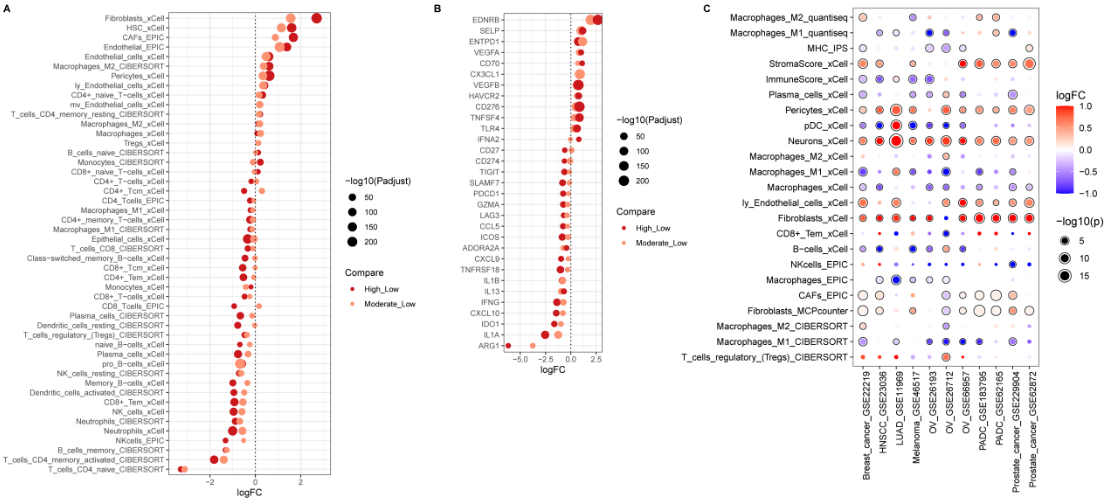

Figure S6. Differences of immunomodulators between molecular subtypes. The dot plot showing the differential changes in abundances of cell types (A) and expressions of immune regulatory gene (B) related to TIME. (C) The relationship between neural signals and TIME in other cohorts. Red represents upregulation in the neural-high group, while blue represents upregulation in the neural-low group. The size of the points are related to the p-value.

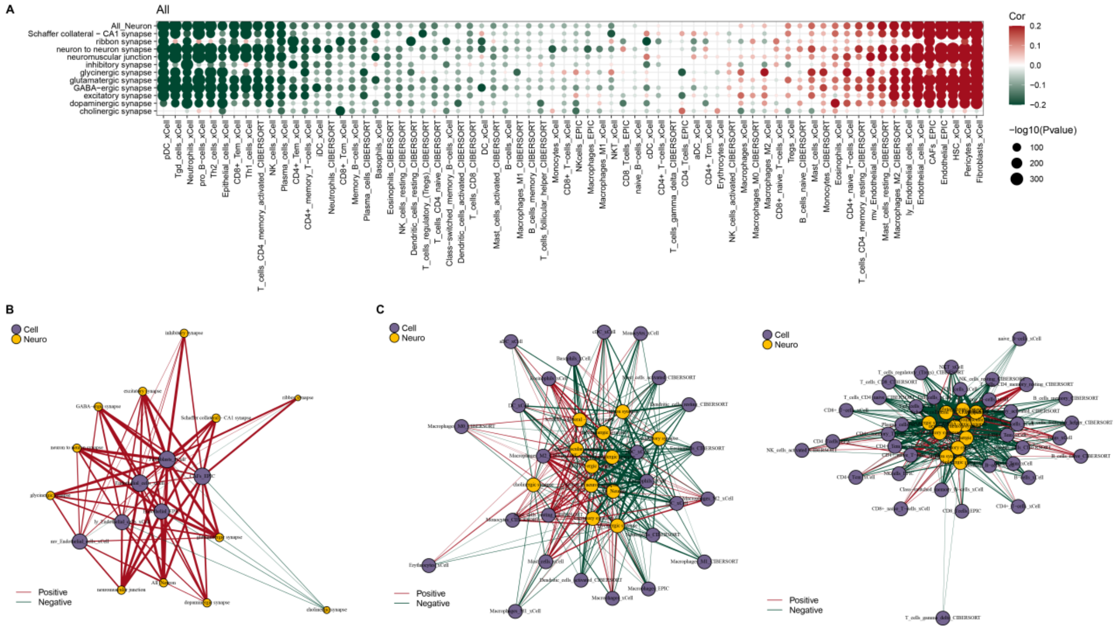

Figure S7. The correlation between neural signals and TIME-related cell types. (A) The dot plot showing the correlation between the activities of neural signals and abundances of TIME-related cell types. (B) The networks exhibiting the association between neural signals and oncogenesis-related cell types. (C) The networks exhibiting the association between neural signals and immune-related cell types, such as myeloid cell (left) and lymphoid cells (right). Red represents significant positive correlation, green represents significant negative correlation. Yellow represents neural signals, while purple represents cancer or immune related cell types.

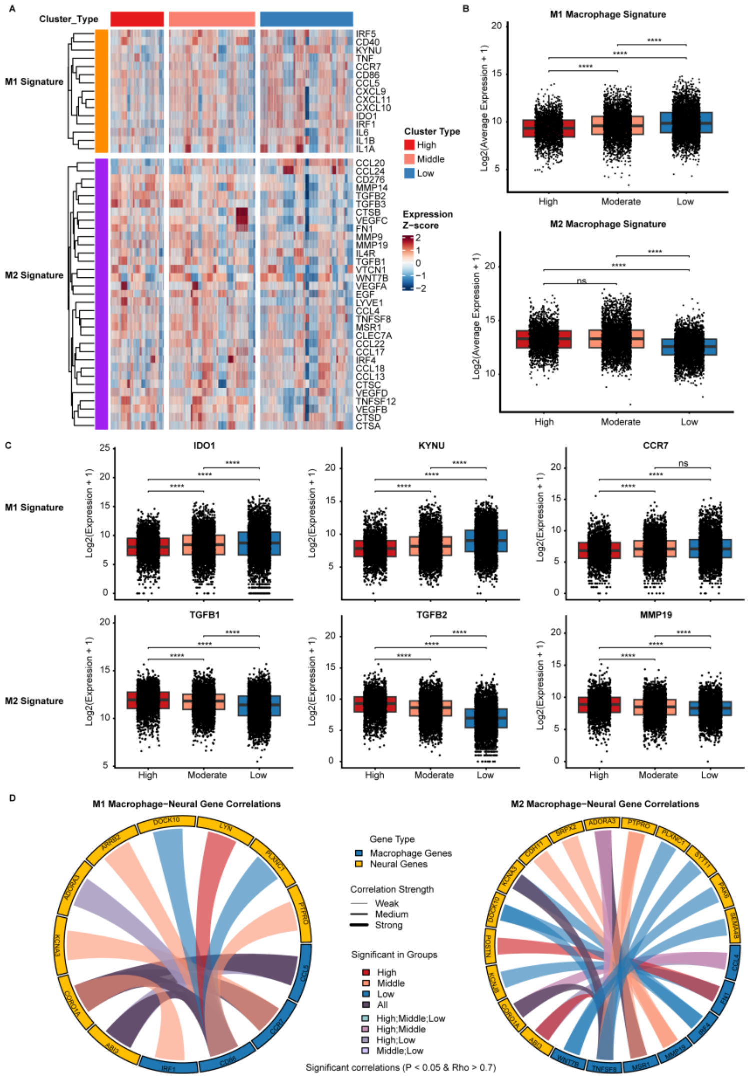

Figure S8. Correlation between neural signals and macrophage subtypes. (A) Heatmap showing the expression of marker genes related macrophage polarization. (B) Boxplot displaying macrophage signature scores across diverse neural subtypes. (C) Boxplot showing the expression distribution of macrophage signatures in different subtypes. (D) Circos plot showing the correlation between neural signals signatures and macrophage signatures. Blue indicates neural signals signatures, and yellow indicates macrophage signatures.

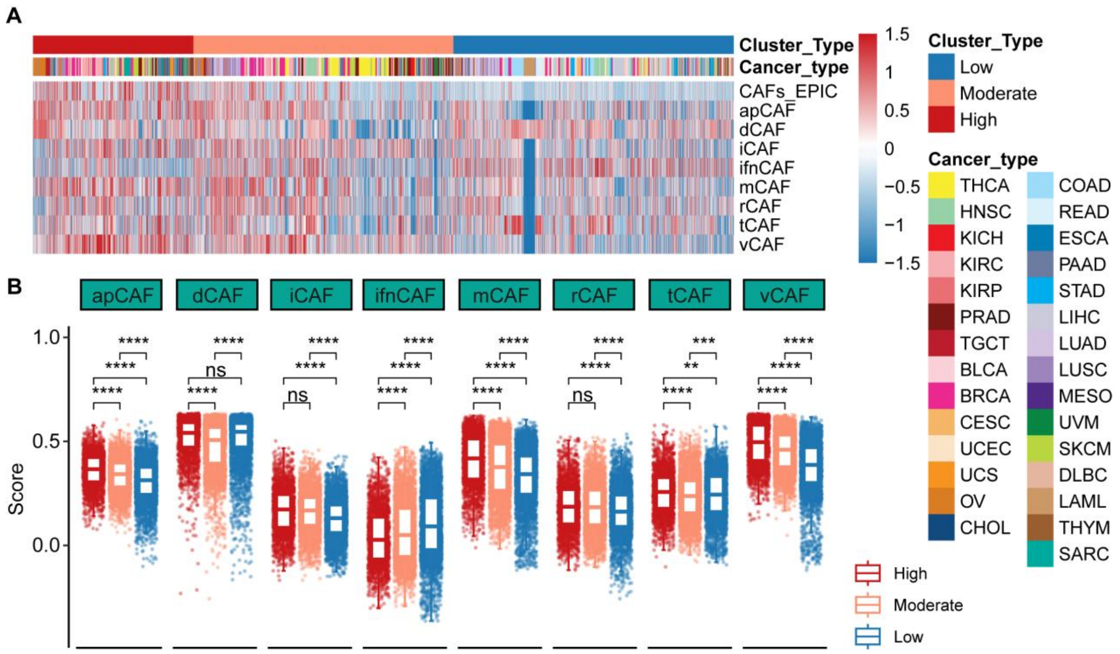

Figure S9. Differences of CAFs among neural signals-related molecular subtypes . (A) Heatmap showing the enrichment score of different CAF subtype signatures. (B) Boxplot displaying the distribution of different CAF signatures among molecular subtypes classified by neural signals. apCAF, antigen-presenting CAFs; dCAF, dividing CAFs; iCAF, inflammatory CAFs; ifnCAF, interferon response CAFs; mCAF, matrix CAFs;rCAF, reticular-like CAFs;tCAF, tumor-like CAFs;vCAF, vascular or vessel-associated CAFs.

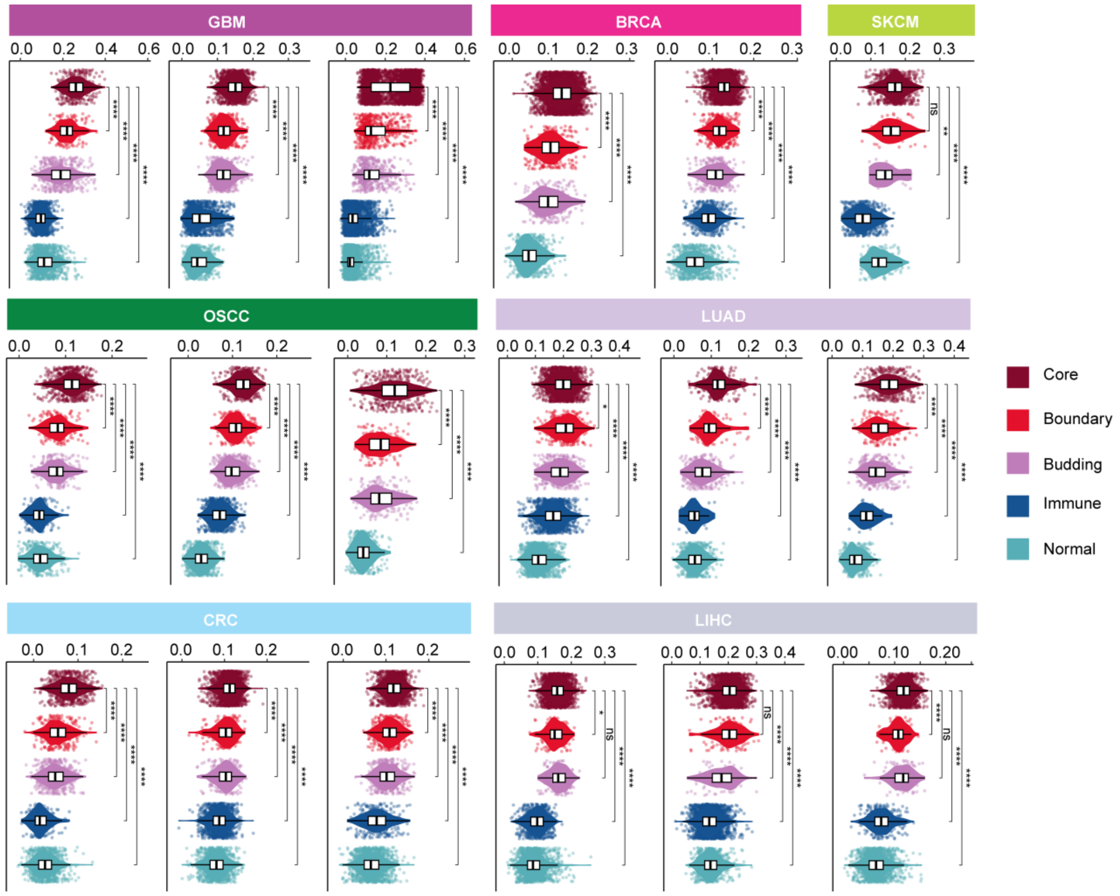

Figure S10. Neural signals activity in different regions based on the spatial transcriptome. VlnpIot exhibiting the distribution of neural signal activity across different regions in various cancer types, associated with Figure 4b. Wilcoxon rank sum test, P>0.5, ns; p<0.5, *; p<0.01, **; p<0.001, ***; p<0.0001, ****.

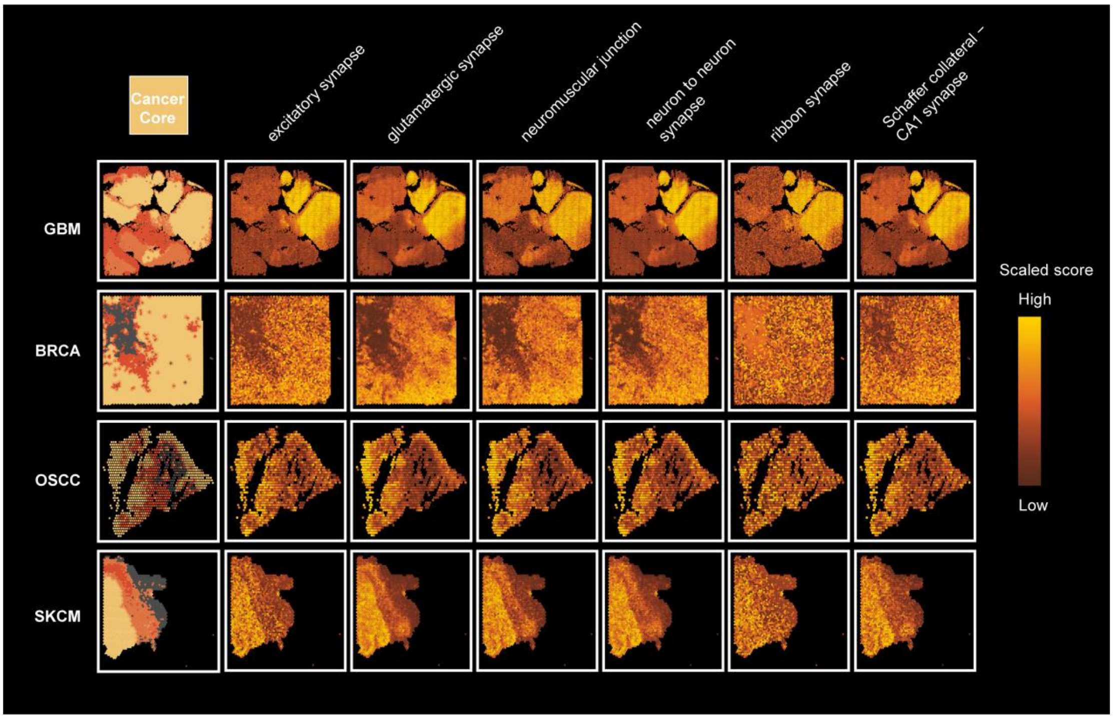

Figure S11. The activities of neural signals-related pathways in tumor core regions. The SpatialFeaturePlot displaying the activities of neural signals-related pathways in tumor core regions of GBM, BRCA, OSCC and SKCM.

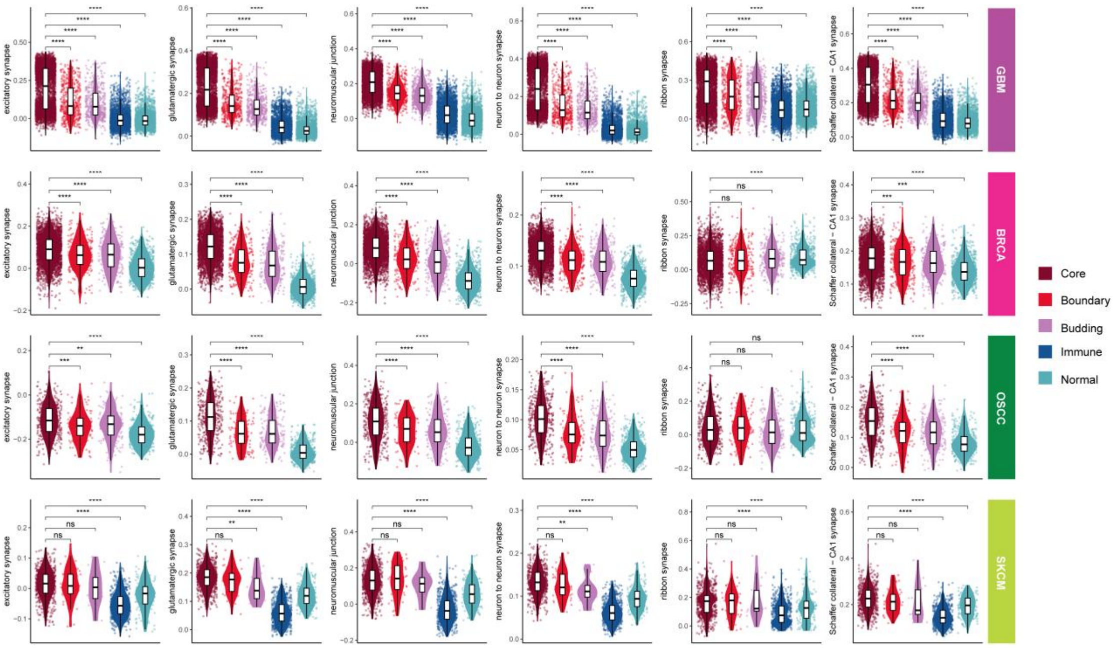

Figure S12. The activities of neural signals-related pathways in different regions. Vlnplot comparing the activities of neural signals-related pathways across different regions of various cancer types, associated with Figure S9. Wilcoxon rank sum test, P>0.5, ns; p<0.5, *; p<0.01, **; p<0.001, ***; p<0.0001, ****.

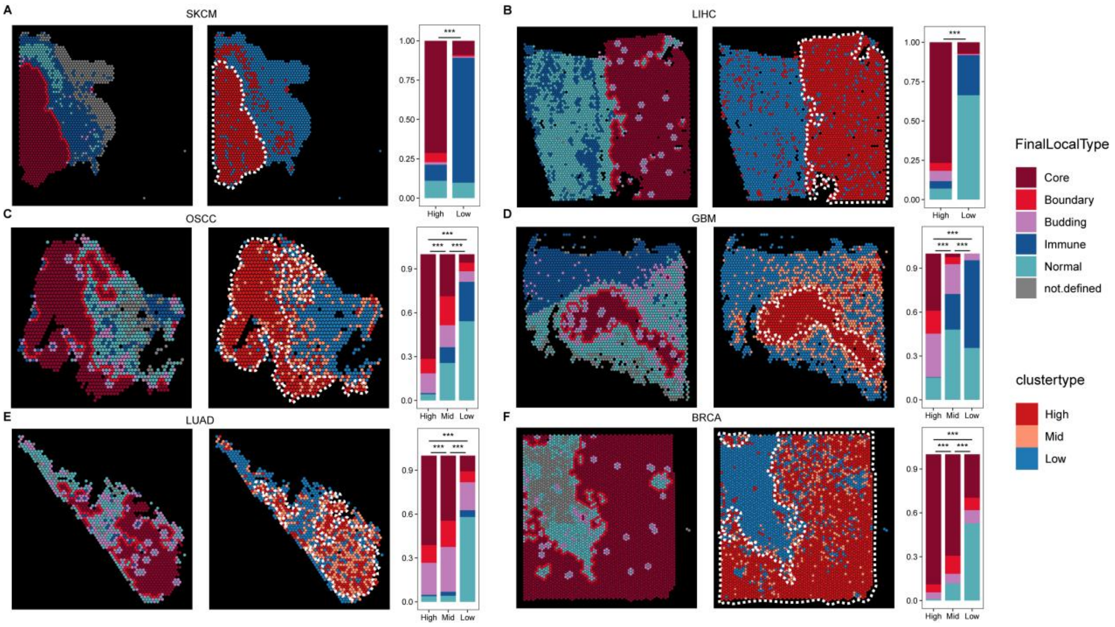

Figure S13. The association between neural signals-related molecular subtypes and diverse regions. The SpatialFeaturePlot comparing the distribution of neural signals-related molecular subtypes (middle) with diverse regions (left) of SKCM (A), LIHC (B), OSCC (C), GBM (D), LUAD (E) and BRCA (F). The bar plots (right) showing the proportion of spots from different regions in different subtypes.

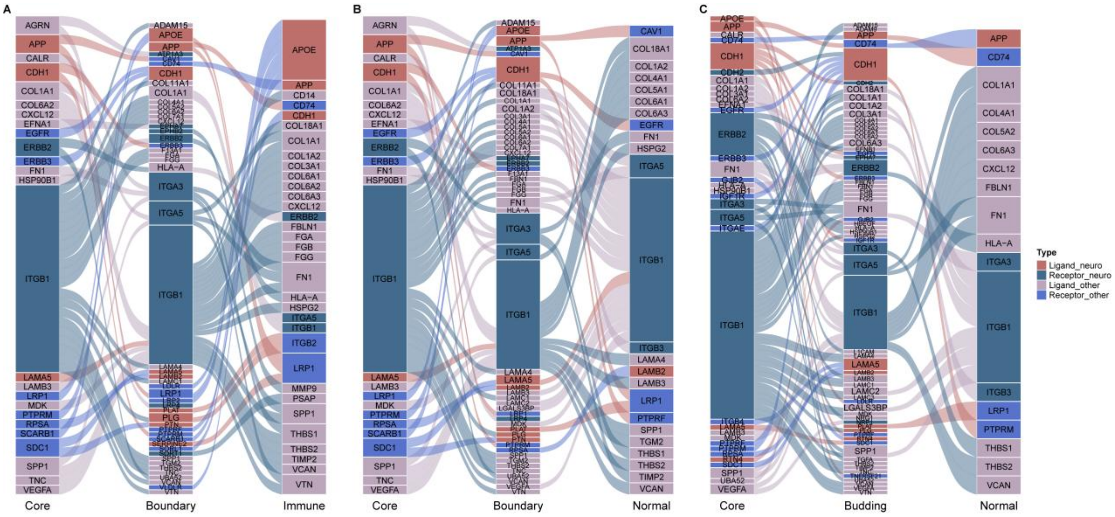

Figure S14. Ligand-receptor interactions between different regions. The sankey plots displaying the interaction of neural signals-related ligands or receptors and their paired receptors/ligands between malignant regions and non-malignant regions. Deep red represents neural-related ligands, and deep blue represents neural-related receptors.

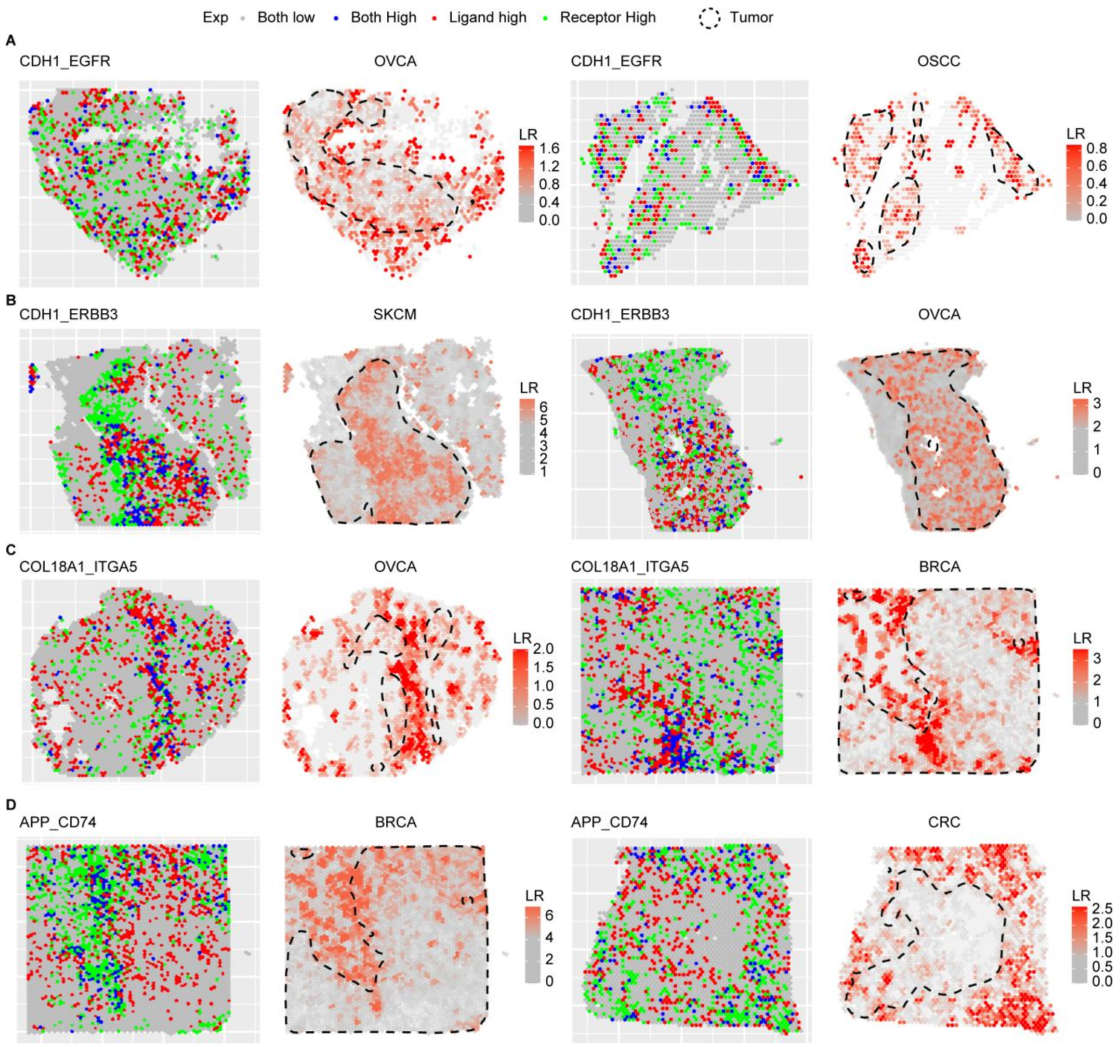

Figure S15. Spatial localization of Ligand-Receptor pairs. The scatter diagram displaying the expression distribution of the Ligand-Receptor pairs, such as *CDH1-EGFR* (A), *CDH1-ERBB3* (B), *COL18A1-ITGA5* (C) and *APP-CD74* (D).

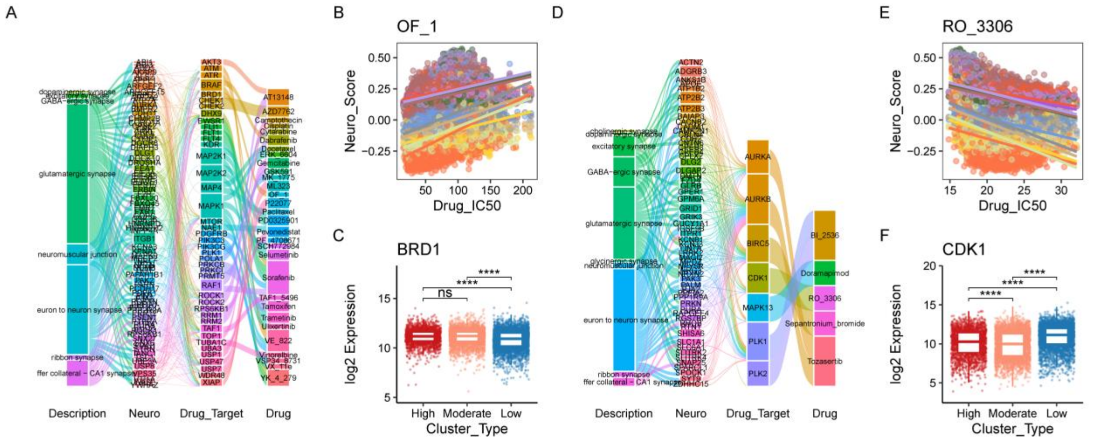

Figure S16. The correlation between neural signals and drug targets. (A) Sankey diagram illustrating the interaction between neural signals and the targets of positively correlated drugs. (B) The scatter plot showing shows the positive correlation between neural signals and IC50 values of OF-1. (C) Box plot displaying the expression distribution of OF-1 target *BRD1* in diverse neural subtypes. (D) Sankey diagram illustrating the interaction between neural signals and the targets of negatively correlated drugs. (E) The scatter plot showing shows the negative correlation between neural signals and IC50 values of RO-3306. (F) Box plot displaying the expression distribution of RO-3306 target *CDK1* in diverse neural subtypes.

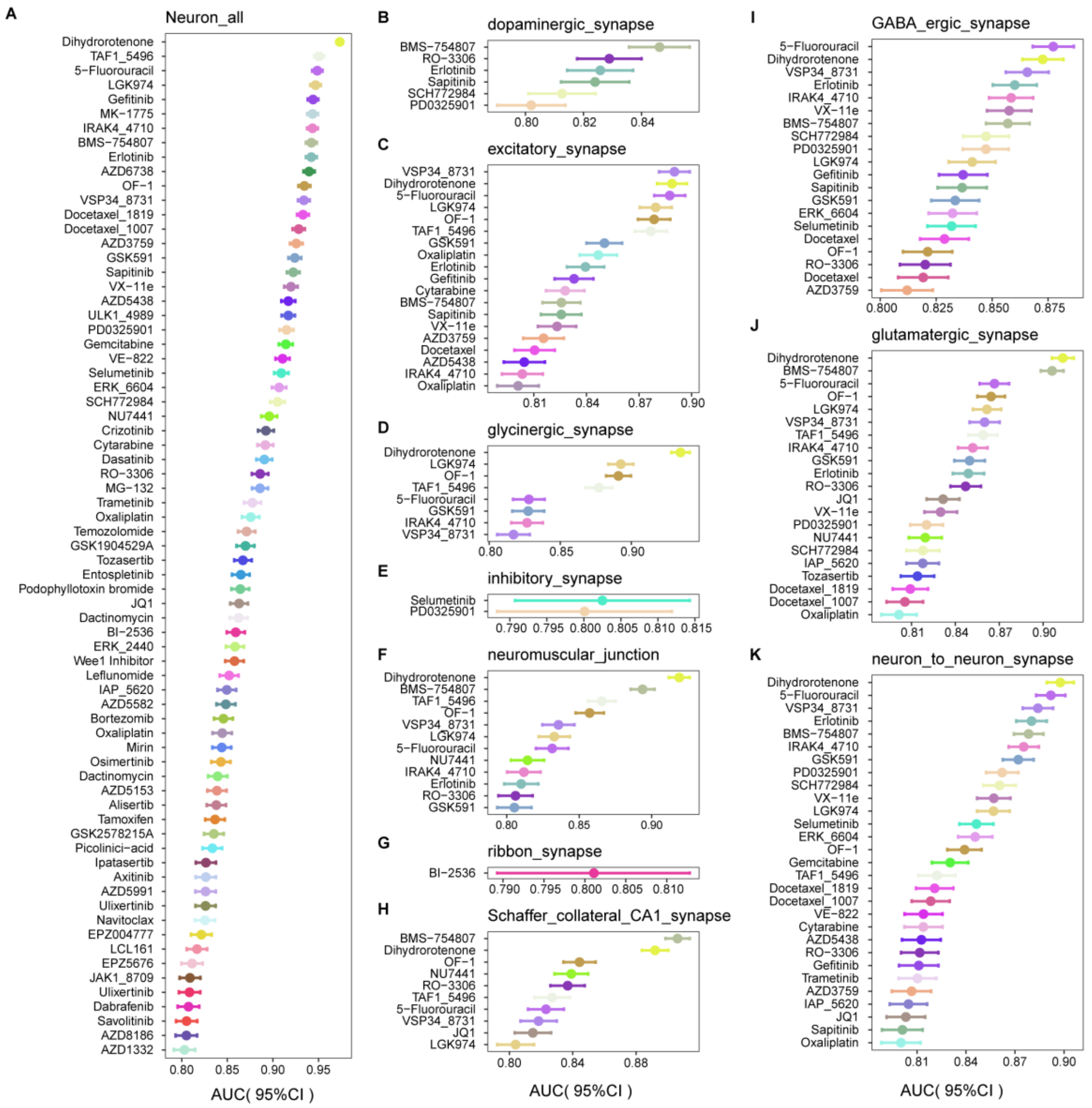

Figure S17. The efficacy of neural signals-related features in predicting drug sensitivity. The dot plots displaying the accuracy of neural signals-related features in predicting drug sensitivity via the area under the receiver operating characteristic curve (AUC). Only results with AUC greater than 0.8 were displayed.

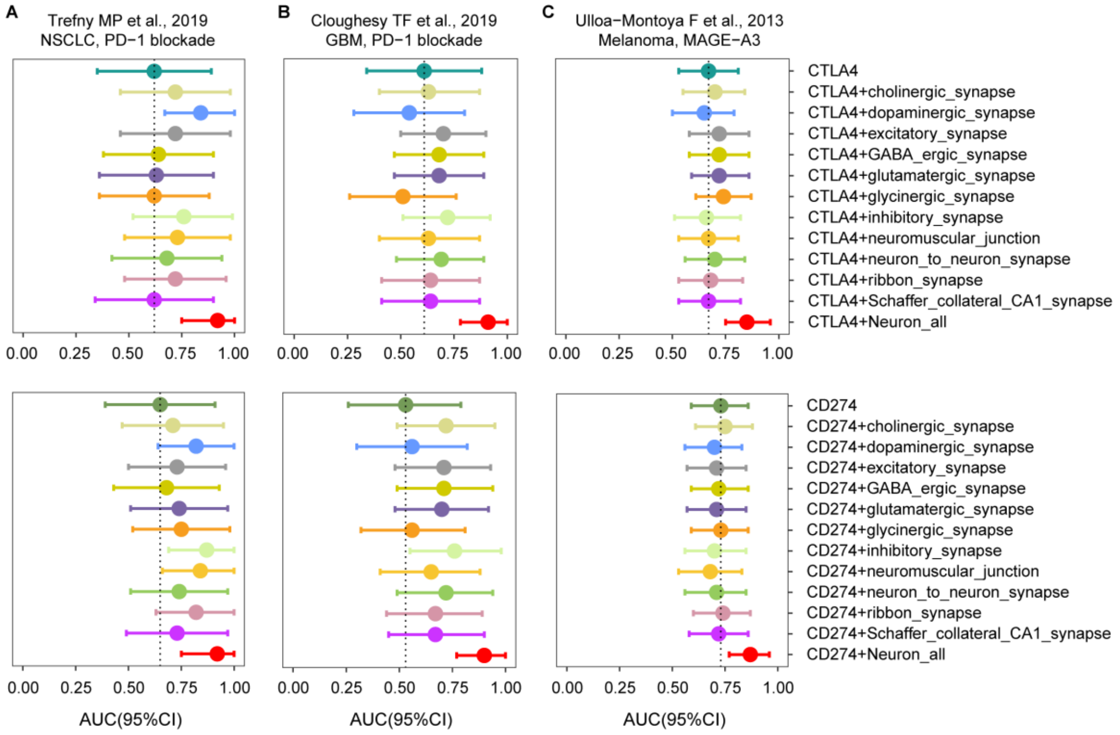

Figure S18. The efficacy of immune check points and combained neural signals-related features in predicting immunotherapy response. The dot plots displaying the accuracy of immune check points and combained neural signals-related features in predicting immunotherapy response via the area under the receiver operating characteristic curve (AUC), using NSCLC, GBM and Melanom cohorts.

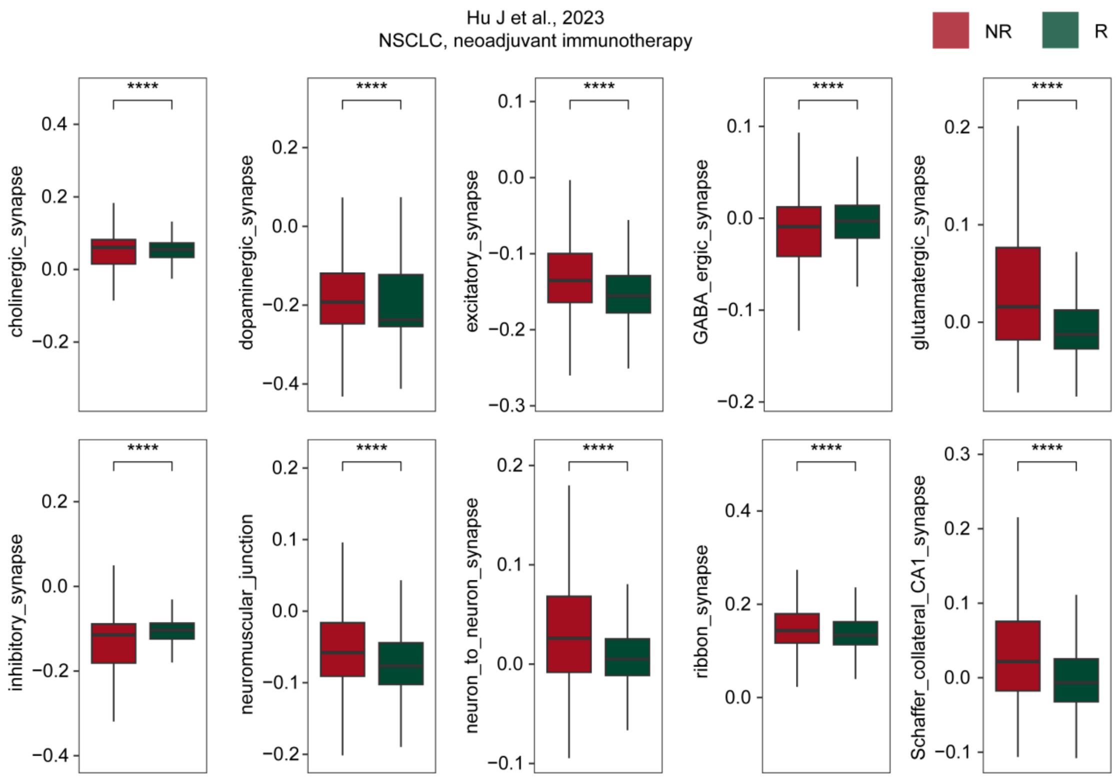

Figure S19. Activities of neural signals in responsive group and non-responsive group. The boxplot displaying the distribution of activities of neural signals in responsive group and non-responsive group using NSCLC single-cell cohort.

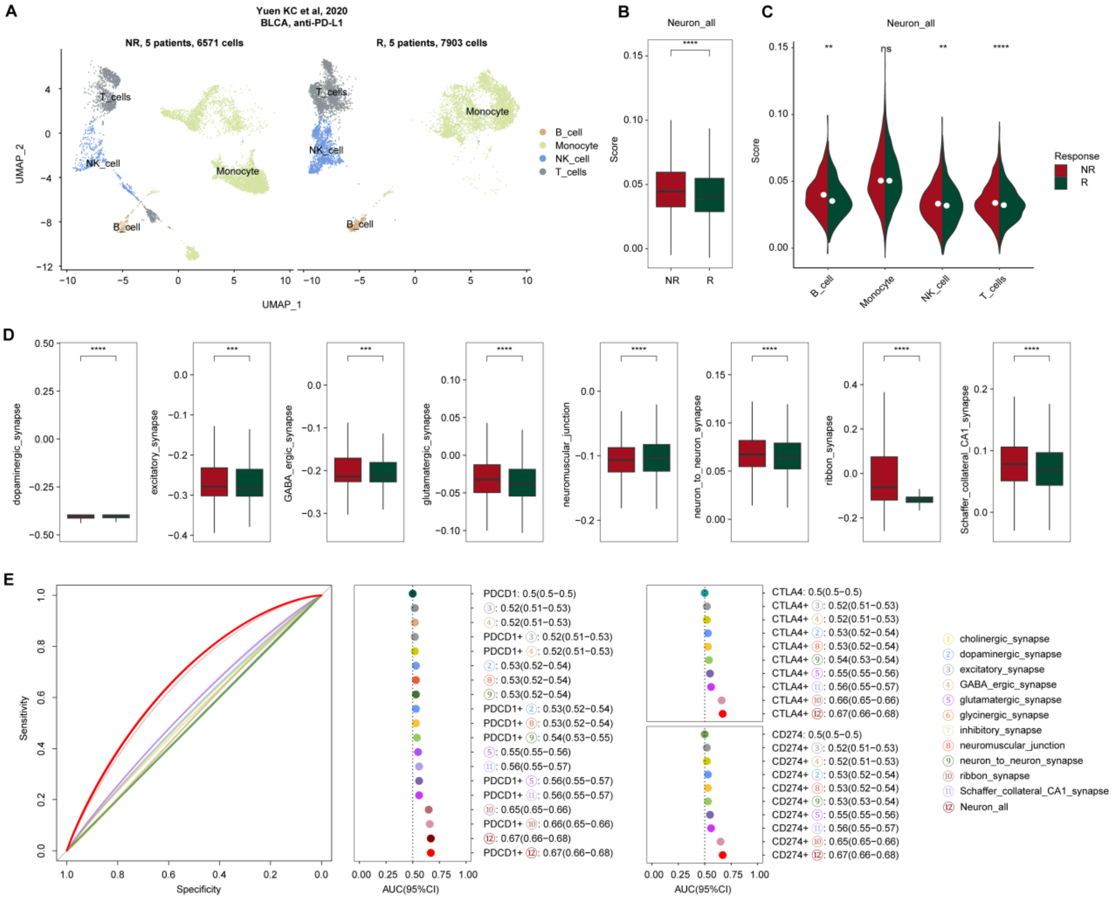

Figure S20. The performance of neural signals in predicting immune therapy response using BLCA single-cell cohort. (A) The umap showing cell types and number of cells in responsive group and non-responsive group. (B-D) The boxplot displaying he distribution of activities of neural signals in responsive group and non-responsive group, as well as in diverse cell types. (E) ROC curve (left) evaluating the ability of immune check points and combained neural signals-related features to predict immune therapy response. The dot plots (right) displaying the accuracy in predicting immunotherapy response via AUC value.

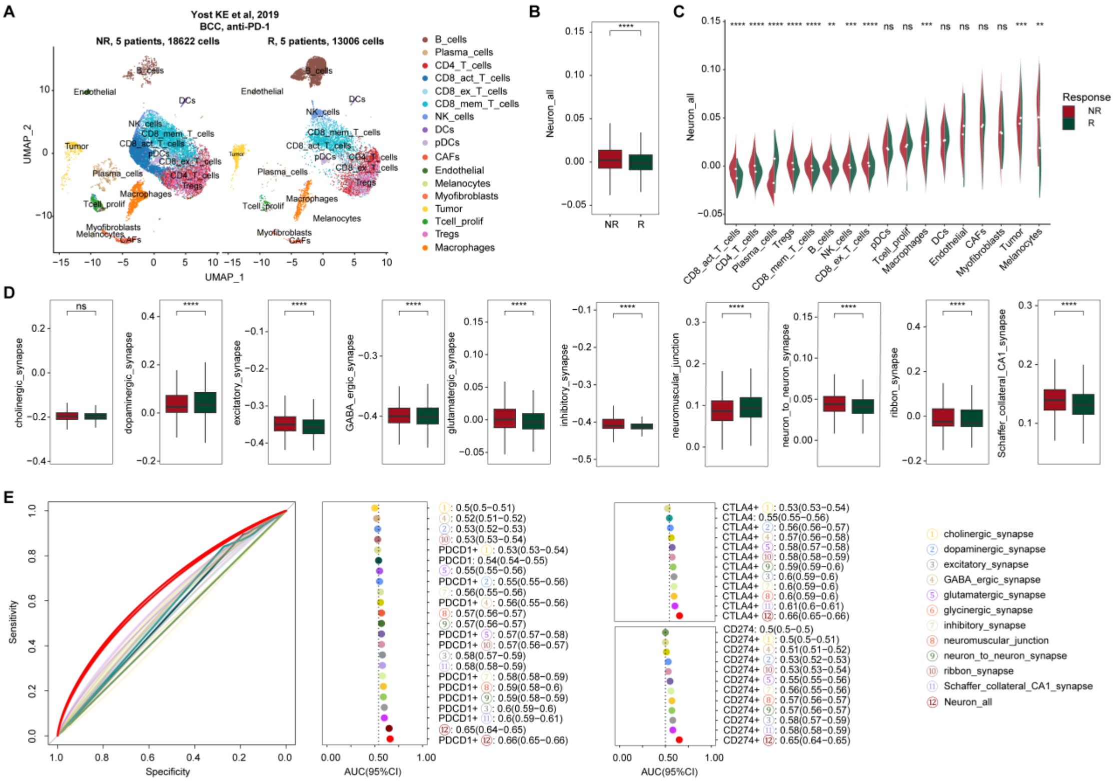

Figure S21. The performance of neural signals in predicting immune therapy response using BCC single-cell cohort. (A) The umap showing cell types and number of cells in responsive group and non-responsive group. (B-D) The boxplot displaying he distribution of activities of neural signals in responsive group and non-responsive group, as well as in diverse cell types. (E) ROC curve (left) evaluating the ability of immune check points and combained neural signals-related features to predict immune therapy response. The dot plots (right) displaying the accuracy in predicting immunotherapy response via AUC value.

Supplemental Tables

Table S1. Gene members of neural signal pathways.

Table S2. The genes involved in cancer or immune-related pathways.

Table S3. Correlations between activities of neural signals and abundances of TIME-related cell.

Table S4. The list of immune regulatory factors.

Table S5. DE results of immune-related factors between subtypes.

Table S6. AUC and 95%CI of neural signals-related signatures in predicting drug sensitivity.
